# Construction of highly functionalized carbazoles *via* condensation of an enolate to a nitro group†
†Electronic supplementary information (ESI) available: Experimental procedures, characterization data, ^1^H NMR and ^13^C NMR spectra for synthesized compounds. X-ray structure and data for **7a**. CCDC 1046362. For ESI and crystallographic data in CIF or other electronic format see DOI: 10.1039/c5sc02407b


**DOI:** 10.1039/c5sc02407b

**Published:** 2015-09-16

**Authors:** Tej Narayan Poudel, Yong Rok Lee

**Affiliations:** a School of Chemical Engineering , Yeungnam University , Gyeongsan 712-749 , Republic of Korea . Email: yrlee@yu.ac.kr ; Fax: +82-53-810-4631 ; Tel: +82-53-810-2529

## Abstract

This paper describes a novel synthesis of highly functionalized and diverse carbazoles *via* transition-metal-free and mild base-promoted condensations of readily available 2-nitrocinnamaldehyde or 2-nitrochalcones with various β-ketoesters or 1,3-diaryl-2-propanones. The method selectively forms four bonds by the intramolecular conjugate addition of an enolate to the enal or chalcone bearing an *o*-nitro group. This group then undergoes *in situ* N–O bond cleavage under non-reductive conditions in a one-pot procedure. This protocol allows for the introduction of various functional groups at all positions of the newly formed aromatic ring of the carbazole moiety. The utility of this methodology is further illustrated by the concise synthesis of naturally occurring hyellazole and chlorohyellazole.

## Abstract 2

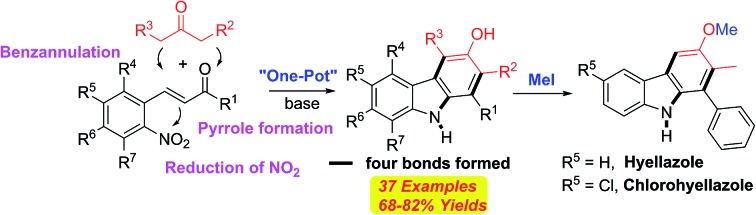
A transition-metal-free unique tandem annulation reaction has been developed for the synthesis of various functionalized 3-hydroxycarbazoles.

## Introduction

**Fig. 1:**
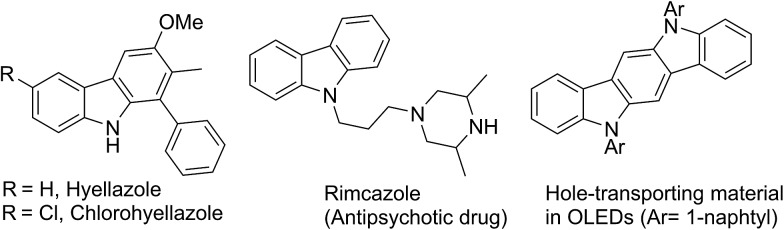
Selected naturally occurring, pharmaceutical, or electroactive carbazoles.

The carbazole framework is found in a wide range of bioactive natural products and pharmaceuticals (Fig. 1).^1^,^2^ These carbazole-containing molecules show antiviral,^3^ antimalarial,^4^ and antitumor activity.^5^ Some of them are currently being used as lead compounds for drug development.^6^ Carbazoles are also used as building blocks for the synthesis of functional materials, such as organic light-emitting diodes (OLED), because of their wide band gap, high luminescence efficiency, and allowing flexible modification of the parent skeleton.^7^,^8^

**Fig. 2:**
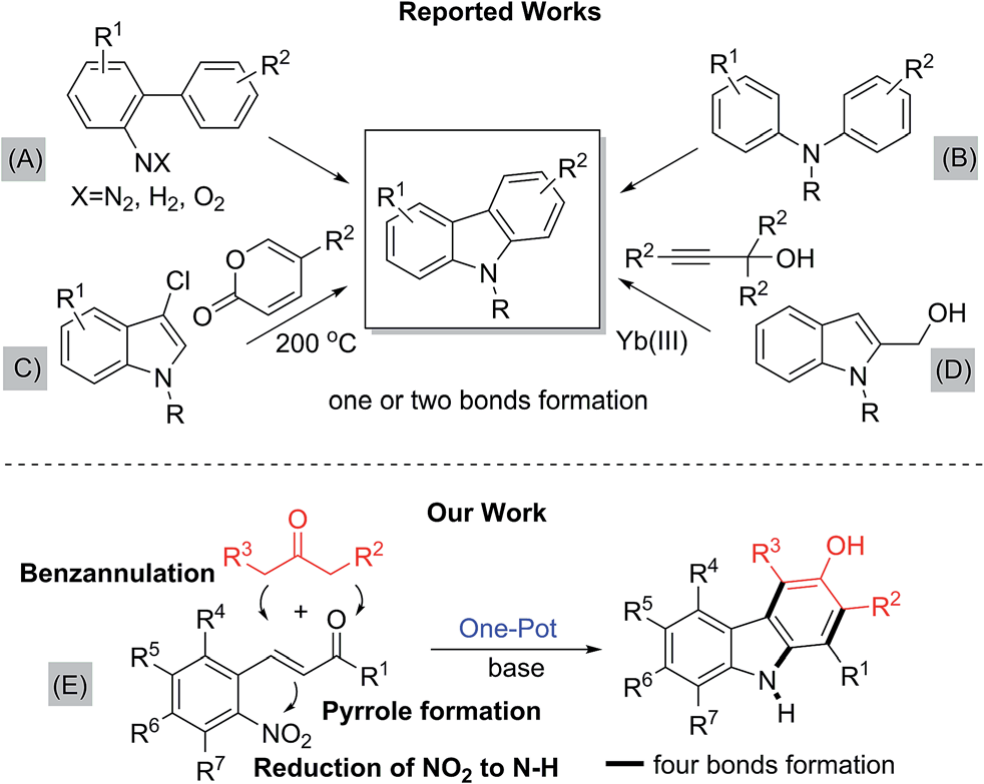
Diverse synthetic routes for carbazoles.

Owing to the importance and usefulness of these carbazole-based compounds, various approaches for their construction have been developed. The general and representative strategies can be classified into two main types depending on how the carbazole ring is constructed. The first strategy relies on the formation of a C–C or a C–N bond to construct the middle pyrrole ring starting from arene building blocks (methods A and B, Fig. 2).^9^–^16^ Also, the reaction of arynes with nitrosoarene and the nitrogenation of biphenyl halides have been reported.^17^ The second strategy involves the installation of a new aromatic ring onto functionalized indole derivatives *via* benzannulation (methods C and D, Fig. 2).^18^–^23^

Despite their own merits, most, if not all, of these methods suffer from certain drawbacks, including low tolerance of functionality, limited substrate scope, not-easily accessible starting materials, the necessity of complex and expensive transition-metal catalysts, and harsh reaction conditions. In particular, many existing methods require either highly elaborated biaryls or biarylamines to construct the central pyrrole moiety or pre-functionalized indole derivatives for benzannulation. Therefore, more environmentally benign and modular multi-bond forming approaches accommodating structurally simple building blocks as the feedstock are highly sought-after to improve on these shortcomings. In relation to the synthesis of 3-hydroxy carbazoles, iron-mediated reactions have also been reported.^24^ A recently-reported rhodium-catalyzed tandem annulation uses a new approach, where the [5 + 1] cycloaddition of 3-hydroxy-1,4-enynes with CO generates three bonds and two rings.^25^ Yet, even for this transformation, various 3-hydroxy-1,4-enyne reagents must be prepared by a multi-step route.

In this regard, the new approach, depicted in E, accommodating a novel double annulation through the consecutive construction of a pyrrole and a benzene moiety reflects further innovation (Fig. 2). A unique feature of the current reaction compared with all other reported pyrrole formations or benzannulations is the formation of the carbazole nitrogen atom by electrophilic attack on a nitro group rather than the use of an amine nucleophile. Herein, we describe a unique tandem annulation followed by N–O bond cleavage without any external reductant for the synthesis of various functionalized 3-hydroxycarbazoles from readily available 2-nitrocinnamaldehyde or 2-nitrochalcone and β-ketoesters or 1,3-diaryl-2-propanone.

## Results and discussion

First, the reaction of 2-nitrocinnamaldehyde (**1a**) and methyl 2-oxobutanoate (**2a**) was examined with several bases and solvents to optimize the reaction conditions (Table 1). The initial attempt with NaOMe (1 equiv.) in refluxing toluene for 12 h did not provide product **3a** (Table 1, entry 1), but produced an intractable mixture. With triethylamine (1 equiv.), product **3a** was also not formed (Table 1, entry 2), but with DBU (1 equiv.), **3a** was produced in 10% yield (Table 1, entry 3). Encouraged by this result, other bases were screened. With K_2_CO_3_ (1 equiv.) for 6 h, the yield of **3a** increased to 67% (Table 1, entry 4). The highest yield (81%) was achieved with 1.0 equivalent of Cs_2_CO_3_ in refluxing toluene for 4 h (Table 1, entry 5). Increasing the amount of Cs_2_CO_3_ to 1.5 equivalents (entry 6) or decreasing it to 0.1 equivalent (Table 1, entry 7) lowered the yield of **3a**. Based on these results, this transformation was found to be sensitive towards the base strength used. For example, strong bases like NaOMe (1 equiv.) or DBU (1 equiv.) provided very little or no desired product, while weak bases provided better yields. Among the screened bases, Cs_2_CO_3_ was superior in terms of both reaction time and yield for this reaction, probably due to its mild and optimum base strength.^26^ In two other nonpolar solvents (benzene or dichloroethane), **3a** was produced in 35 and 51% yield, respectively, whereas **3a** was not obtained in a more polar solvent, such as methanol, DMSO, or water (Table 1, entries 8–12). The structure of **3a** was established by spectroscopic analysis. The ^1^H NMR of **3a** showed a characteristic singlet of the OH group at *δ* 11.12 ppm and another broad singlet for the NH proton at *δ* 8.17 ppm. The ^13^C NMR showed the expected characteristic ester carbonyl carbon at *δ* 171.6 ppm and an aromatic carbon containing OH at *δ* 157.7 ppm. The structural confirmation of **3a** was further evidenced by X-ray crystallographic analysis of the related compound **7a** (see ESI†).

**Table 1:** Optimization of the reaction conditions for the synthesis of carbazole **3a**^*a*^

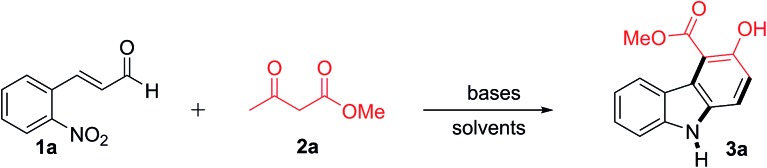				
Entry	Base	Solvent	Condition	Yield^*b*^ (%)
1	NaOMe (1 equiv.)	Toluene	Reflux, 12 h	0
2	TEA (1 equiv.)	Toluene	Reflux, 12 h	0
3	DBU (1 equiv.)	Toluene	Reflux, 12 h	10
4	K_2_CO_3_ (1 equiv.)	Toluene	Reflux, 6 h	67
**5**	**Cs**_**2**_ **CO**_**3**_ **(1 equiv.)**	**Toluene**	**Reflux, 4 h**	**81**
6	Cs_2_CO_3_ (1.5 equiv.)	Toluene	Reflux, 4 h	78
7	Cs_2_CO_3_ (0.1 equiv.)	Toluene	Reflux, 12 h	32
8	Cs_2_CO_3_ (1 equiv.)	Benzene	Reflux, 12 h	35
9	Cs_2_CO_3_ (1 equiv.)	DCE	Reflux, 12 h	51
10	Cs_2_CO_3_ (1 equiv.)	MeOH	Reflux, 12 h	0
11	Cs_2_CO_3_ (1 equiv.)	DMSO	Reflux, 12 h	0
12	Cs_2_CO_3_ (1 equiv.)	Water	Reflux, 12 h	0

^*a*^Reactions were conducted on a 1.0 mmol scale of **1a**.

^*b*^Isolated yield.

With the optimized conditions in hand, the generality of this reaction was explored by employing different β-ketoesters **2b–2i** (Table 2). Reaction of 2-nitrocinnamaldehyde (**1a**) with several β-ketoesters such as ethyl 2-oxobutanoate (**2b**), allyl 3-oxobutanoate (**2c**) and benzyl 3-oxobutanoate (**2d**), afforded the desired products **3b–3d** in 79, 82 and 77% yield, respectively. Moreover, the reactions of other β-ketoesters such as ethyl 3-oxopentanoate (**2e**), ethyl 3-oxohexanoate (**2f**), methyl 3-oxooctanoate (**2g**), methyl 3-oxododecanoate (**2h**), and methyl 3-oxo-4-phenylbutanoate (**2i**) provided the desired carbazoles **3e–3i** in 73–78% yield.

**Table 2:** Formation of carbazole **3b–3i** by the reaction of 2-nitrocinnamaldehyde **1a** with various β-ketoesters **2b–2i**^*a*^

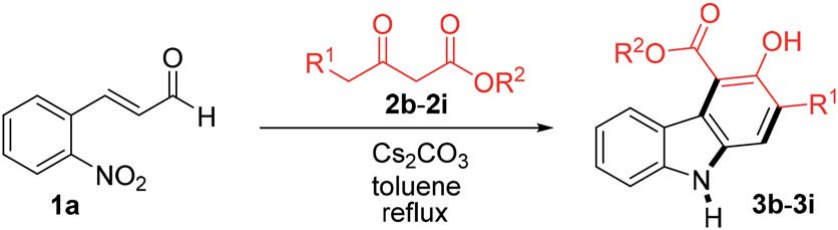
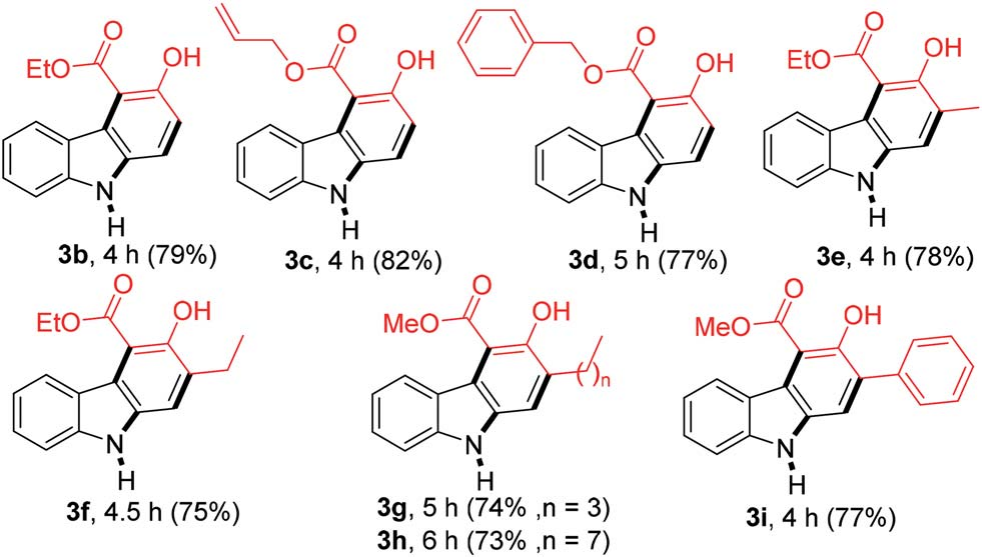

^*a*^Reactions were performed on a 1.0 mmol scale according to the standard conditions described in Table 1.

The scope of the reaction was further extended by employing a series of 2-nitrochalcones and β-ketoesters (Table 3). When 2-nitrochalcone **4a** was treated with allyl 3-oxobutanoate (**2c**), ethyl 3-oxopentanoate (**2e**) or 3-oxo-4-phenylbutanoate (**2i**) under optimized reaction conditions, the desired products **5a**, **5b** and **5c** were formed in 75, 73 and 78% yield respectively. Furthermore, 2-nitrochalcones **4b–4c**, bearing electron-donating or -withdrawing groups such as a methyl or bromo substituent on the 1-phenyl group, and β-ketoesters **2b**, **2d** and **2e** also provided the desired products **5d–5f** in 76, 75, and 70% yield, respectively. In addition, 2-nitrochalcones **4d–4f**, having electron-donating or -withdrawing groups such as a methoxy, bromo and chloro substituent on the 3-phenyl group, produced the expected carbazoles **5g–5k** in good yield (70–78%).

**Table 3:** Formation of carbazoles **5a–5k** from various 2-nitrochalcones (**4a–4f**) and several β-ketoesters (**2a–2e** and **2i**)^*a*^

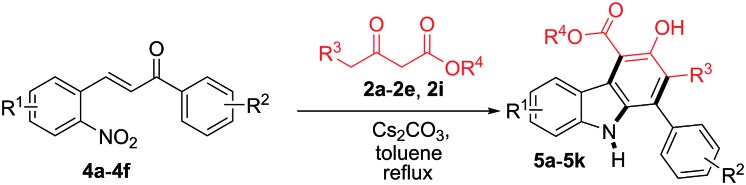
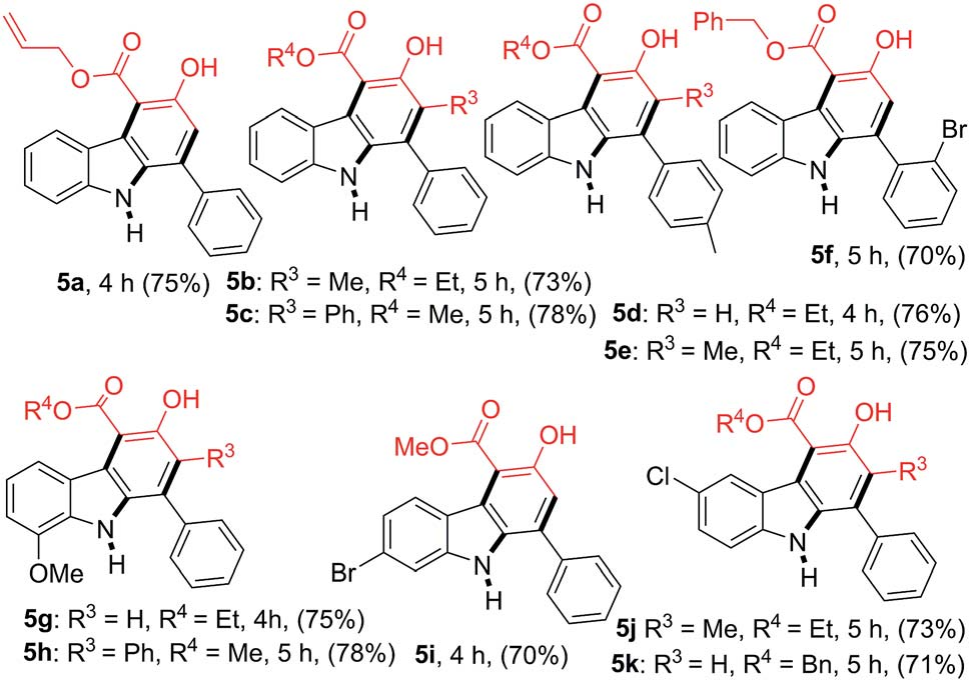

^*a*^Reactions were performed on a 1.0 mmol scale according to the standard conditions described in Table 1.

The reactions between 2-nitrocinnamaldehyde (**1a**) or one of the 2-nitrochalcones (**4a**, **4c**, **4d**, **4e** and **4f**) and 1,3-diarylpropan-2-ones **6a** and **6b** were examined to further demonstrate the versatility of this carbazole formation (Table 4). The reaction of **1a** with **6a** or **6b** in refluxing toluene for 4 h afforded the corresponding products **7a–7b** in 81 and 80% yield, respectively. Similarly, the treatment of the nitrochalcones (**4a**, **4c**, **4d**, **4e**, and **4f**) with **6a** or **6b** provided the products **7c–7g** in the range of 68–78% yield.

**Table 4:** Formation of carbazoles **7a–7g** from **1a** or the 2-nitrochalcones and **6a** or **6b**^*a*^

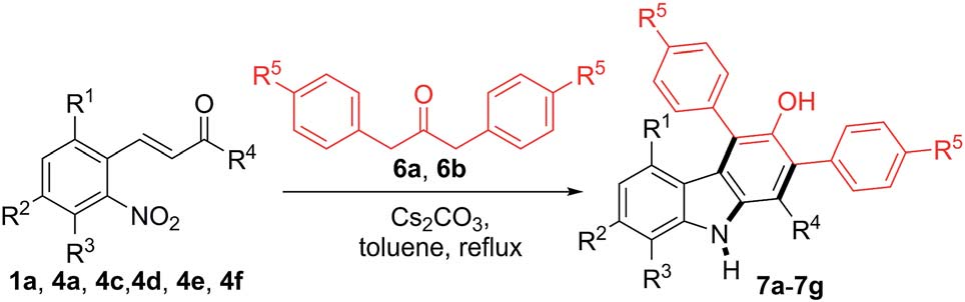
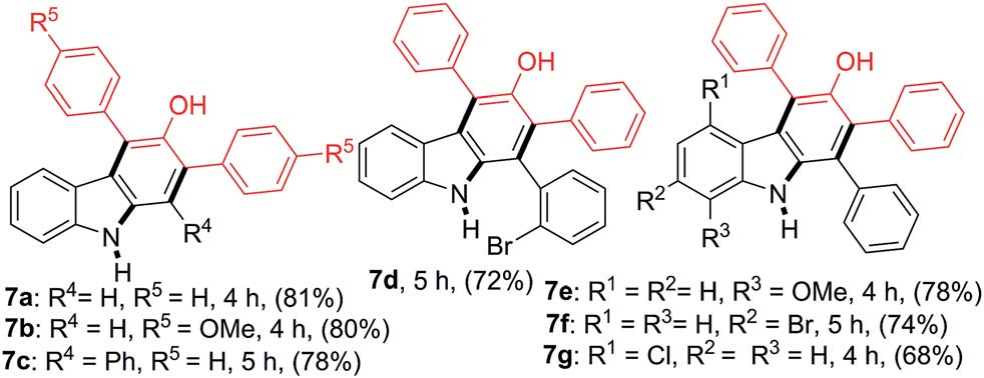

^*a*^Reactions were performed on a 1.0 mmol scale according to the standard conditions described in Table 1.

Having confirmed the general applicability of the reaction by using 2-nitrocinnamaldehyde and the 2-nitrochalcones as starting materials, the possibility of using the 2-nitrochalcones bearing a heteroatom was examined, which would lead to the formation of carbazole derivatives with extended structural space. To our delight, the reactions of **8a** or **8b** with β-ketoesters **2b**, **2d**, and **2e** provided the expected products **9a–9f** in the range of 71–75% yield (Table 5).

**Table 5:** Formation of carbazoles **9a–9f** from various 2-nitrochalcones (**8a** and **8b**) and β-ketoesters (**2b**, **2d** and **2e**)^*a*^

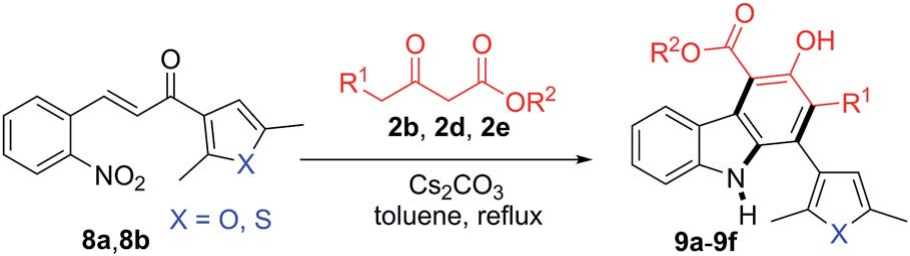
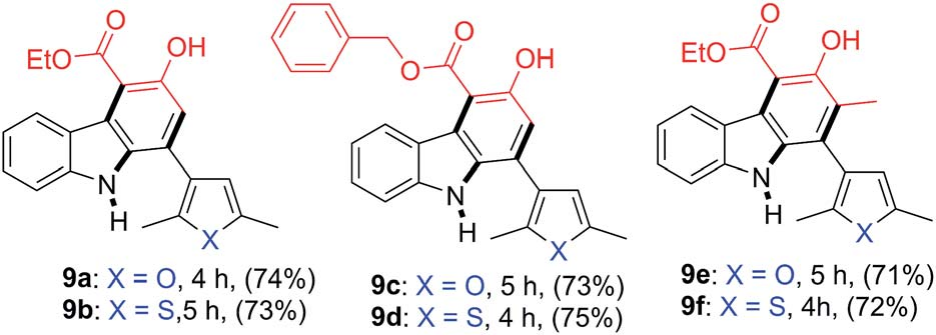

^*a*^Reactions were performed on a 1.0 mmol scale according to the standard conditions described in Table 1.

**Scheme 1:**
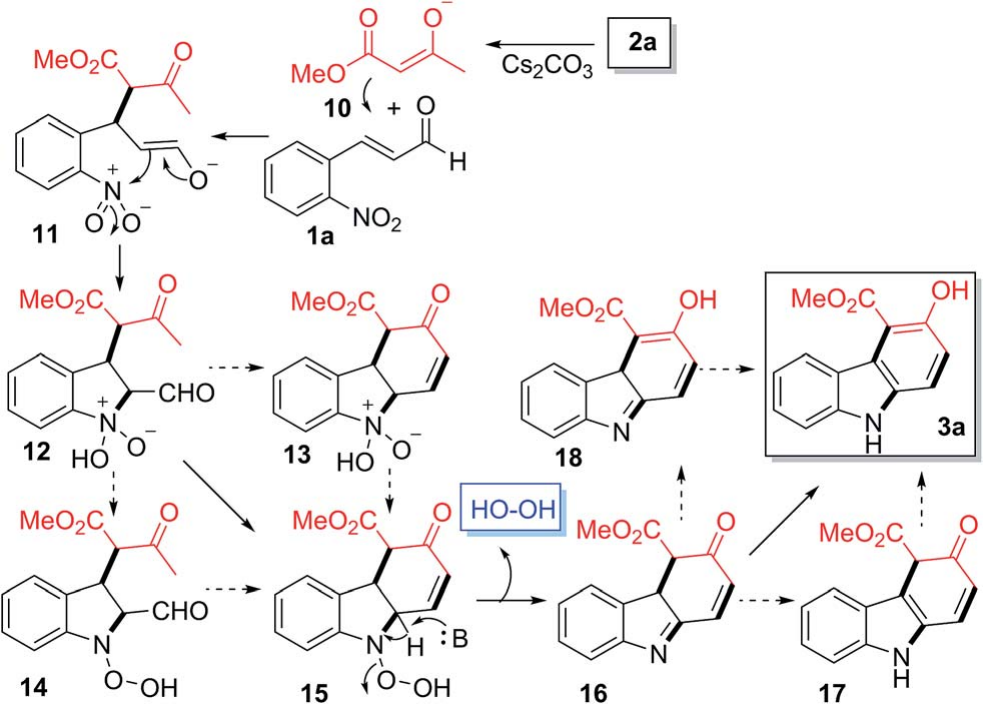
Proposed mechanism for the formation of **3a**.

We propose that the formation of the observed carbazole products may involve a mechanism shown in Scheme 1. In a basic medium, enolate **10** derived from **2a** undergoes Michael addition onto **1a** to give the new enolate intermediate **11**, which subsequently reacts with the nitro group to form the bicyclic intermediate **12**.^27^ The reorganization of the O–N–OH moiety in **12** to N–O–OH would generate **15***via***13** or **14**. The base-induced elimination of the hydrogen peroxide from **15** would generate **16**, which would then undergo sequential double tautomerization *via***17** or **18** to generate the observed product **3a**.

**Scheme 2:**
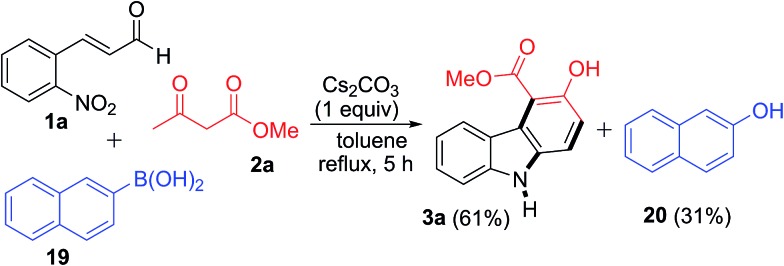
Control experiment to detect H_2_O_2_ in the reaction pathway.

To obtain evidence for the formation of H_2_O_2_ during the reaction sequence, a control experiment was carried out with added aryl boronic acid (Scheme 2). To our delight, this reaction involving **1a**, **2a** and 2-naphthyl boronic acid **19** under the standard reaction conditions provided product **3a** (61%) together with 2-naphthol **20** in 31% yield. The formation of 2-naphthol **20** implies the existence of *in situ* generated H_2_O_2_ in the reaction, although other mechanistic possibilities cannot be excluded.^28^

**Scheme 3:**
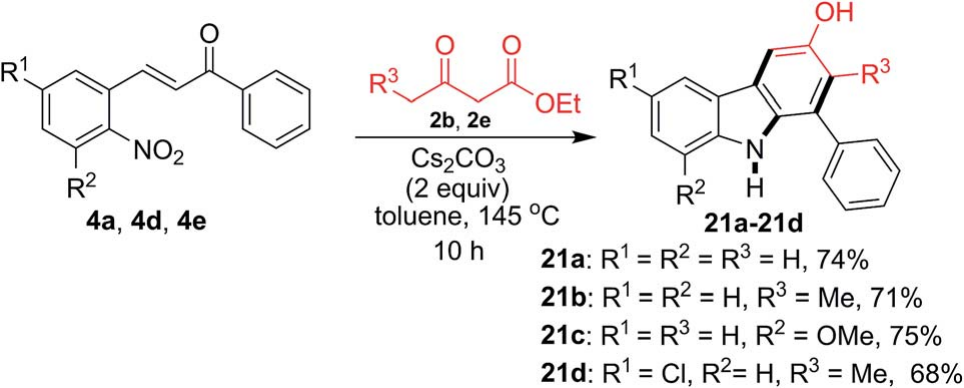
Formation of the decarboethoxylated carbazoles **21a–21d** from various 2-nitrochalcones and β-ketoesters.

Next, we broaden the carbazole structures to those that do not carry a carboethoxy group at the 4-position (Scheme 3). By carrying out the reaction at a higher temperature (145 °C) for a prolonged time using 2 equivalents of Cs_2_CO_3_ for decarboethoxylation, carbazoles **21a–21d** were obtained in 68–75% yield.

**Scheme 4:**
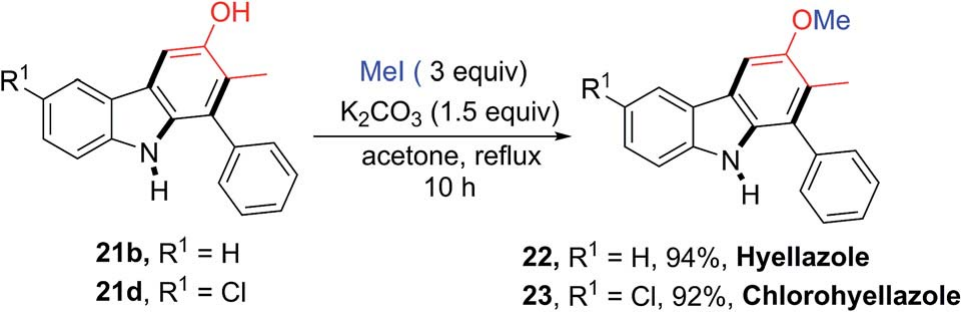
Synthesis of naturally occurring hyellazole (**22**) and chlorohyellazole (**23**).

The utility of this new protocol was demonstrated by the conversion of **21b** and **21d** to biologically active natural products (Scheme 4). Upon treating **21b** and **21d** with iodomethane in refluxing acetone in the presence of K_2_CO_3_, hyellazole (**22**) and chlorohyellazole (**23**) were obtained in 94% and 92% yields, respectively. Our concise synthesis of hyellazole and chlorohyellazole was achieved in two steps from commercially available starting materials in 67% and 63% overall yields, respectively. This protocol has several advantages such as higher yields, lower cost, fewer steps, transition metal-free, and environmentally benignity.^29^,^30^ The identity of these two natural products was confirmed by the comparison of their spectroscopic data with those previously reported.^29^,^30^

## Conclusions

A highly efficient, transition-metal-free, modular and operationally simple tandem annulation process was developed for the synthesis of diverse carbazole derivatives starting from readily available 2-nitrocinnamaldehydes or 2-nitrochalcones and β-ketoesters or 1,3-diaryl-2-propanones. This synthetic approach for the rapid construction of various functionalized carbazoles involves the intramolecular addition of an enolate to a nitro group and a unique *in situ* N–O bond cleavage under non-reductive conditions. As an application of this new synthetic methodology, a concise synthesis of naturally occurring bioactive hyellazole and chlorohyellazole has been realized in two steps.

## Supplementary Material

Supplementary information

Crystal structure data
