# Supplementary material for: Construction of highly functionalized carbazoles via condensation of an enolate to a nitro group
Source: Chem Sci. 2015 Sep 16;6(12):7028–33. doi: 10.1039/c5sc02407b (PMC5947529; doi:10.1039/c5sc02407b)
Supplement: Supplementary file 1 [file SC-006-C5SC02407B-s001.pdf]

Electronic Supplementary Material (ESI) for Chemical Science.
This journal is © The Royal Society of Chemistry 2015

SUPPORTING INFORMATION

Construction of highly functionalized carbazoles via condensation of an enolate to a nitro group

Tej Narayan Poudel and Yong Rok Lee *

School of Chemical Engineering, Yeungnam University, Gyeongsan 712-749, Republic of Korea

Email: yrlee@yu.ac.kr, Phone: +82-53-810-2529; Fax: +82-53-810-4631

TABLE OF CONTENTS

General experimental information **2**

General experimental procedures for compounds **3-9** **2**

Spectroscopic data of compounds **3-9** **2-10**

General experimental procedures control experiment **11**

General experimental procedures for compounds **21a-21d** **11**

Spectroscopic data of all compounds **21a-21d** **11-12**

General experimental procedures for **22** and **23** **12**

Spectroscopic data of compounds **22-23** **12**

^{1}H NMR and ^{13}C NMR spectra of compounds **3-9** **14-46**

^{1}H NMR and ^{13}C NMR spectra of compounds **20-23** **47-53**

X-ray structure and data of compound **7a** **54-71**

General experimental information

All experiments were carried out under open air without inert gases protection. 2-Nitrochalcones, 2-nitrocinnamaldehyde, 1,3-diphenyl-2-propanone and Ketoesters were purchased from Sigma-Aldrich or prepared by reported methods. Merck precoated silica gel plates (Art. 5554) with a fluorescent indicator were used for analytical TLC. Flash column chromatography was performed using silica gel 9385 (Merck). Melting points were determined with micro-cover glasses on a Fisher-Johns apparatus and are uncorrected. ^{1}H NMR spectra were recorded on a Varian-VNS (300 or 600 MHz) spectrometer in $CDCl_3$ using 7.24 ppm as the solvent chemical shift. ^{13}C NMR spectra were recorded on a Varian-VNS (75 or 150 MHz) spectrometer in $CDCl_3$ using 77.0 ppm as the solvent chemical shift. IR spectra were recorded on a JASCO FTIR 5300 spectrophotometer. High resolution mass (HRMS) were obtained with a JEOL JMS-700 spectrometer at the Korea Basic Science Institute.

General procedure for the synthesis carbazole derivatives (3-9)

A general procedure for the base catalyzed synthesis of carbazoles **3-9** is as follows: An oven dried two-neck round bottom flask was charged with ketoesters (1.0 mmol) or ketone (1.0 mmol) and 1.0 mmol of 2-nitrocinnamaldehyde or 2-nitrochalcone in 5 mL toluene and Cs_2CO_3 (1 equiv.) was added. Then, the flask was fitted with condenser. Each reaction mixture was refluxed 3-5 hours in open air without using nitrogen balloon until the completion of the reaction as indicated by TLC. Then solvent was evaporated in rotary evaporator under reduced pressure to obtain the residue. The residue was purified by flash column chromatography on silica gel to isolate the pure product. Characterization data for all compounds **3-9** are as follows:

Spectroscopic data of compounds 3-9

Methyl 3-hydroxy-9*H*-carbazole-4-carboxylate (3a). The title compound was prepared according to the general procedure. The product was obtained as a solid, mp 141-143 °C. Yield: 81% (195 mg). ^{1}H NMR (300 MHz, $CDCl_3$) δ 11.12 (1H, s), 8.43 (1H, d, J = 8.4 Hz), 8.17 (1H, s) , 7.52 (1H, d, J = 8.7 Hz), 7.41-7.40 (2H, m), 7.22-7.16 (1H, m), 7.09 (1H, d, J = 8.7 Hz), 4.16 (3H, s); ^{13}C NMR (75 MHz, $CDCl_3$) δ 171.6, 157.7, 140.6, 133.6, 126.2, 124.9, 122.2, 119.9, 119.2, 118.6, 116.5, 110.8, 105.7, 51.9; IR (KBr) 3391, 1618, 1340, 1274, 758, 540 cm^{-1}; HRMS *m/z* (M^+) calcd for $C_{14}H_{11}NO_3$: 241.0739. Found: 241.0738.

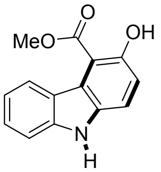

Ethyl 3-hydroxy-9*H*-carbazole-4-carboxylate (3b). The title compound was prepared according to the general procedure. The product was obtained as a solid, mp 112-114 °C. Yield: 79% (201 mg). ^{1}H NMR (300 MHz, $CDCl_3$) δ 11.19 (1H, s), 8.56 (1H, d, J = 8.4 Hz), 8.15 (1H, s), 7.47 (1H, d, J = 8.7 Hz), 7.44-7.36 (2H, m), 7.21-7.16 (1H, m), 7.09 (1H, d, J = 8.7 Hz), 4.67 (2H, q, J = 6.9 Hz), 1.56 (3H, t, J = 6.9 Hz); ^{13}C NMR (75 MHz, $CDCl_3$) δ 171.2, 157.6, 140.5, 133.6, 126.1, 125.3, 122.2, 119.9, 118.8, 118.4, 116.5, 110.8, 106.2, 61.7, 14.3; IR (KBr) 3399, 1648, 1311, 1083, 750, 628 cm^{-1}; HRMS *m/z* (M^+) calcd for $C_{15}H_{13}NO_3$: 255.0895. Found: 255.0897.

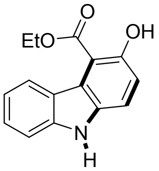

Allyl 3-hydroxy-9*H*-carbazole-4-carboxylate (3c). The title compound was prepared according to the general procedure. The product was obtained as a solid, mp 114-116 °C. Yield: 82% (218 mg).^{1}H NMR (300 MHz, $CDCl_3$) δ 11.14 (1H, s), 8.53 (1H, d, J = 8.4 Hz), 8.15 (1H, s), 7.46-7.33 (3H, m), 7.14 (1H, t, J = 7.8 Hz), 7.08 (1H, d, J = 8.7 Hz), 6.24-6.11 (1H, m), 5.50 (1H, d, J = 17.4 Hz), 5.38 (1H, d, J = 10.2 Hz), 5.08 (2H, d, J = 6.0 Hz); ^{13}C NMR (75 MHz, $CDCl_3$) δ 170.8, 157.6, 140.5, 133.6, 131.3, 126.1, 125.4, 122.1, 120.0, 119.9, 118.9, 118.6, 116.4, 110.7, 105.8, 66.3; IR (KBr) 3386, 3012, 1666, 1435, 1276, 760, 526 cm^{-1}; HRMS *m/z* (M^+) calcd for $C_{16}H_{13}NO_3$: 267.0895 Found: 267.0891.

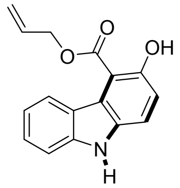

Benzyl 3-hydroxy-9*H*-carbazole-4-carboxylate (3d). The title compound was prepared according to the general procedure. The product was obtained as a solid, mp 170-172 °C. Yield: 77% (244 mg). ^{1}H NMR (300 MHz, $CDCl_3$) δ 11.17 (1H, s), 8.36 (1H, d, *J* = 8.4 Hz), 8.10 (1H, s), 7.45-7.48 (3H, m), 7.42-7.33 (5H, m), 7.09 (1H, d, *J* = 9.0 Hz), 6.94-6.88 (1H, m), 5.62 (2H, m); ^{13}C NMR (75 MHz, $CDCl_3$) δ 171.0, 157.9, 140.5, 134.7, 133.6, 129.1, 128.7, 128.7, 126.1, 125.6, 122.1, 120.0, 118.8, 118.6, 116.5, 110.6, 105.9, 67.5; IR (KBr) 3397, 2985, 1686, 1251, 970, 534 cm^{-1}; HRMS *m/z* (M^+) calcd for $C_{20}H_{15}NO_3$: 317.1052. Found: 317.1050.

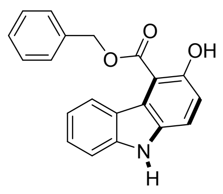

Ethyl 3-hydroxy-2-methyl-9*H*-carbazole-4-carboxylate (3e). The title compound was prepared according to the general procedure. The product was obtained as a solid, mp 140-142 °C. Yield: 78% (209 mg). ^{1}H NMR (600 MHz, $CDCl_3$) δ 11.54 (1H, s), 8.51 (1H, d, *J* = 8.4 Hz), 7.90 (1H, s), 7.38 (1H, t, *J* = 7.2 Hz), 7.32 (1H, d, *J* = 8.4 Hz), 7.26 (1H, s), 7.18 (1H, t, *J* = 7.2 Hz), 4.64 (2H, q, *J* = 7.2 Hz), 2.38 (3H, s), 1.54 (3H, t, *J* = 7.8 Hz); ^{13}C NMR (150 MHz, $CDCl_3$) δ 171.7, 156.5, 140.1, 133.2, 125.8, 125.5, 124.9, 122.4, 118.9, 118.7, 117.8, 110.6, 105.4, 61.7, 16.9, 14.3; IR (KBr) 3359, 2972, 1632, 1435, 1227, 1026, 737, 502 cm^{-1}; HRMS *m/z* (M^+) calcd for $C_{16}H_{15}NO_3$: 269.1052. Found: 269.1053.

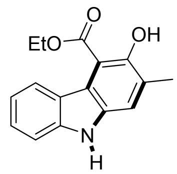

Ethyl 2-ethyl-3-hydroxy-9*H*-carbazole-4-carboxylate (3f). The title compound was prepared according to the general procedure. The product was obtained as a solid, mp 154-156 °C. Yield: 75% (212 mg). ^{1}H NMR (300 MHz, $CDCl_3$ + DMSO-d_6) δ 11.46 (1H, s), 8.98 (1H, s), 8.47 (1H, d, *J* = 8.4 Hz), 7.35-7.33 (3H, m), 7.13-7.09 (1H, m), 4.62 (2H, q, *J* = 7.2 Hz), 2.77 (2H, q, *J* = 7.2 Hz), 1.51 (3H, t, *J* = 7.2 Hz), 1.25 (3H, t, J = 7.2 Hz); ^{13}C NMR (75 MHz, $CDCl_3$ + DMSO-d_6) δ 171.7, 155.9, 140.3, 133.6, 131.4, 125.1, 124.7, 122.2, 121.1, 118.2, 117.4, 110.7, 105.2, 61.4, 23.5, 14.2, 13.6; IR (KBr) 3412, 1658, 1320, 1081, 771, 625 cm^{-1}; HRMS *m/z* (M^+) calcd for $C_{17}H_{17}NO_3$: 283.1208. Found: 283.1208.

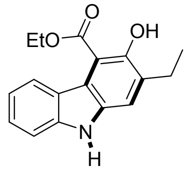

Methyl 2-butyl-3-hydroxy-9*H*-carbazole-4-carboxylate (3g). The title compound was prepared according to the general procedure. The product was obtained as a solid, mp 153-155 °C. Yield: 74% (219 mg). ^{1}H NMR (600 MHz, $CDCl_3$) δ 11.48 (1H, s), 8.38 (1H, d, *J* = 8.4 Hz), 8.00 (1H, s), 7.38-7.37 (3H, m), 7.19-7.17(1H, m), 4.15 (3H, s), 2.78 (2H, t, *J* = 7.2 Hz), 1.69-1.64 (2H, m), 1.44-1.38 (2H, m), 0.95 (3H, t, *J* = 7.2 Hz); ^{13}C NMR (150 MHz, $CDCl_3$) δ 172.2, 156.4, 140.2, 133.3, 130.5, 125.6, 124.6, 122.5, 119.0, 118.3, 117.8, 110.7, 105.1, 51.9, 31.6, 30.4, 22.6, 14.0; IR (KBr) 3434, 3054, 1663, 1436, 1276, 970, 760, 516 cm^{-1}; HRMS *m/z* (M^+) calcd for $C_{18}H_{19}NO_3$: 297.1365. Found: 297.1368.

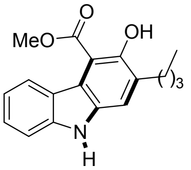

Methyl 3-hydroxy-2-octyl-9*H*-carbazole-4-carboxylate (3h). The title compound was prepared according to the general procedure. The product was obtained as a solid, mp 133-135 °C. Yield:

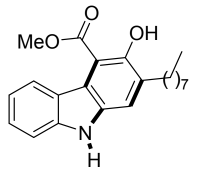

73% (257 mg). ^{1}H NMR (300 MHz, CDCl$_3$) δ 11.46 (1H, s), 8.38 (1H, d, J = 8.4 Hz), 8.01 (1H, s), 7.38-7.37 (3H, m), 7.20-7.15(1H, m), 4.15 (3H, s), 2.77 (2H, t, J = 7.8 Hz), 1.70-1.58 (2H, m), 1.34-1.26 (10H, m), 0.87 (3H, t, J = 7.2 Hz) ^{13}C NMR (75 MHz, CDCl$_3$) δ 172.1, 156.4, 140.2, 133.3, 130.6, 125.5, 124.5, 122.5, 119.0, 118.3, 117.9, 110.7, 105.1, 51.8, 31.8, 30.6, 29.6, 29.53, 29.51, 29.29, 22.6, 14.0; IR (KBr) 3361, 2926, 1657, 1436, 801, 650 cm^{-1}; HRMS *m/z* (M$^+$) calcd for $C_{22}H_{27}NO_3$: 353.1991. Found: 353.1988.

Methyl 3-hydroxy-2-phenyl-*9H*-carbazole-4-carboxylate (3i). The title compound was prepared according to the general procedure. The product was obtained as a solid, mp 149-151

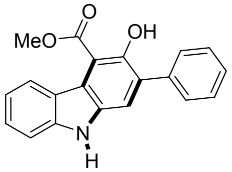

°C. Yield: 77% (244 mg). ^{1}H NMR (600 MHz, CDCl$_3$) δ 11.53 (1H, s), 9.78 (1H, s), 8.34 (1H, d, J = 8.4 Hz), 7.59-7.57 (3H, m), 7.40-7.37 (3H, m), 7.35-7.32 (1H, m), 7.30-7.28 (1H, m), 7.11 (1H, t, J = 7.8 Hz), 4.12 (3H, s); ^{13}C NMR (150 MHz, CDCl$_3$) δ 172.0, 154.8, 141.0, 138.0, 133.8, 129.4, 128.8, 127.9, 127.0, 125.8, 124.6, 121.8, 119.4, 118.9, 118.6, 110.9, 105.4, 51.9; IR (KBr) 3408, 2997, 1689, 1105, 740 cm^{-1}; HRMS *m/z* (M$^+$) calcd for $C_{20}H_{15}NO_3$: 317.1052. Found: 317.1048.

Allyl 3-hydroxy-1-phenyl-9*H*-carbazole-4-carboxylate (5a). The title compound was prepared

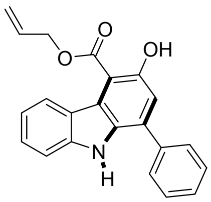

according to the general procedure. The product was obtained as a yellow liquid. Yield: 75% (257 mg). ^{1}H NMR (300 MHz, CDCl$_3$) δ 11.09 (1H, s), 8.56 (1H, d, J = 8.4 Hz), 8.31 (1H, s), 7.64 (2H, d, J = 7.5 Hz), 7.57-7.53 (2H, m), 7.48 (1H, d, J = 7.2 Hz), 7.43-7.37 (2H, m), 7.19-7.12 (2H, m), 6.26-6.13 (1H, m), 5.52 (1H, d, J = 17.1 Hz), 5.40 (1H, d, J = 10.5 Hz), 5.11 (2H, d, J = 6.0 Hz); ^{13}C NMR (75 MHz, CDCl$_3$) δ 170.8, 157.9, 140.5, 137.4, 132.9, 131.7, 131.4, 129.3, 128.8, 128.6, 128.3, 126.2, 125.5, 122.4, 120.0, 119.0, 116.1, 110.8, 105.0, 66.3; IR (neat) 3464, 1655, 1506, 1340, 1161, 724, 563 cm^{-1}; HRMS *m/z* (M$^+$) calcd for $C_{22}H_{17}NO_3$: 343.1208. Found: 343.1212.

Ethyl 3-hydroxy-2-methyl-1-phenyl-9*H*-carbazole-4-carboxylate (5b). The title compound was prepared according to the general procedure. The product was obtained as a solid, mp 145-

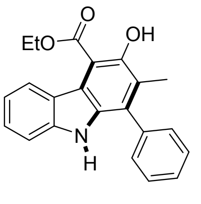

147 °C. Yield: 73% (252 mg). ^{1}H NMR (600 MHz, CDCl$_3$) δ 11.69 (1H, s), 8.54 (1H, d, J = 8.4 Hz), 7.76 (1H, s), 7.57-7.55 (2H, m), 7.50-7.48 (1H, m), 7.38 (2H, d, J = 7.8 Hz), 7.35-7.33 (1H, m), 7.29 (1H, d, J = 7.8 Hz), 7.17-7.14 (1H, m), 4.70 (2H, q, J = 7.2 Hz), 2.21 (3H, s), 1.58 (3H, t, J = 7.2 Hz); ^{13}C NMR (150 MHz, CDCl$_3$) δ 171.8, 156.9, 139.8, 136.5, 132.5, 132.1, 129.4, 129.1, 128.2, 125.5, 125.1, 123.5, 122.6, 118.7, 117.1, 110.6, 104.6, 61.7, 14.4, 13.6; IR

(KBr) 3433, 2929, 1711, 1680, 1452, 1262, 747, 557 cm^{-1}; HRMS *m/z* (M^+) calcd for $C_{22}H_{19}NO_3$: 345.1365. Found: 345.1363.

Methyl 3-hydroxy-1,2-diphenyl-9*H*-carbazole-4-carboxylate (5c). The title compound was prepared according to the general procedure. The product was obtained as a solid, mp 211-213 °C. Yield: 78% (306 mg). ^{1}H NMR (300 MHz, $CDCl_3$) δ 11.46 (1H, s), 8.43 (1H, d, J = 8.4 Hz), 7.95 (1H, s), 7.37-7.17 (13H, m), 4.17(3H, s); ^{13}C NMR (75 MHz, $CDCl_3$) δ 171.9, 155.7, 140.4, 136.1, 136.0, 132.5, 131.9, 131.1, 129.9, 128.6, 128.3, 127.7, 127.5, 126.7, 126.1, 124.9, 122.4, 119.2, 119.0, 110.8, 104.9, 52.0; IR (KBr) 3395, 3021, 142, 1509, 1157, 743, 635 cm^{-1}; HRMS *m/z* (M^+) calcd for $C_{26}H_{19}NO_3$: 393.1365. Found: 393.1365.

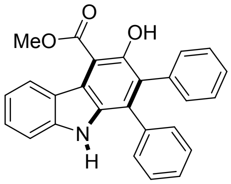

Ethyl 3-hydroxy-1-(*p*-tolyl)-9*H*-carbazole-4-carboxylate (5d). The title compound was prepared according to the general procedure. The product was obtained as a solid, mp 105-107 °C. Yield: 76% (262 mg). ^{1}H NMR (600 MHz, $CDCl_3$) δ 11.21 (1H, s), 8.58 (1H, d, J = 8.4 Hz), 8.32 (1H, s), 7.54 (2H, d, J = 8.1 Hz), 7.41-7.35 (4H, m), 7.18 (1H, t, J = 7.8 Hz), 7.10 (1H, s), 4.69 (2H, q, J = 7.2 Hz), 2.45 (3H, s), 1.58 (3H, t, J = 7.2 Hz); ^{13}C NMR (150 MHz, $CDCl_3$) δ 171.2, 157.9, 140.4, 138.5, 134.4, 132.8, 131.8, 130.0, 128.2, 126.1, 125.4, 122.5, 120.3, 118.9, 116.0, 110.8, 105.0, 61.7, 21.2, 14.4; IR (KBr) 3385, 2962, 1656, 1463, 750, 658 cm^{-1}; HRMS *m/z* (M^+) calcd for $C_{22}H_{19}NO_3$: 345.1365. Found: 345.1362.

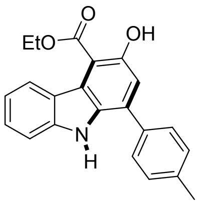

Ethyl 3-hydroxy-2-methyl-1-(*p*-tolyl)-9*H*-carbazole-4-carboxylate (5e). The title compound was prepared according to the general procedure. The product was obtained as a solid, mp 200-202 °C. Yield: 75% (269 mg). ^{1}H NMR (300 MHz, $CDCl_3$) δ 11.70 (1H, s), 8.55 (1H, d, J = 8.4 Hz), 7.81 (1H, s), 7.38-7.26 (6H, m), 7.19-7.13 (1H, m), 4.70 (2H, q, J = 7.2 Hz), 2.47 (3H, s), 2.22 (3H, s), 1.58 (3H, t, J = 7.2 Hz); ^{13}C NMR (75 MHz, $CDCl_3$) δ 171.8, 156.9, 139.8, 138.0, 133.5, 132.7, 132.2, 129.8, 129.2, 125.4, 125.0, 123.5, 122.6, 118.6, 117.0, 110.6, 104.4, 61.6, 21.3, 14.4, 13.5; IR (KBr) 3399, 3040, 1647, 1508, 1310, 1157, 749, 635 cm^{-1}; HRMS *m/z* (M^+) calcd for $C_{23}H_{21}NO_3$: 359.1521. Found: 359.1520.

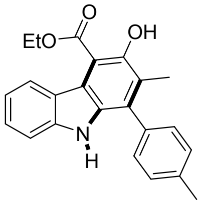

Benzyl 1-(2-bromophenyl)-3-hydroxy-9*H*-carbazole-4-carboxylate (5f). The title compound was prepared according to the general procedure. The product was obtained as a yellow liquid. Yield: 70% (329 mg). ^{1}H NMR (300 MHz, $CDCl_3$) δ 11.20 (1H, s), 8.41 (1H, d, J = 8.4 Hz), 7.88 (1H, s), 7.75 (1H, d, J = 7.8 Hz), 7.54-7.30 (10H, m), 7.04 (1H, s), 6.92-6.90 (1H, m), 5.65 (2H, s); ^{13}C NMR (75 MHz, $CDCl_3$) δ 170.9, 157.6, 140.4, 137.8, 134.7, 133.4, 131.9, 131.8, 131.2, 130.2, 129.2, 129.2, 128.7, 127.8, 126.2, 125.8, 122.9, 122.3,

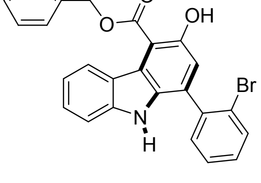

120.2, 118.9, 117.1, 110.7, 105.7, 67.5; IR (neat) 3390, 1635, 1509, 1212, 760, 638 cm^{-1}; HRMS *m/z* (M^{+}) calcd for $C_{26}H_{18}BrNO_3$: 471.0470. Found: 471.0474.

Ethyl 3-hydroxy-8-methoxy-1-phenyl-9*H*-carbazole-4-carboxylate (5g). The title compound was prepared according to the general procedure. The product was obtained as a solid, mp 118-120 °C. Yield: 75% (270 mg). ^{1}H NMR (600 MHz, $CDCl_3$) δ 11.22 (1H, s), 8.48 (1H, s), 8.14 (1H, d, J = 8.4 Hz), 7.66 (2H, d, J = 6.9 Hz), 7.56 (2H, t, J = 7.2 Hz), 7.48 (1H, t, J = 7.5 Hz), 7.12-7.09 (2H, m), 6.86 (1H, d, J = 7.8 Hz), 4.68 (2H, q, J = 7.2 Hz), 3.95 (3H, s), 1.57 (3H, t, J = 7.2 Hz); ^{13}C NMR (150 MHz, $CDCl_3$) δ 171.2, 157.9, 145.5, 137.4, 133.0, 131.5, 131.4, 129.3, 128.5, 128.3, 123.9, 120.8, 118.9, 117.7, 116.1, 105.4, 105.2, 61.7, 55.4, 14.4; IR (KBr) 3440, 3065, 1624, 1435, 1162, 1128, 756, 516 cm^{-1}; HRMS *m/z* (M^{+}) calcd for $C_{22}H_{19}NO_4$: 361.1314. Found: 361.1315.

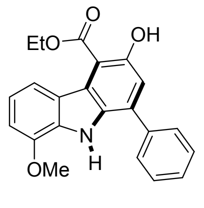

Methyl 3-hydroxy-8-methoxy-1,2-diphenyl-9*H*-carbazole-4-carboxylate (5h). The title compound was prepared according to the general procedure. The product was obtained as a solid, mp 238-240 °C. Yield: 78% (329 mg). ^{1}H NMR (300 MHz, $CDCl_3$) δ 11.56 (1H, s), 8.20 (1H, s), 8.07 (1H, d, J = 8.4 Hz), 7.36-7.14 (11H, m), 6.91 (1H, d, J = 7.5 Hz), 4.24 (3H, s), 3.98 (3H, s); ^{13}C NMR (150 MHz, $CDCl_3$) δ 171.9, 155.9, 145.5, 136.2, 136.1, 132.34, 132.31, 131.3, 131.1, 129.9, 128.5, 128.3, 127.7, 127.5, 126.7, 123.3, 119.3, 119.1, 117.3, 105.5, 104.8, 55.3, 52.0; IR (KBr) 3464, 3058, 1655, 1581, 1249, 722, 564 cm^{-1}; HRMS *m/z* (M^{+}) calcd for $C_{27}H_{21}NO_4$: 423.1471. Found: 423.1469.

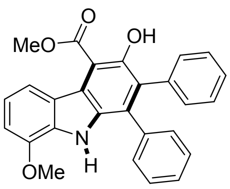

Methyl 7-bromo-3-hydroxy-1-phenyl-9*H*-carbazole-4-carboxylate (5i). The title compound was prepared according to the general procedure. The product was obtained as a solid, mp 177-179 °C. Yield: 70% (276 mg). ^{1}H NMR (600 MHz, $CDCl_3$) δ 11.17 (1H, s), 8.32 (1H, d, J = 8.4 Hz), 8.29 (1H, s), 7.62 (2H, d, J = 7.2 Hz), 7.56 (2H, t, J = 7.8 Hz), 7.53 (1H, s), 7.49 (1H, t, J = 7.8 Hz), 7.28 (1H, dd, J = 1.8, 9.6 Hz), 7.13 (1H, s), 4.17 (3H, s); ^{13}C NMR (150 MHz, $CDCl_3$) δ 171.2, 158.3, 141.1, 137.0, 133.0, 131.7, 129.4, 128.8, 128.3, 126.4, 122.6, 121.5, 120.0, 119.9, 116.7, 113.7, 104.6, 52.1; IR (KBr) 3384, 3054, 1653, 1426, 1226, 940, 770, 536 cm^{-1}; HRMS *m/z* (M^{+}) calcd for $C_{20}H_{14}BrNO_3$: 395.0157. Found: 395.0158.

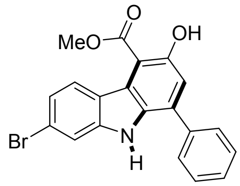

Ethyl 6-chloro-3-hydroxy-2-methyl-1-phenyl-9*H*-carbazole-4-carboxylate (5j). The title compound was prepared according to the general procedure. The product was obtained as a solid, mp 148-150 °C. Yield: 73% (276 mg). ^{1}H NMR (300 MHz, $CDCl_3$) δ 11.57 (1H, s), 8.37 (1H, d, J = 1.8 Hz), 7.53 (1H, s), 7.36-7.25 (3H, m), 7.16-7.13 (2H, m), 7.06 (1H, dd, J = 1.8, 8.7 Hz), 6.99 (1H, t, J = 7.8 Hz), 4.46 (2H, q,

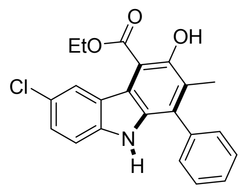

J = 7.2 Hz), 1.98 (3H, s), 1.41 (3H, t, J = 7.2 Hz); ^{13}C NMR (75 MHz, CDCl$_3$) δ 171.6, 157.5, 138.0, 136.3, 133.2, 132.3, 129.3, 129.2, 128.3, 125.5, 124.8, 124.6, 124.2, 123.6, 116.3, 111.4, 104.4, 62.0, 14.3, 13.6; IR (KBr) 3466, 2980, 1708, 1511, 1315, 903, 630 cm^{-1}; HRMS *m/z* (M$^+$) calcd for $C_{22}H_{18}ClNO_3$: 379.0975. Found: 379.0979.

Benzyl 6-chloro-3-hydroxy-1-phenyl-9*H*-carbazole-4-carboxylate (5k). The title compound was prepared according to the general procedure. The product was obtained as brown liquid.

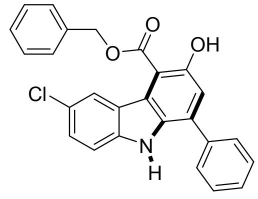

Yield: 71% (303 mg). ^{1}H NMR (300 MHz, CDCl$_3$) δ 11.24 (1H, s), 8.42 (1H, s), 8.29 (1H, s), 7.57-7.36 (10H, m), 7.25-7.17 (2H, m), 7.07 (1H, s), 5.59 (2H, s); ^{13}C NMR (75 MHz, CDCl$_3$) δ 170.6, 158.4, 138.5, 137.0, 134.4, 133.1, 132.2, 129.5, 129.3, 128.8, 128.7, 128.2, 127.9, 126.2, 125.1, 124.4, 123.3, 119.5, 116.9, 111.6, 104.8, 67.7; IR (neat) 3423, 2989, 1694, 1521, 1375, 703, 635 cm^{-1}; HRMS *m/z* (M$^+$) calcd for $C_{26}H_{18}ClNO_3$: 427.0975. Found: 427.0976.

2,4-Diphenyl-*9H*-carbazol-3-ol (7a). The title compound was prepared according to the general procedure.

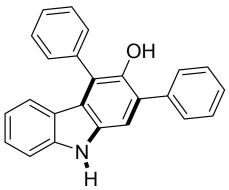

The product was obtained as a solid, mp 193-195 ^{o}C. Yield: 81% (271 mg). ^{1}H NMR (300 MHz, CDCl$_3$) δ 7.89 (1H, s), 7.67-7.59 (7H, m), 7.49-7.44 (2H, m), 7.39-7.30 (4H, m), 6.99-6.89 (2H, m), 4.97 (1H, m); ^{13}C NMR (150 MHz, CDCl$_3$) δ 143.4, 140.6, 138.7, 135.3, 134.1, 130.3, 129.6, 129.4, 128.4, 128.4, 127.6, 127.2, 125.6, 122.9, 121.9, 121.8, 121.6, 118.8, 111.3, 110.4; IR (KBr) 3394, 3091, 1649, 1518, 1320, 1187, 749, 635 cm^{-1}; HRMS *m/z* (M$^+$) calcd for $C_{24}H_{17}NO$: 335.1310. Found: 335.1309.

2,4-Bis(4-methoxyphenyl)-9*H*-carbazol-3-ol (7b). The title compound was prepared according

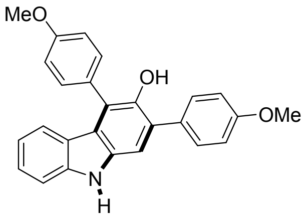

to the general procedure. The product was obtained as a solid, mp 155-157 ^{o}C. Yield: 80% (316 mg). ^{1}H NMR (300 MHz, CDCl$_3$ + DMSO-d_6) δ 9.25 (1H, s), 7.53 (2H, d, J = 8.7 Hz), 7.43 (2H, d, J = 8.7 Hz), 7.31-7.18 (3H, m), 7.07 (2H, d, J = 8.7 Hz), 6.98-6.90 (3H, m), 6.81 (1H, t, J = 7.8 Hz), 5.14 (1H, s), 3.86 (3H, s), 3.78 (3H, s); ^{13}C NMR (75 MHz, CDCl$_3$ + DMSO-d_6) δ 159.2, 158.4, 143.1, 140.6, 134.2, 131.3, 131.0, 130.4, 127.2, 126.9, 124.9, 122.6, 121.4, 121.1, 121.0, 117.9, 114.5, 113.5, 110.9, 110.3, 55.1, 55.0; IR (KBr) 3371, 2936, 1659, 1446, 1308, 801, 851, 540 cm^{-1}; HRMS *m/z* (M$^+$) calcd for $C_{26}H_{21}NO_3$: 395.1521. Found: 395.1519.

1,2,4-Triphenyl-9*H*-carbazol-3-ol (7c).The title compound was prepared according to the

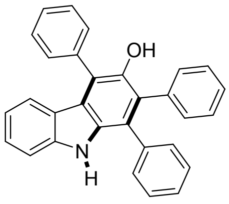

general procedure. The product was obtained as a solid, mp 275-277 ^{o}C. Yield: 78% (320 mg). ^{1}H NMR (300 MHz, CDCl$_3$) δ 8.17 (1H, s), 7.95-7.83 (5H, m), 7.59-7.50 (12H, m), 7.35 (1H, d, J = 8.4 Hz), 7.21-7.17 (1H, m), 5.18 (1H, s); ^{13}C NMR (75 MHz, CDCl$_3$) δ 143.8, 140.2, 137.0, 136.1,

135.7, 132.8, 131.3, 130.4, 130.3, 129.2, 128.5, 128.5, 128.2, 128.2, 127.1, 126.1, 125.6, 123.8, 123.1, 122.1, 121.1, 120.8, 118.7, 110.4; IR (KBr) 3440, 3066, 1612, 1391, 1128, 752 cm^{-1}; HRMS *m/z* (M^+) calcd for $C_{30}H_{21}NO$: 411.1623. Found: 411.1623.

1-(2-Bromophenyl)-2,4-diphenyl-9*H*-carbazol-3-ol (7d). The title compound was prepared according to the general procedure. The product was obtained as a solid, mp 213-215 ^{o}C. Yield: 72% (352 mg). ^{1}H NMR (300 MHz, $CDCl_3$) δ 7.92-7.88 (2H, m), 7.85-7.75 (5H, m), 7.57-7.55 (2H, m), 7.48-7.47 (3H, m), 7.44-7.40 (4H, m), 7.37-7.29 (2H, m), 7.14-7.08 (1H, m), 5.07 (1H, s); ^{13}C NMR (75 MHz, $CDCl_3$) δ 143.6, 140.4, 138.0, 135.9, 135.7, 132.8, 132.6, 130.7, 130.5, 129.21, 129.2, 129.1, 128.2, 128.1, 127.3, 127.2, 126.5, 125.6, 124.6, 123.2, 123.0, 122.1, 121.1, 121.1, 118.8, 110.5; IR (KBr) 3433, 3058, 1711, 1608, 1262, 747 cm^{-1}; HRMS *m/z* (M^+) calcd for $C_{30}H_{20}BrNO$: 489.0728. Found: 489.0726.

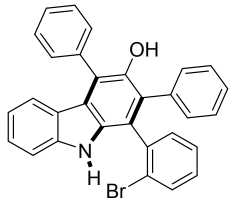

8-Methoxy-1,2,4-triphenyl-9*H*-carbazol-3-ol (7e). The title compound was prepared according to the general procedure. The product was obtained as a solid, mp 244-246 ^{o}C. Yield: 78% (343 mg). ^{1}H NMR (300 MHz, $CDCl_3$) δ 8.04 (1H, s), 7.68-7.51 (5H, m), 7.32-7.19 (10H, m), 6.86-6.76 (2H, m), 6.67 (1H, d, J = 7.2 Hz), 4.88 (1H, s), 3.91 (3H, s); ^{13}C NMR (75 MHz, $CDCl_3$) δ 155.9, 145.4, 143.8, 137.1, 136.2, 135.8, 132.7, 131.3, 130.7, 130.4, 130.3, 129.0, 128.5, 128.2, 128.1, 127.0, 126.3, 124.3, 124.1, 121.6, 120.8, 118.9, 114.7, 105.6, 55.3; IR (KBr) 3394, 3084, 1693, 1456, 1266, 870, 751, 517 cm^{-1}; HRMS *m/z* (M^+) calcd for $C_{31}H_{23}NO_2$: 441.1729. Found: 441.1729.

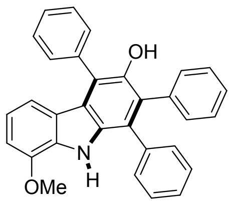

7-Bromo-1,2,4-triphenyl-9*H*-carbazol-3-ol (7f). The title compound was prepared according to the general procedure. The product was obtained as a solid, mp 252-254 ^{o}C. Yield: 74% (361 mg). ^{1}H NMR (300 MHz, $CDCl_3$) δ 7.98 (1H, s), 7.75-7.66 (5H, m), 7.54 (1H, s), 7.44-7.37 (10H, m), 7.11 (1H, d, J = 8.4 Hz), 7.00 (1H, d, J = 8.7 Hz), 5.00 (1H, s); ^{13}C NMR (75 MHz, $CDCl_3$) δ 144.2, 141.0, 136.8, 135.9, 135.5, 132.9, 131.2, 130.2, 130.2, 129.2, 129.1, 128.6, 128.3, 128.2, 127.3, 126.7, 124.1, 123.3, 122.2, 122.1, 120.8, 120.7, 119.2, 113.4; IR (KBr) 3359, 3064, 1673, 1446, 1296, 960, 740, 505 cm^{-1}; HRMS *m/z* (M^+) calcd for $C_{30}H_{20}BrNO$: 489.0728. Found: 489.0726.

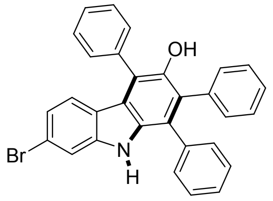

5-Chloro-1,2,4-triphenyl-9*H*-carbazol-3-ol (7g). The title compound was prepared according to the general procedure. The product was obtained as a solid, mp 240-242°C. Yield: 68% (302 mg). ^{1}H NMR (600 MHz, $CDCl_3$) δ 7.75-7.72 (2H, m), 7.68-7.64 (4H, m), 7.59 (1H, t, J = 7.8 Hz), 7.40 (2H, d, J = 7.2 Hz), 7.33-7.26 (4H, m), 7.26-7.24 (3H, m), 7.22-7.18 (1H, m), 7.13 (1H, d, J = 7.8 Hz), 6.96-6.94 (1H, m), 4.93 (1H, s); ^{13}C NMR (150 MHz, $CDCl_3$) δ

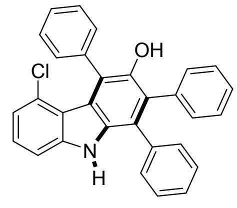

144.0, 138.5, 136.8, 135.9, 135.1, 133.5, 131.2, 130.2, 130.1, 129.3, 129.1, 128.6, 128.5, 128.2, 127.2, 127.0, 126.9, 125.7, 124.3, 124.1, 121.7, 120.4, 113.2, 111.3; IR (KBr) 3353, 3058, 1685, 1446, 1296, 960, 742, 509 cm^{-1}; HRMS *m/z* (M^+) calcd for $C_{30}H_{20}ClNO$: 445.1233. Found: 445.1235.

Ethyl 1-(2,5-dimethylfuran-3-yl)-3-hydroxy-9*H*-carbazole-4-carboxylate (9a). The title compound was prepared according to the general procedure. The product was obtained as a brown liquid. Yield: 74% (258 mg). ^1H NMR (600 MHz, $CDCl_3$) δ 11.24 (1H, s), 8.58 (1H, d, J = 9.0 Hz), 8.20 (1H, s), 7.40-7.39 (2H, m), 7.19-7.17 (1H, m), 6.99 (1H, s), 6.21 (1H, s), 4.68 (2H, q, J = 7.2 Hz), 2.36 (3H, s), 2.33 (3H, s), 1.57 (3H, t, J = 7.2 Hz); ^{13}C NMR (150 MHz, $CDCl_3$) δ 171.3, 157.8, 151.1, 147.9, 140.3, 132.4, 126.1, 125.7, 125.4, 122.6, 119.9, 118.9, 116.9, 116.1, 110.9, 106.8, 104.8, 61.7, 14.4, 13.5, 12.6; IR (neat) 3380, 2923, 1650, 1392, 1309, 1240, 740 cm^{-1}; HRMS *m/z* (M^+) calcd for $C_{21}H_{19}NO_4$: 349.1314. Found: 349.1314.

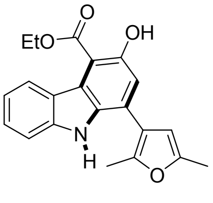

Ethyl 1-(2,5-dimethylthiophen-3-yl)-3-hydroxy-9*H*-carbazole-4-carboxylate (9b). The title compound was prepared according to the general procedure. The product was obtained as brown liquid. Yield: 73% (266 mg). ^1H NMR (600 MHz, $CDCl_3$) δ 11.22 (1H, s), 8.58 (1H, d, J = 8.4 Hz), 8.10 (1H, s), 7.40-7.39 (2H, m), 7.18-7.17 (1H, m), 6.99 (1H, s), 6.77 (1H, s), 4.68 (2H, q, J = 7.2 Hz), 2.50 (3H, s), 2.34 (3H, s), 1.57 (3H, t, J = 7.2 Hz); ^{13}C NMR (150 MHz, $CDCl_3$) δ 171.2, 157.6, 140.3, 137.5, 134.9, 133.1, 132.4, 128.0, 126.1, 126.1, 125.4, 122.5, 119.9, 118.9, 116.7, 110.8, 105.1, 61.7, 15.2, 14.4, 13.8; IR (neat) 3385, 2935, 1681, 1397, 209, 1140, 743 cm^{-1}; HRMS *m/z* (M^+) calcd for $C_{21}H_{19}NO_3S$: 365.1086. Found: 365.1085.

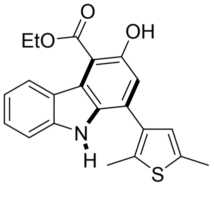

Benzyl 1-(2,5-dimethylfuran-3-yl)-3-hydroxy-9*H*-carbazole-4-carboxylate (9c). The title compound was prepared according to the general procedure. The product was obtained as a yellow liquid. Yield: 73% (300 mg). ^1H NMR (300 MHz, $CDCl_3$) δ 11.23 (1H, s), 8.39 (1H, d, J = 8.4 Hz), 8.16 (1H, s), 7.54-7.52 (2H, m), 7.40-7.29 (6H, m), 6.99 (1H, s), 6.20 (1H, s), 5.64 (2H, s), 2.35 (3H, s), 2.32 (3H, s); ^{13}C NMR (75 MHz, $CDCl_3$) δ 171.0, 158.0, 151.1, 147.9, 140.2, 134.9, 132.4, 129.1, 128.73, 128.70, 126.0, 125.9, 125.7, 122.5, 120.0, 118.8, 117.0, 116.0, 110.7, 106.8, 104.5, 67.3, 13.5, 12.5; IR (neat) 3395, 2963, 1667, 1389, 1307, 1262, 756 cm^{-1}; HRMS *m/z* (M^+) calcd for $C_{26}H_{21}NO_4$: 411.1471. Found: 411.1471.

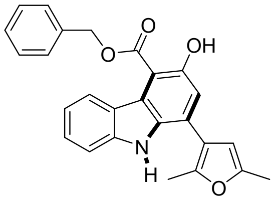

Benzyl 1-(2,5-dimethylthiophen-3-yl)-3-hydroxy-9*H*-carbazole-4-carboxylate (9d). The title compound was prepared according to the general procedure. The product was obtained as brown liquid. Yield: 75% (320 mg). ^1H NMR (300 MHz, $CDCl_3$) δ 11.21 (1H, s), 8.41 (1H, d, J = 8.4 Hz), 8.07 (1H, s), 7.55-7.53

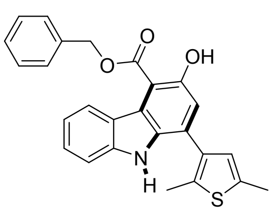

(2H, m), 7.41-7.32 (5H, m), 7.00 (1H, s), 6.94-6.90 (1H, m), 6.76 (1H, s), 5.65 (2H, s), 2.49 (3H, s), 2.33 (3H, s); ^{13}C NMR (150 MHz, $CDCl_3$) δ 170.9, 157.9, 140.3, 137.5, 134.96, 134.93, 133.2, 132.5, 129.1, 128.8, 128.7, 128.3, 126.2, 126.1, 125.8, 122.5, 120.1, 118.9, 116.8, 110.7, 104.9, 67.4, 15.2, 13.8; IR (neat) 3387, 2989, 1675, 1397, 209, 1140, 743 cm^{-1}; HRMS *m/z* (M^+) calcd for $C_{26}H_{21}NO_3S$: 427.1242. Found: 427.1239.

Ethyl 1-(2,5-dimethylfuran-3-yl)-3-hydroxy-2-methyl-9*H*-carbazole-4-carboxylate (9e). The title compound was prepared according to the general procedure. The product was obtained as brown liquid. Yield: 71% (257 mg). ^{1}H NMR (600 MHz, $CDCl_3$) δ 11.68 (1H, s), 8.53 (1H, d, J = 8.4 Hz), 7.95 (1H, s), 7.36-7.35 (2H, m), 7.16-7.14 (1H, m), 6.02 (1H, s), 4.68 (2H, q, J = 7.2 Hz), 2.37 (3H, s), 2.25 (3H, s), 2.10 (3H, s), 1.57 (3H, t, J = 7.2 Hz); ^{13}C NMR (150 MHz, $CDCl_3$) δ 171.8, 156.8, 151.0, 147.6, 139.6, 133.0, 125.4, 125.0, 124.6, 124.5, 122.7, 118.6, 116.6, 115.6, 110.7, 107.8, 104.4, 61.6, 14.4, 13.6, 13.6, 12.4; IR (neat) 3398, 2990, 1670, 1383, 1318, 1228, 747 cm^{-1}; HRMS *m/z* (M^+) calcd for $C_{22}H_{21}NO_4$: 363.1471. Found: 363.1467.

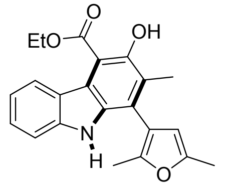

Ethyl 1-(2,5-dimethylthiophen-3-yl)-3-hydroxy-2-methyl-9*H*-carbazole-4-carboxylate (9f). The title compound was prepared according to the general procedure. The product was obtained as brown liquid. Yield: 72% (272 mg). ^{1}H NMR (600 MHz, $CDCl_3$) δ 11.69 (1H, s), 8.54 (1H, d, J = 8.4 Hz), 7.83 (1H, s), 7.35-7.34 (2H, m), 7.17-7.14 (1H, m), 6.58 (1H, s), 4.69 (2H, q, J = 7.2 Hz), 2.50 (3H, s), 2.20 (3H, s), 2.13 (3H, s), 1.57 (3H, t, J = 7.2 Hz); ^{13}C NMR (150 MHz, $CDCl_3$) δ 171.8, 156.7, 139.7, 137.4, 134.7, 132.9, 132.4, 127.3, 126.3, 125.4, 125.0, 124.6, 122.6, 118.6, 116.7, 110.7, 104.6, 61.7, 15.3, 14.4, 13.6, 13.4; IR (neat) 3394, 2963, 1690, 1381, 1330, 1250, 751 cm^{-1}; HRMS *m/z* (M^+) calcd for $C_{22}H_{21}NO_3S$: 379.1242. Found: 379.1244.

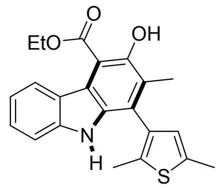

General procedure for control experiment:

An oven dried two-neck round bottom flask was charged with ketoesters **2a** (1.0 mmol) and 1.0 mmol of 2-nitrocinnamaldehyde (**1a)** and 1.0 mmol of naphthyl boronic acid (**19)** in 5 mL toluene and Cs_2CO_3 (1 equiv.) was added. Then the flask is fitted with condenser. The reaction mixture was refluxed for 5 hours. Then solvent was evaporated in rotary evaporator under reduced pressure to obtain the residue. The residue was purified by flash column chromatography on silica gel to isolate the pure products **3a** and 2-naphthol (**20)** in 61% (155 mg) and 31% (44 mg) yields respectively.

General procedure for the synthesis carbazole derivatives (21a-21d)

A general procedure for the base catalyzed synthesis of carbazoles **21a**-**21d** is as follows: An oven dried two-neck round bottom flask was charged with ketoesters (1.0 mmol) and 1.0 mmol of 2-nitrochalcone in 5 mL toluene and, Cs_2CO_3 (2 equiv.) was added. Then, the flask was fitted

with condenser. Each reaction mixture was heated at 145 ^{0}C for 10 hours in open air without using nitrogen balloons until the completion of the reaction as indicated by TLC. Then solvent was evaporated in rotary evaporator under reduced pressure to obtain the residue. The residue was purified by flash column chromatography on silica gel to isolate the pure product. Characterization data for all compounds **21a-21d** are as follows:

1-Phenyl-9*H*-carbazol-3-ol (21a). The title compound was prepared according to the general procedure. The product was obtained as a yellow liquid. Yield: 74% (191 mg). ^{1}H NMR (600 MHz, $CDCl_3$) δ 8.09 (1H, s), 7.99 (1H, d, J = 8.4 Hz), 7.65 (2H, d, J = 7.8 Hz), 7.53 (2H, t, J = 6.6 Hz), 7.48 (1H, d, J = 1.8 Hz), 7.42 (1H, t, J = 7.8 Hz), 7.39-7.34 (2H, m), 7.19-7.17 (1H, m), 7.00 (1H, d, J = 1.8 Hz), 4.73 (1H, brs); ^{13}C NMR (150 MHz, $CDCl_3$) δ 149.6, 140.3, 138.5, 129.2, 128.3, 128.2, 127.7, 126.0, 125.7, 124.4, 123.2, 120.5, 119.1, 114.6, 110.7, 104.9; IR (neat) 3340, 3022, 1421, 1151, 748, 650 cm^{-1}; HRMS *m/z* (M^+) calcd for $C_{18}H_{13}NO$: 259.0997. Found: 259.0999.

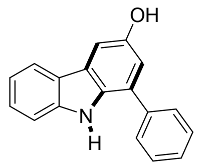

2-Methyl-1-phenyl-9*H*-carbazol-3-ol (21b). The title compound was prepared according to the general procedure. The product was obtained as a solid, mp 177-179 ^{o}C. Yield: 71% (193 mg). ^{1}H NMR (600 MHz, $CDCl_3$) δ 7.95 (1H, d, J = 7.8 Hz), 7.58 (1H, brs), 7.53 (2H, t, J = 7.8 Hz), 7.46-7.44 (2H, m), 7.41 (2H, d, J = 6.6 Hz), 7.32 (1H, t, J = 7.8 Hz), 7.26 (1H, d, J = 7.2 Hz), 7.15 (1H, t, J = 7.8 Hz), 4.40-4.00 (1H, brs), 2.23 (3H, s).; ^{13}C NMR (150 MHz, $CDCl_3$) δ 148.1, 139.7, 137.4, 133.6, 129.8, 129.1, 129.0, 127.6, 125.3, 123.2, 121.2, 120.8, 120.1, 118.8, 110.5, 104.4, 13.4; IR (KBr) 3433, 2950, 1640, 1452, 1267, 748, 590 cm^{-1}; HRMS *m/z* (M^+) calcd for $C_{19}H_{15}NO$: 273.1154. Found: 273.1151.

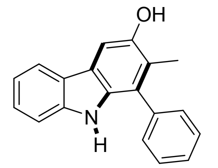

8-Methoxy-1-phenyl-9*H*-carbazol-3-ol (21c). The title compound was prepared according to the general procedure. The product was obtained as a yellow liquid. Yield: 75% (216 mg). ^{1}H NMR (600 MHz, $CDCl_3$) δ 8.30 (1H, s), 7.65 (2H, d, J = 7.8 Hz), 7.59 (1H, d, J = 7.8 Hz), 7.51 (2H, t, J = 7.8 Hz), 7.45 (1H, d, J = 1.8 Hz), 7.41 (1H, t, J = 7.8 Hz), 7.11 (1H, t, J = 7.8 Hz), 6.99 (1H, d, J = 1.8 Hz), 6.86 (1H, d, J = 7.8 Hz), 4.97 (1H, brs), 3.95 (3H, s); ^{13}C NMR (150 MHz, $CDCl_3$) δ 149.5, 145.6, 138.5, 132.0, 130.6, 129.1, 128.3, 127.6, 126.0, 124.7, 124.1, 119.4, 114.6, 112.9, 105.9, 104.9, 55.3; IR (neat) 3320, 3010, 1508, 1320, 1154, 741 cm^{-1}; HRMS *m/z* (M^+) calcd for $C_{19}H_{15}NO_2$: 289.1103. Found: 289.1101.

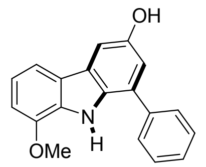

6-Chloro-2-methyl-1-phenyl-9*H*-carbazol-3-ol (21d). The title compound was prepared according to the general procedure. The product was obtained as a solid, mp 187-188 ^{o}C. Yield: 68% (208 mg). ^{1}H NMR (600 MHz, $CDCl_3$) δ 7.90 (1H, s), 7.57 (1H, brs), 7.53 (2H, t, J = 7.8 Hz), 7.45 (1H, d, J = 7.8 Hz), 7.40-7.39 (3H, m), 7.25 (1H, d, J = 8.4 Hz), 7.17 (1H, d, J = 8.4 Hz), 4.72 (1H,

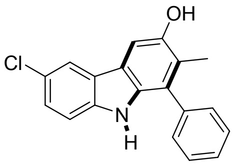

brs), 2.22 (3H, s); ^{13}C NMR (75 MHz, $CDCl_3$) δ 147.0, 138.0, 136.8, 134.2, 130.1, 129.6, 129.1, 128.0, 127.01, 125.5, 124.2, 123.2, 120.6, 117.9, 111.3, 109.8, 14.0; IR (KBr) 3345, 2950, 1530, 1463, 758 cm^{-1}; HRMS *m/z* (M^+) calcd for $C_{19}H_{14}ClNO$: 307.0764. Found: 307.0764.

General procedure for the synthesis Hyellazole and Chlorohyllazole (22-23)

To a mixture of compound **21b or 21d** (0.18 mmol) and K_2CO_3 (50 mg, 0.36 mmol) in acetone (6 mL) was added MeI (23 μL, 0.37 mmol) under nitrogen atmosphere and the reaction was refluxed for 16 h. The reaction mixture was concentrated *in vacuo* and the obtained residue was subjected to silica gel (60–120 mesh) column chromatograpy to obtain compounds hyellazole **22** and chlorohyellazole **23** in pure form.

3-Methoxy-2-methyl-1-phenyl-9*H*-carbazole (22). (Hyellazole) The title compound was prepared according to the general procedure. The product was obtained as a solid, mp 132-134 ^{0}C: 94% (46 mg). ^{1}H NMR (600 MHz, $CDCl_3$) δ 8.02 (1H, d, J = 7.8 Hz), 7.60 (1H, brs), 7.54-7.51 (3H, m), 7.45 (1H, d, J = 7.2 Hz), 7.42 (2H, d, J = 7.8 Hz), 7.32 (1H, t, J = 7.2 Hz), 7.28 (1H, d, J = 8.4 Hz), 7.18 (1H, t, J = 8.4 Hz), 3.99 (3H, s), 2.21 (3H, s); ^{13}C NMR (150 MHz, $CDCl_3$) δ 152.7, 139.4, 137.5, 133.2, 129.8, 128.9, 127.5, 125.5, 125.0, 123.8, 123.6, 120.3, 119.9, 118.8, 110.6, 100.3, 56.2, 13.7; IR (KBr) 3400, 3360, 3065, 2945, 1550, 1495, 1380, 720 cm^{-1}; HRMS *m/z* (M^+) calcd for $C_{20}H_{17}NO$: 287.1310. Found: 287.1308.

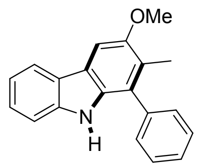

6-Chloro-3-methoxy-2-methyl-1-phenyl-9*H*-carbazole (23). (Chlorohyellazole) The title compound was prepared according to the general procedure. The product was obtained as a solid, mp 163-164 ^{0}C: 92% (50 mg). ^{1}H NMR (300 MHz, $CDCl_3$) δ 7.94 (1H, s), 7.59 (1H, brs), 7.51-7.47 (2H, m), 7.43-7.34 (4H, m), 7.21-7.13 (2H, m), 3.93 (3H, s), 2.15 (3H, s); ^{13}C NMR (75 MHz, $CDCl_3$) δ 152.8, 137.6, 137.1, 133.8, 129.8, 129.0, 127.7, 125.7, 125.0, 124.8, 124.7, 124.2, 119.6, 119.4, 111.5, 99.9, 56.0, 13.8; IR (KBr) 3355, 3058, 2950, 1545, 1485, 1370, 725 cm^{-1}; HRMS *m/z* (M^+) calcd for $C_{20}H_{16}ClNO$: 321.0920. Found: 321.0923.

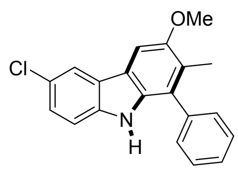

^{1}H NMR and ^{13}C NMR Spectra of all compounds

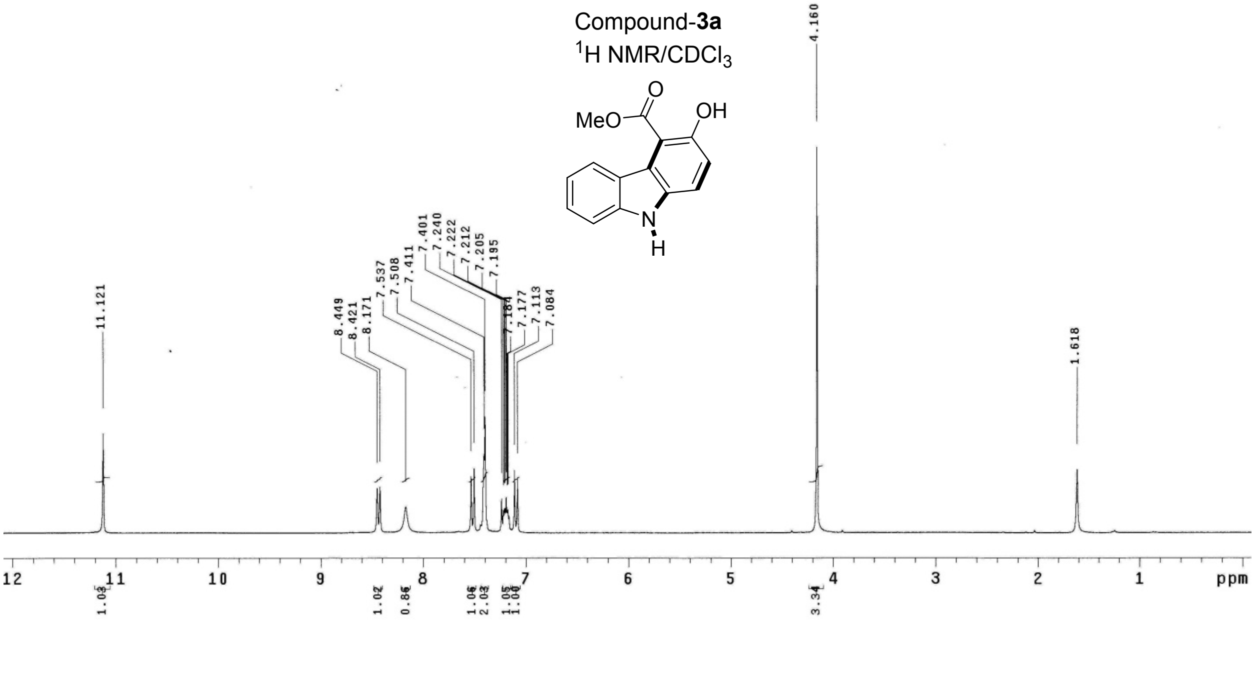

Compound-**3a**
^{13}C NMR/$CDCl_3$

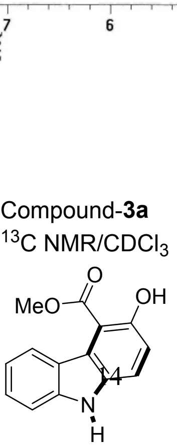

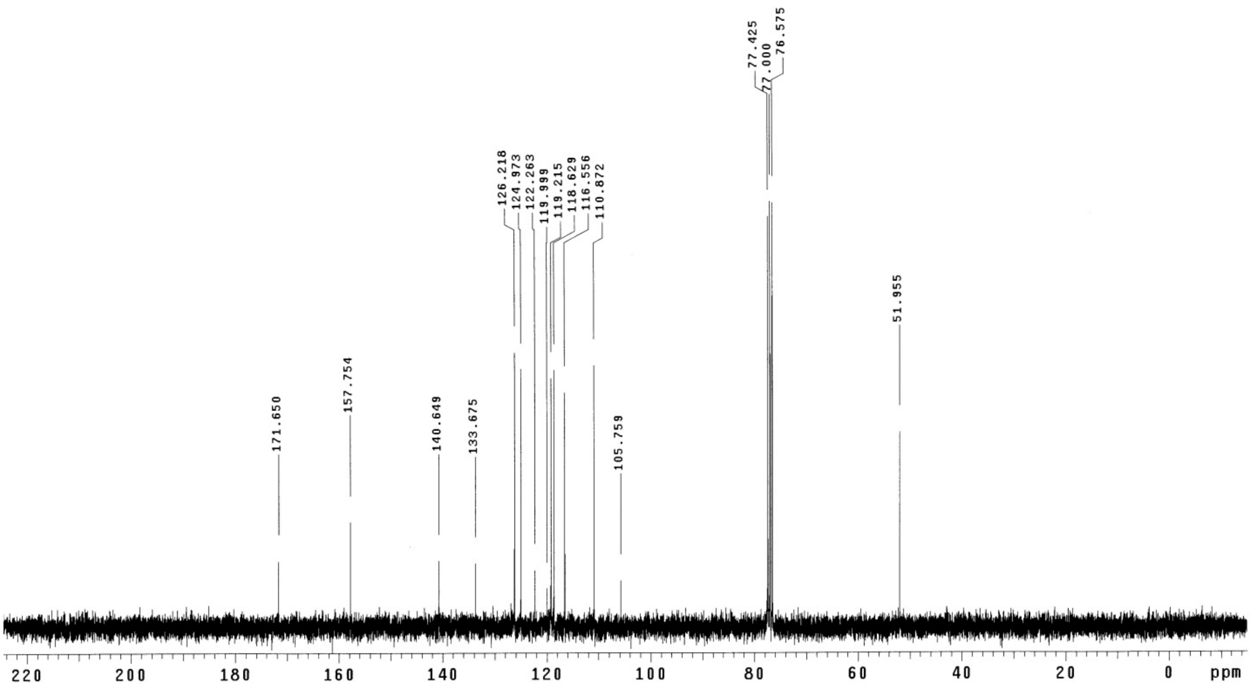

Compound-**3b**
^{1}H NMR/$CDCl_3$

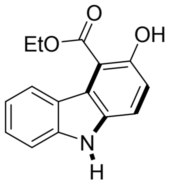

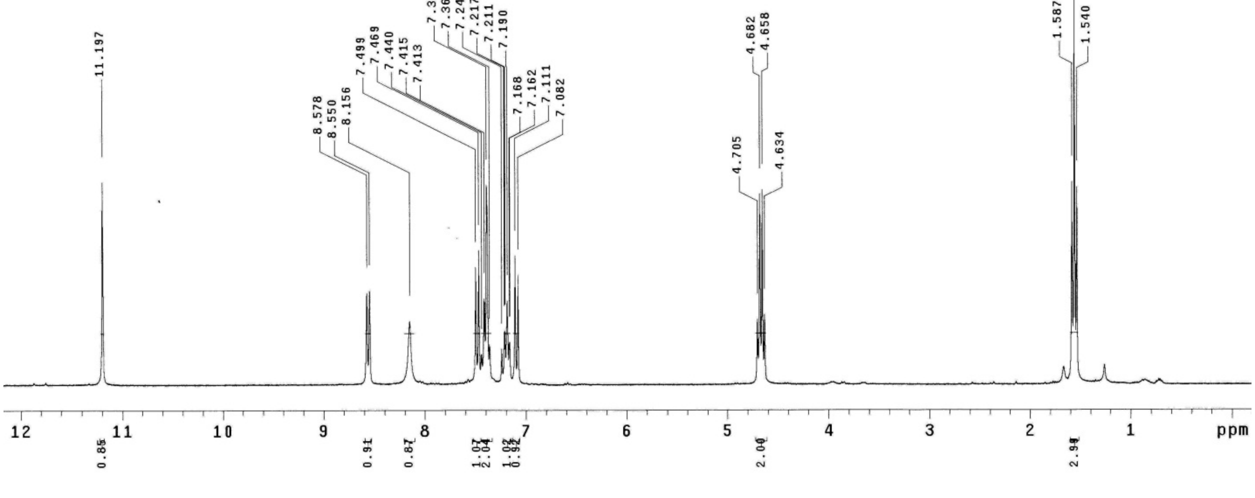

Compound-**3b**
^{13}C NMR/$CDCl_3$

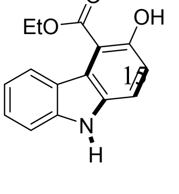

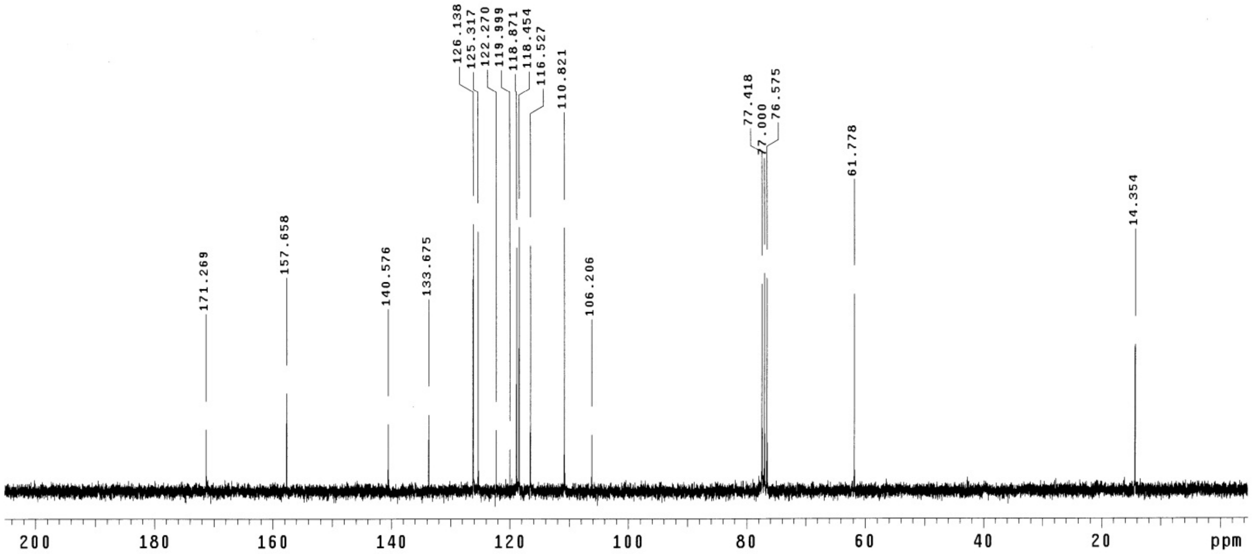

Compound-**3c**
^{1}H NMR/$CDCl_3$

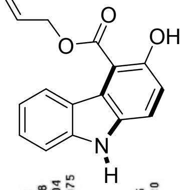

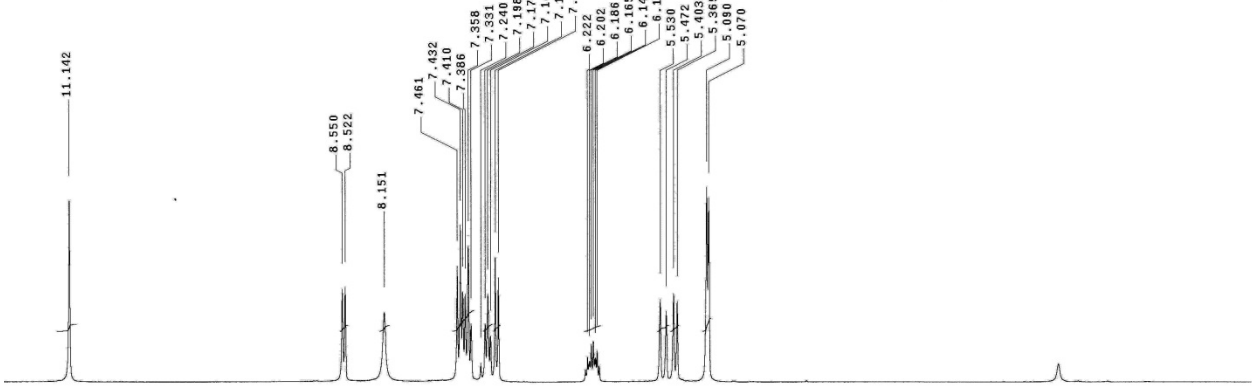

Compound-**3c**
^{1}H NMR/$CDCl_3$

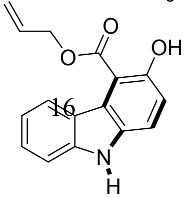

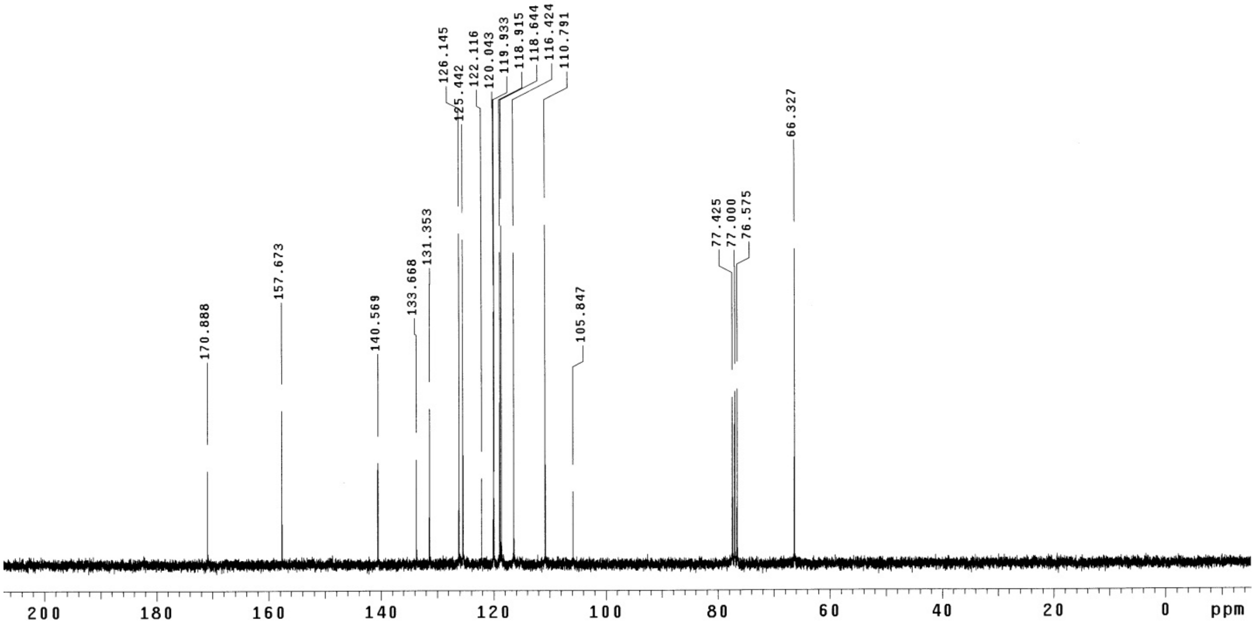

Compound-**3d**

^{1}H NMR/$CDCl_3$

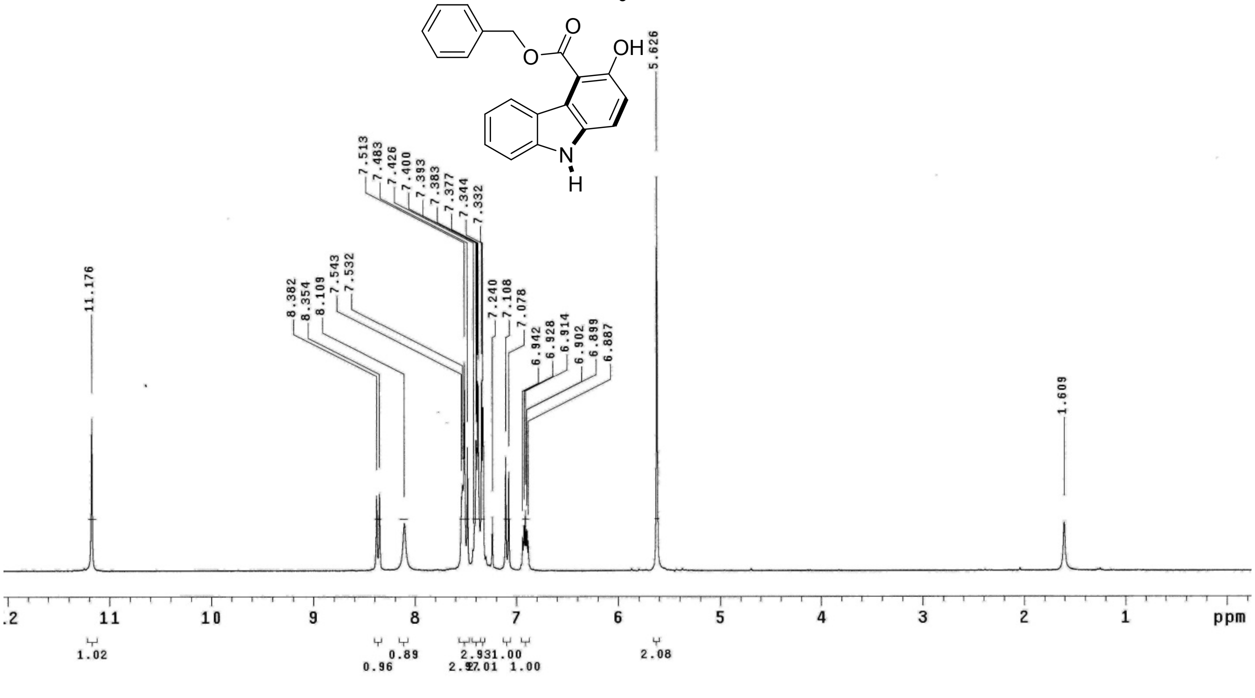

Compound-**3d**

^{13}C NMR/$CDCl_3$

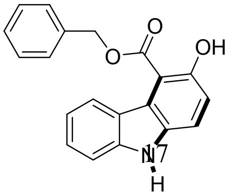

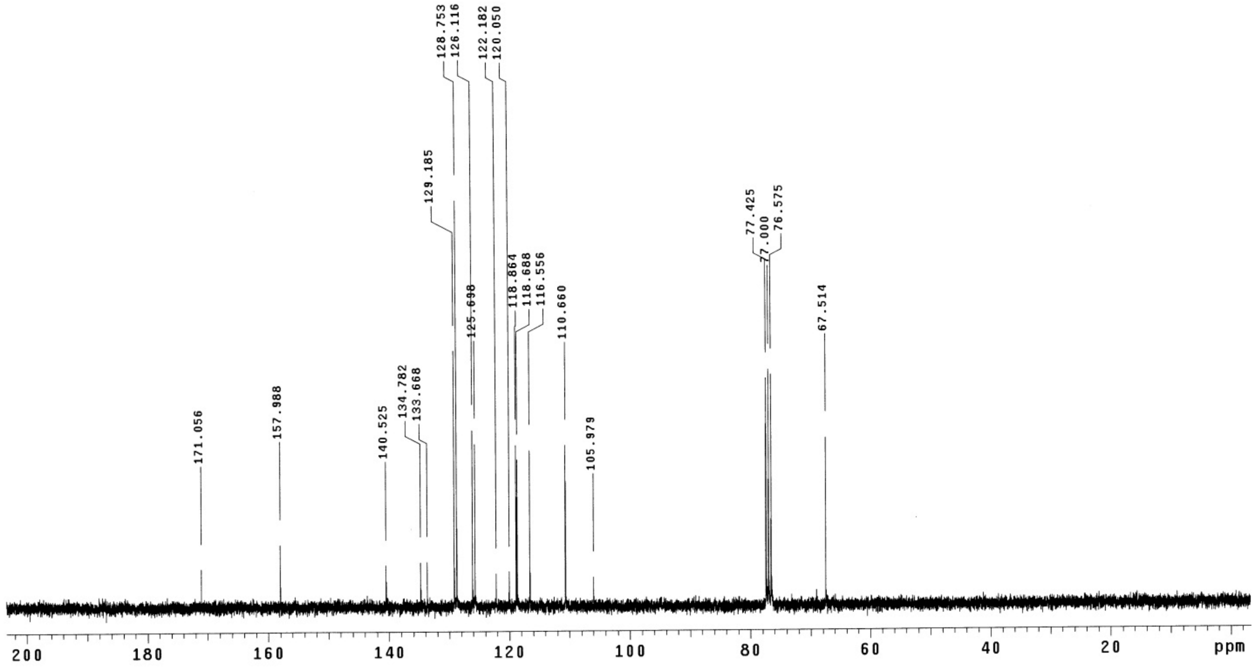

Compound-**3e**
^{1}H NMR/$CDCl_3$

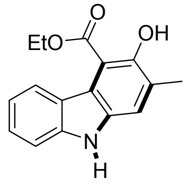

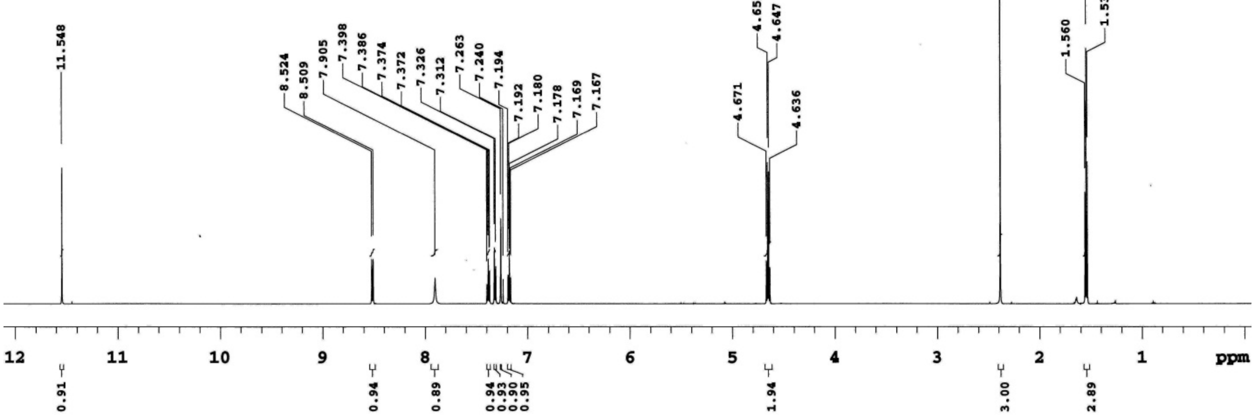

Compound-**3e**
^{13}C NMR/$CDCl_3$

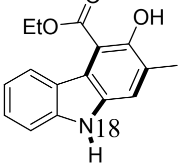

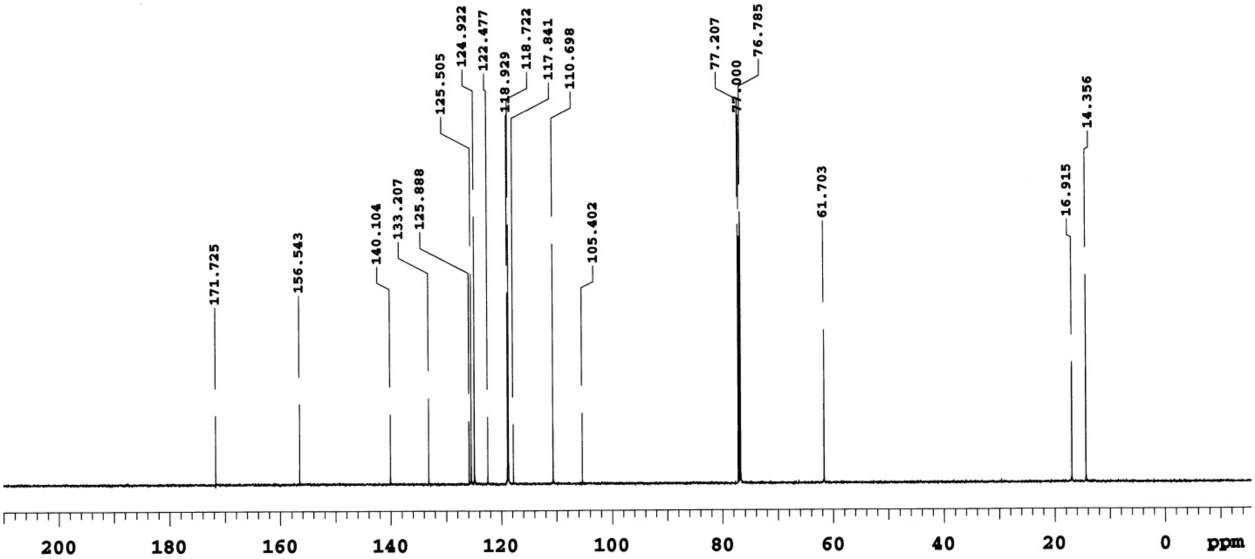

Compound-**3f**
^{1}H NMR/CDCl$_3$ +DMSO-d_6

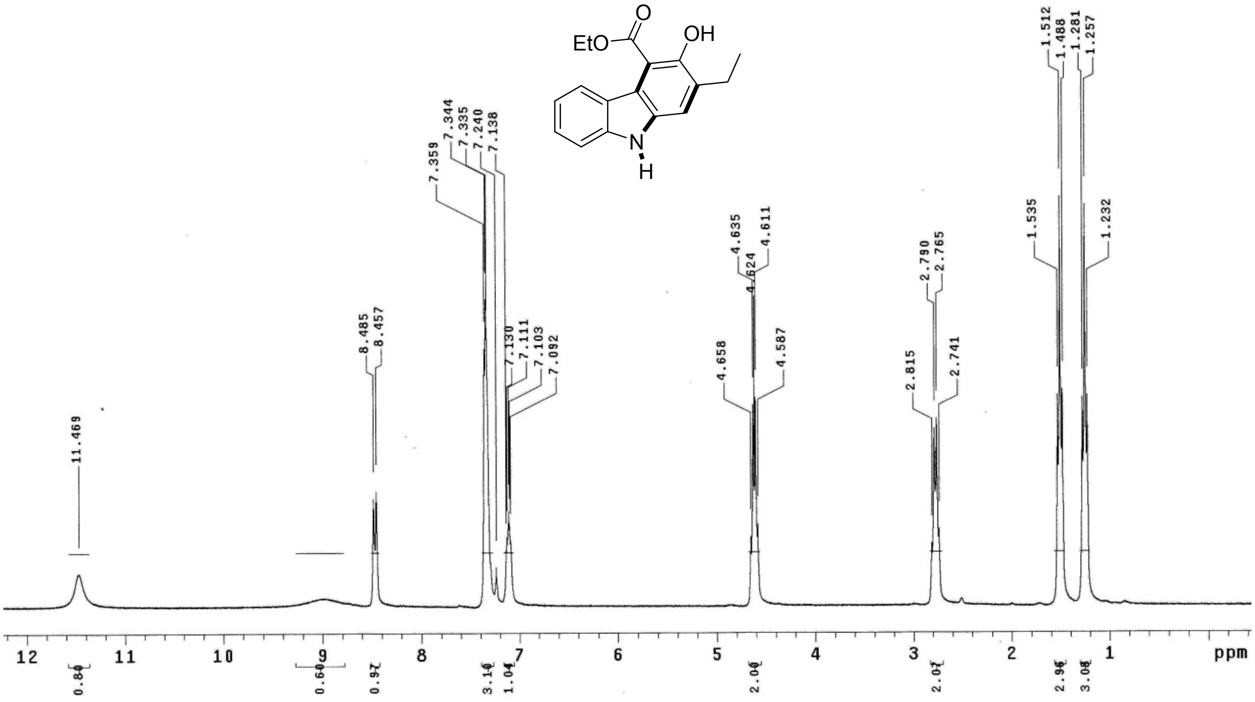

Compound-**3f**
^{13}C NMR/CDCl$_3$+DMSO-d_6

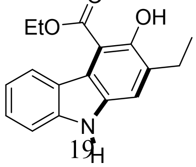

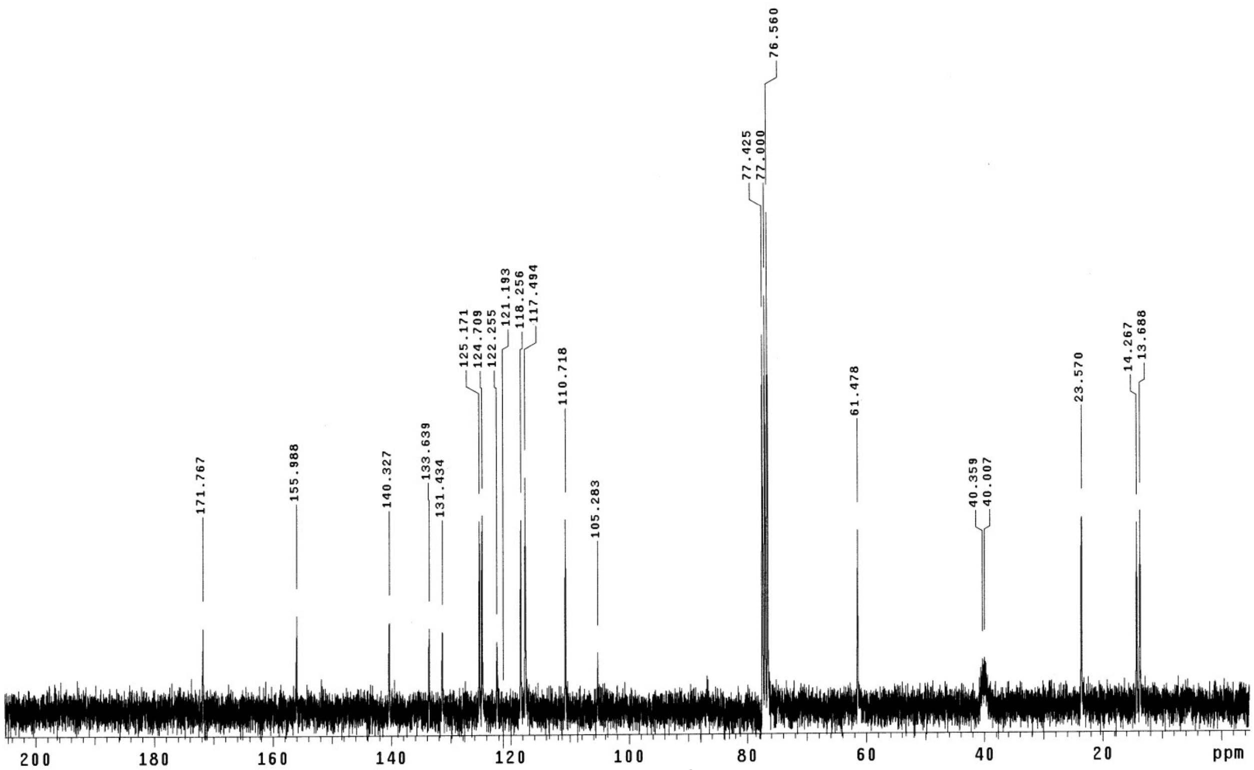

Compound-**3g**

^{1}H NMR/$CDCl_3$

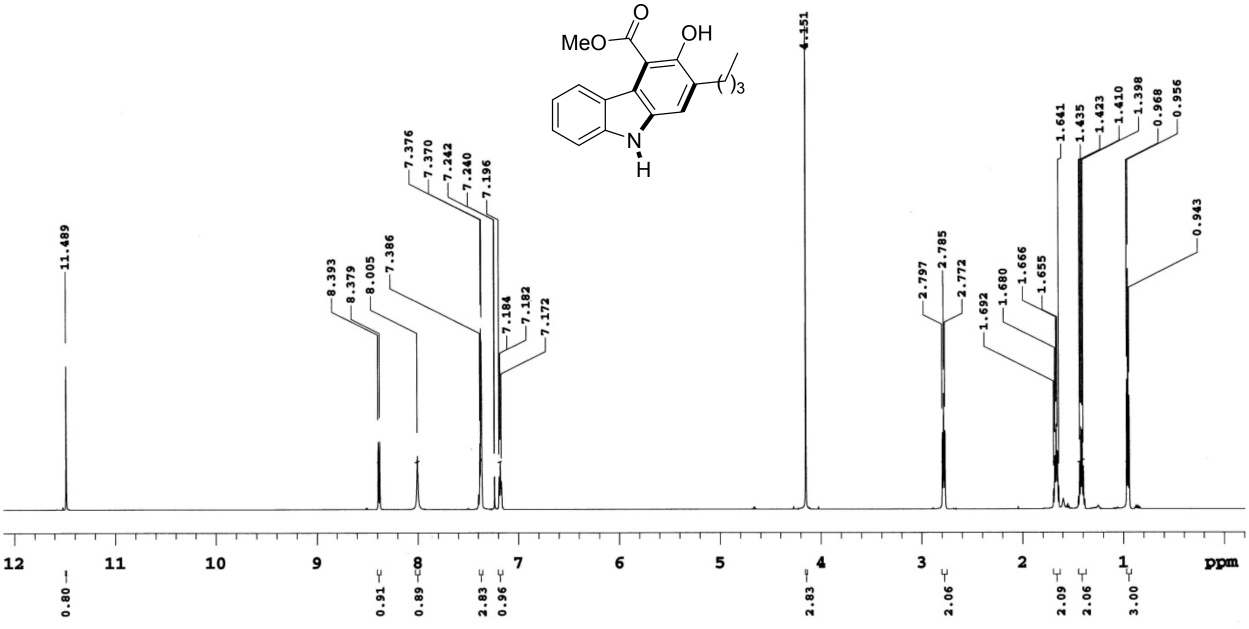

Compound-**3g**

^{13}C NMR/$CDCl_3$

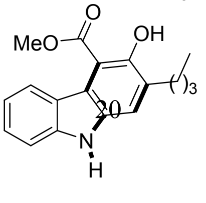

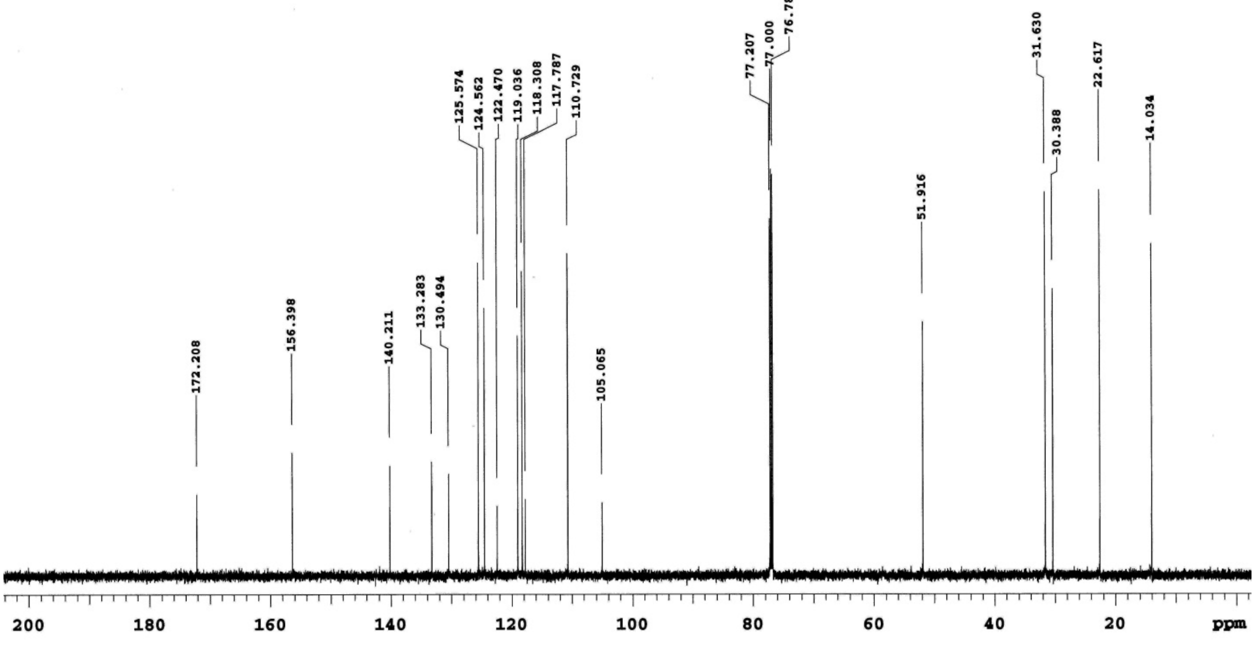

Compound-**3h**
^{1}H NMR/$CDCl_3$

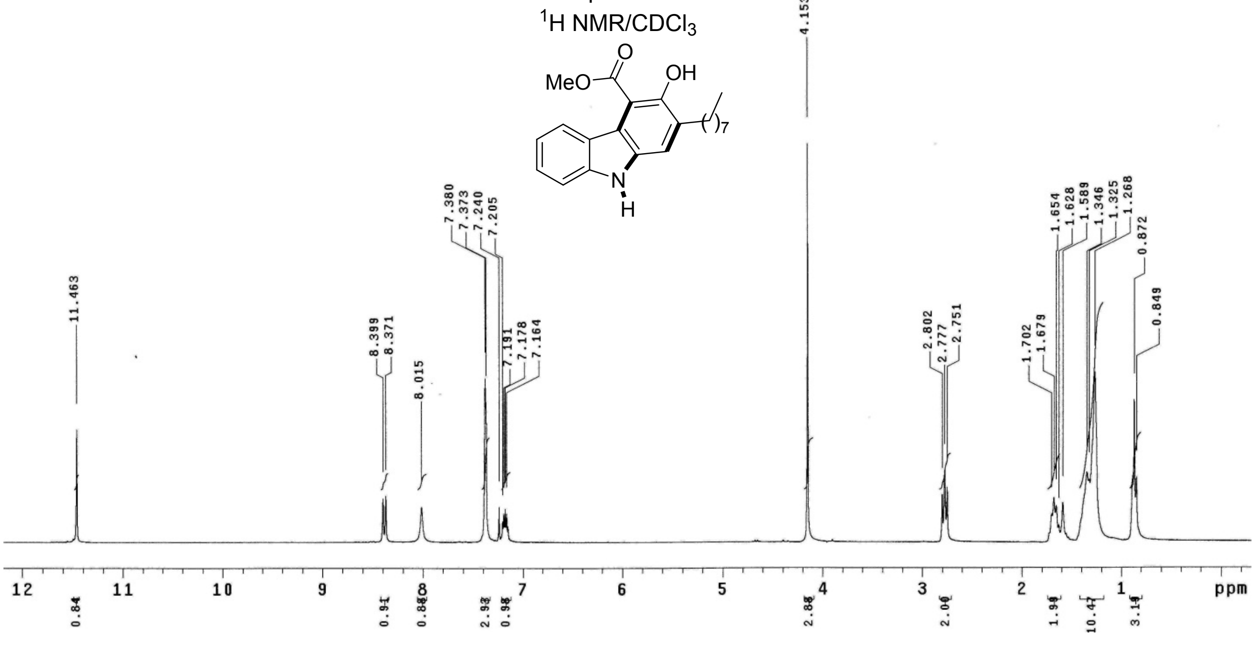

Compound-**3h**
^{13}C NMR/$CDCl_3$

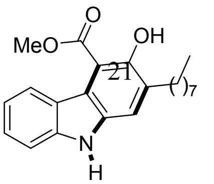

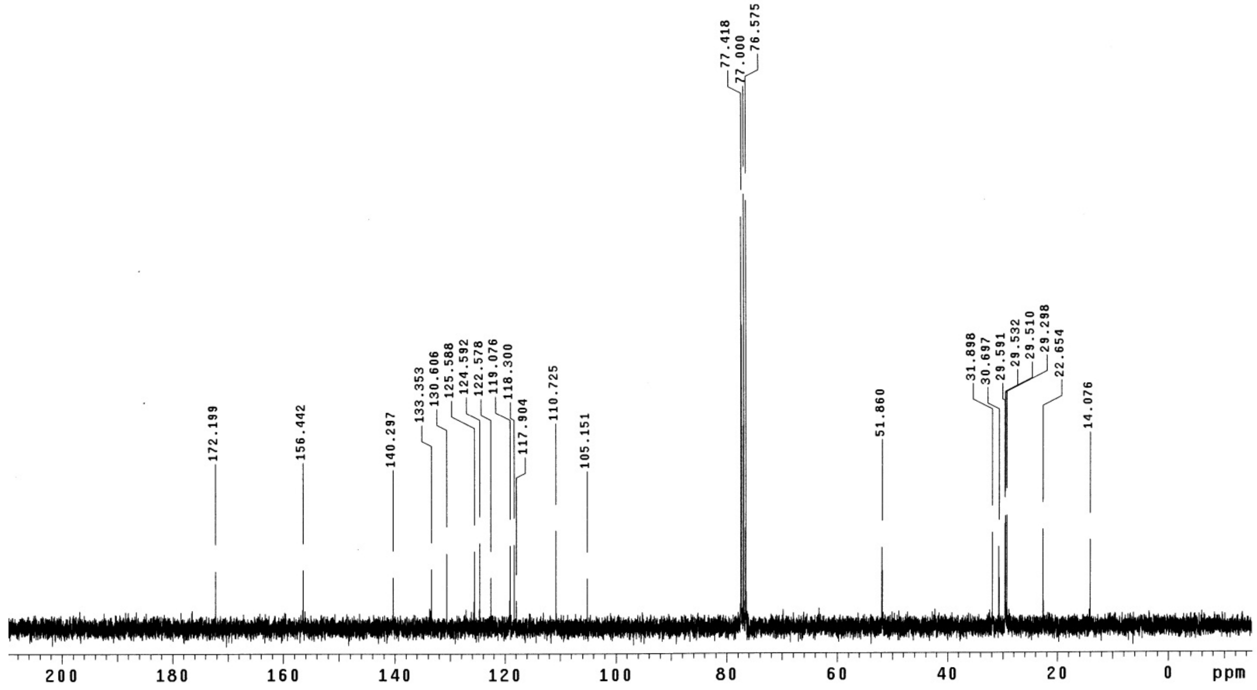

Compound-**3i**
^{1}H NMR/CDCl$_3$ + DMSO-d_6

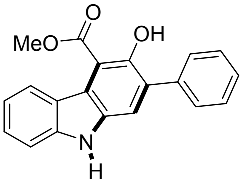

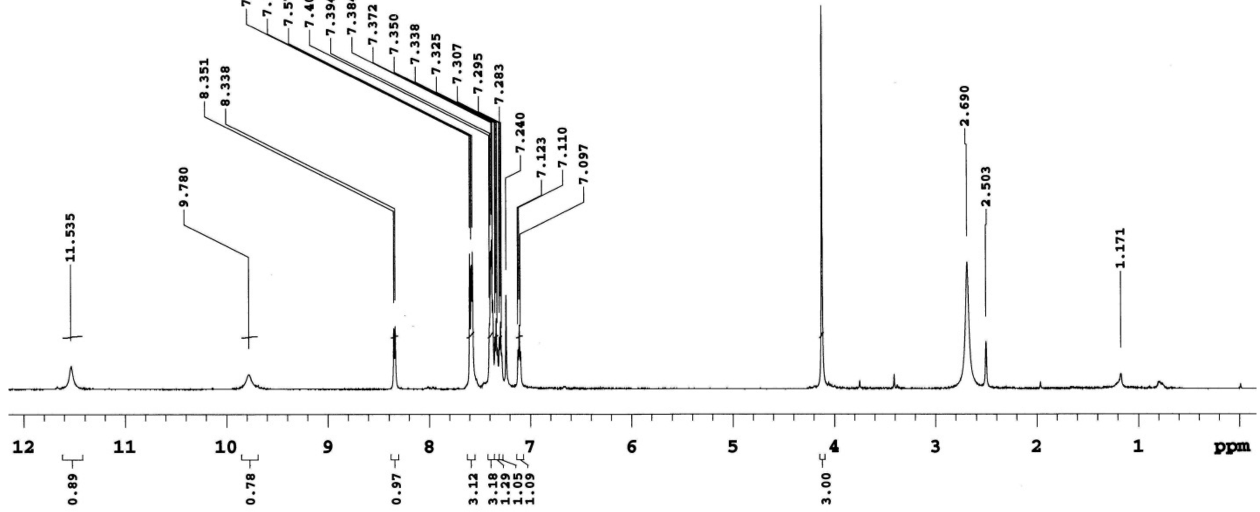

Compound-**3i**
^{13}C NMR/CDCl$_3$ + DMSO-d_6

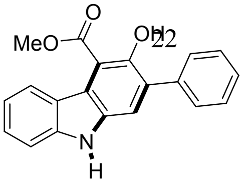

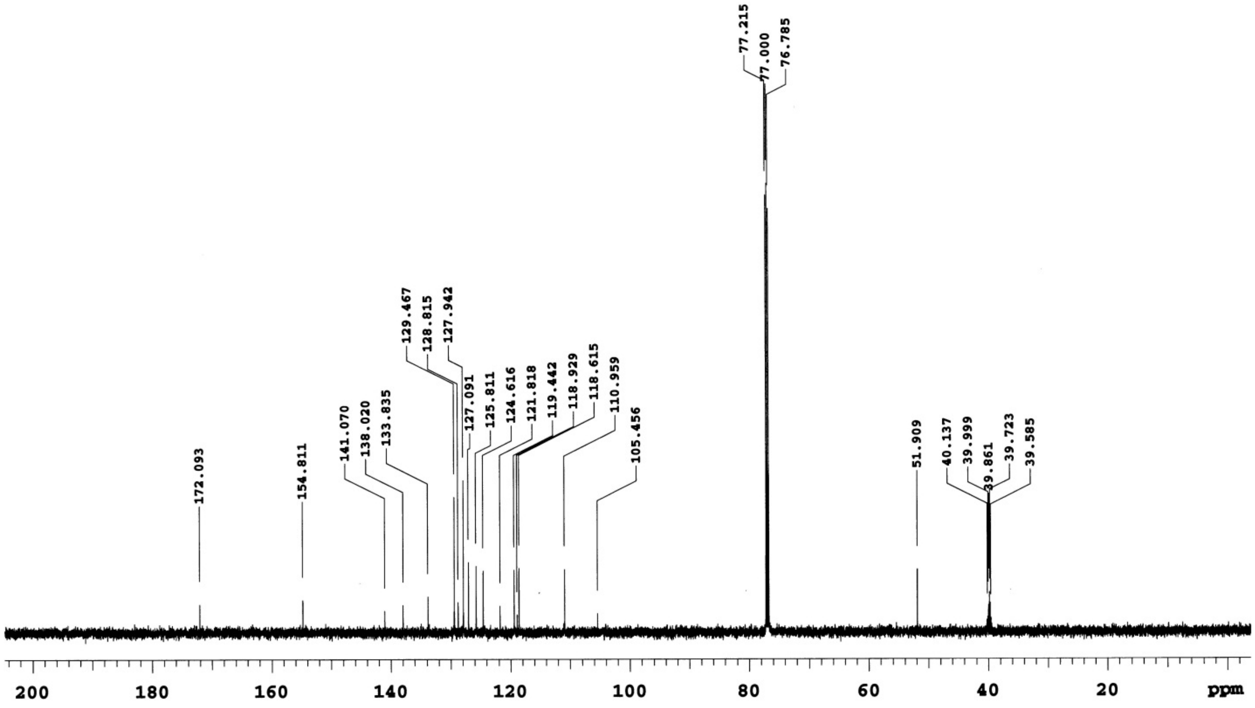

Compound-**5a**

^{1}H NMR/$CDCl_3$

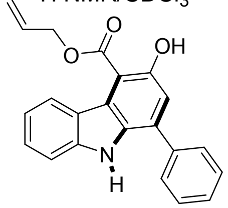

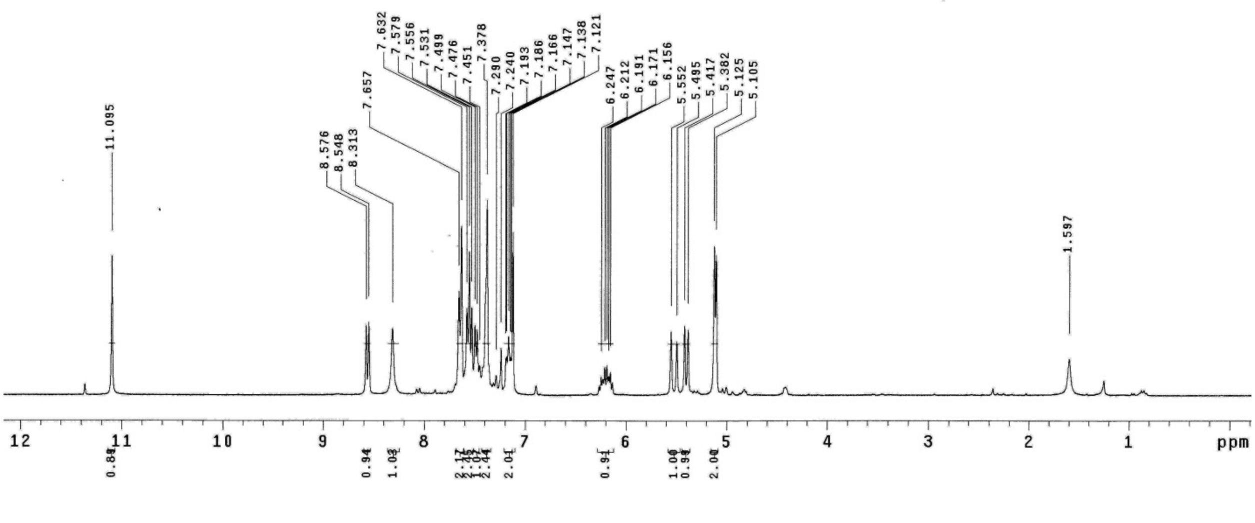

Compound-**5a**

^{13}C NMR/$CDCl_3$

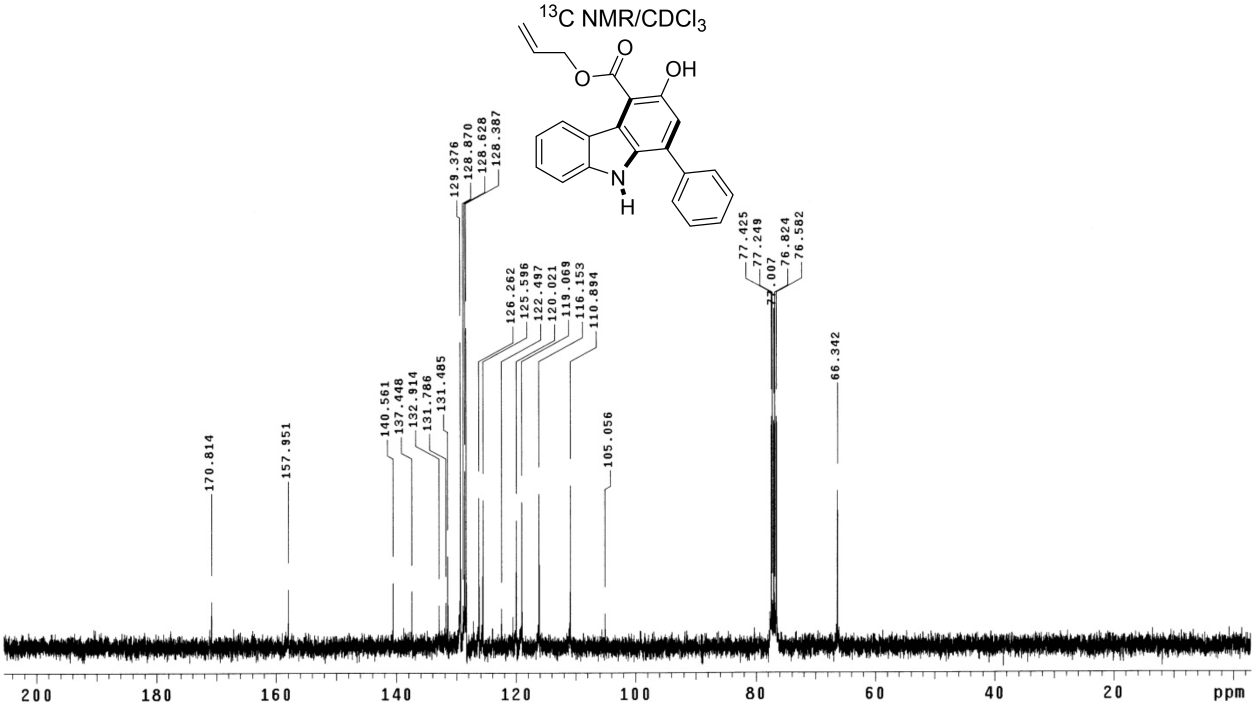

Compound-**5b**

^{1}H NMR/$CDCl_3$

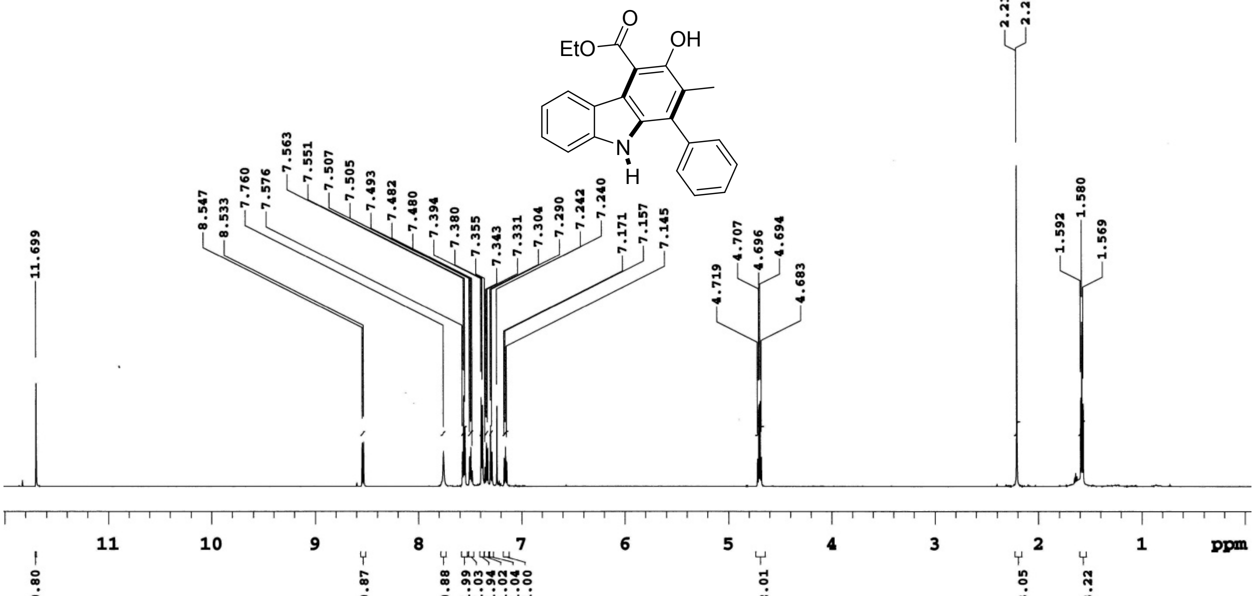

Compound-**5b**

^{13}C NMR/$CDCl_3$

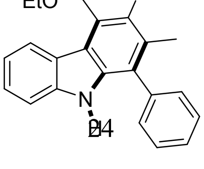

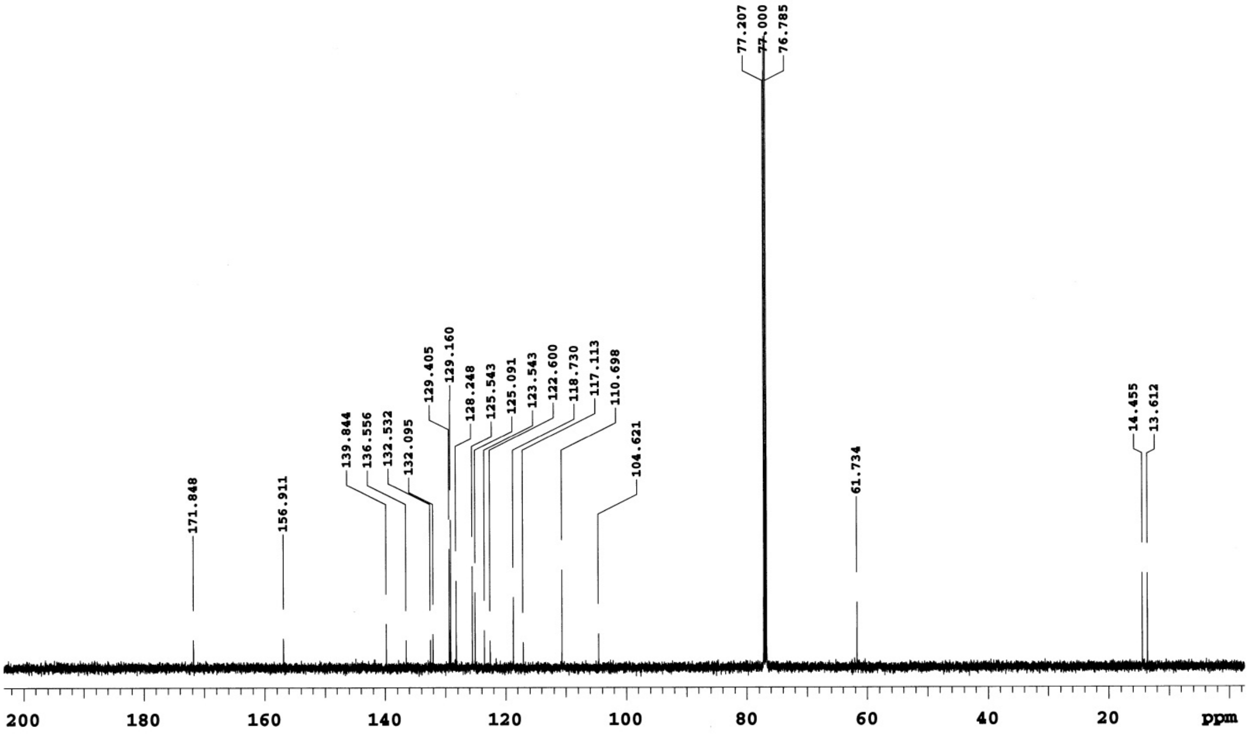

77.207
77.000
76.785
129.405
129.160
128.248
125.543
125.091
123.543
122.600
118.730
117.113
110.698
139.844
136.556
132.532
132.095
104.621
171.848
156.911
61.734
14.455
13.612
200
180
160
140
120
100
80
60
40
20
ppm

Compound-**5c**
^{1}H NMR/$CDCl_3$

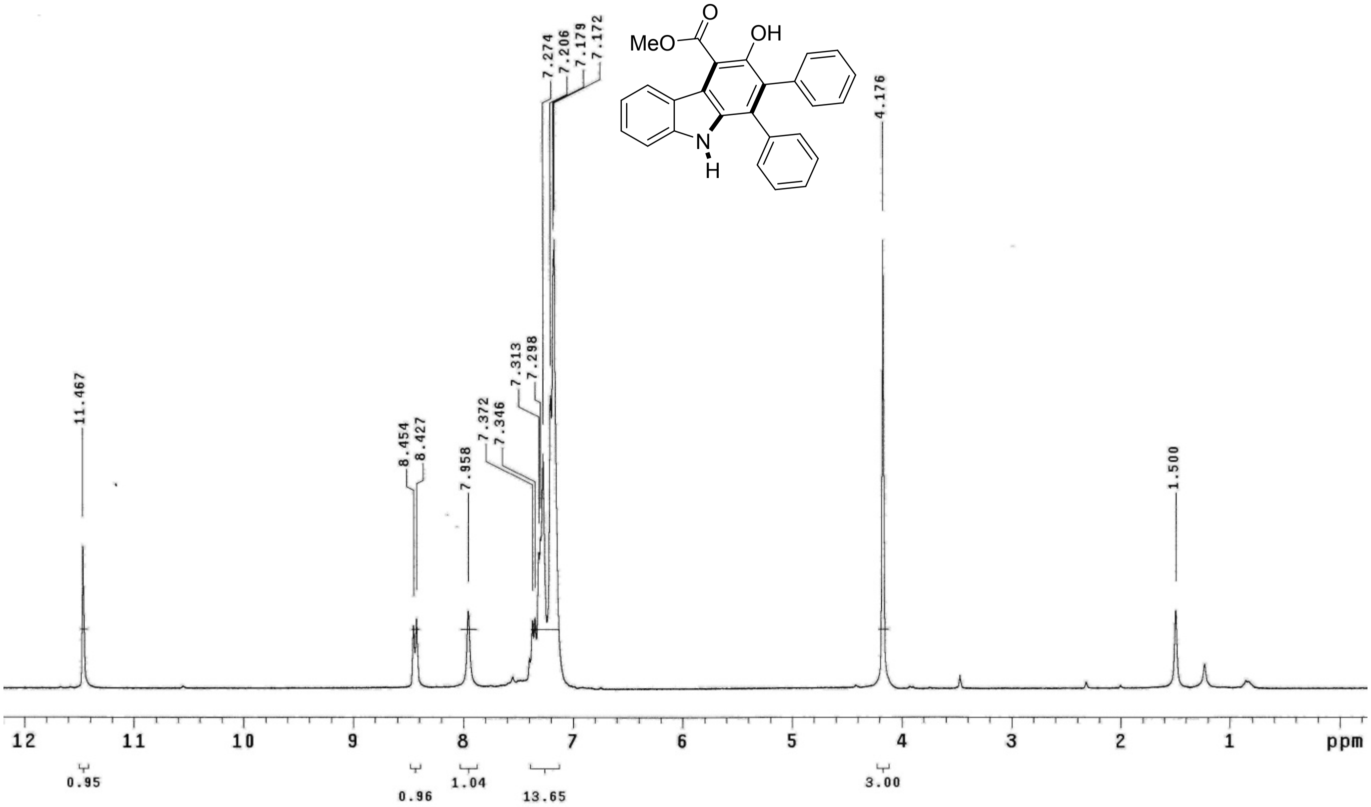

Compound-**5c**
^{13}C NMR/$CDCl_3$

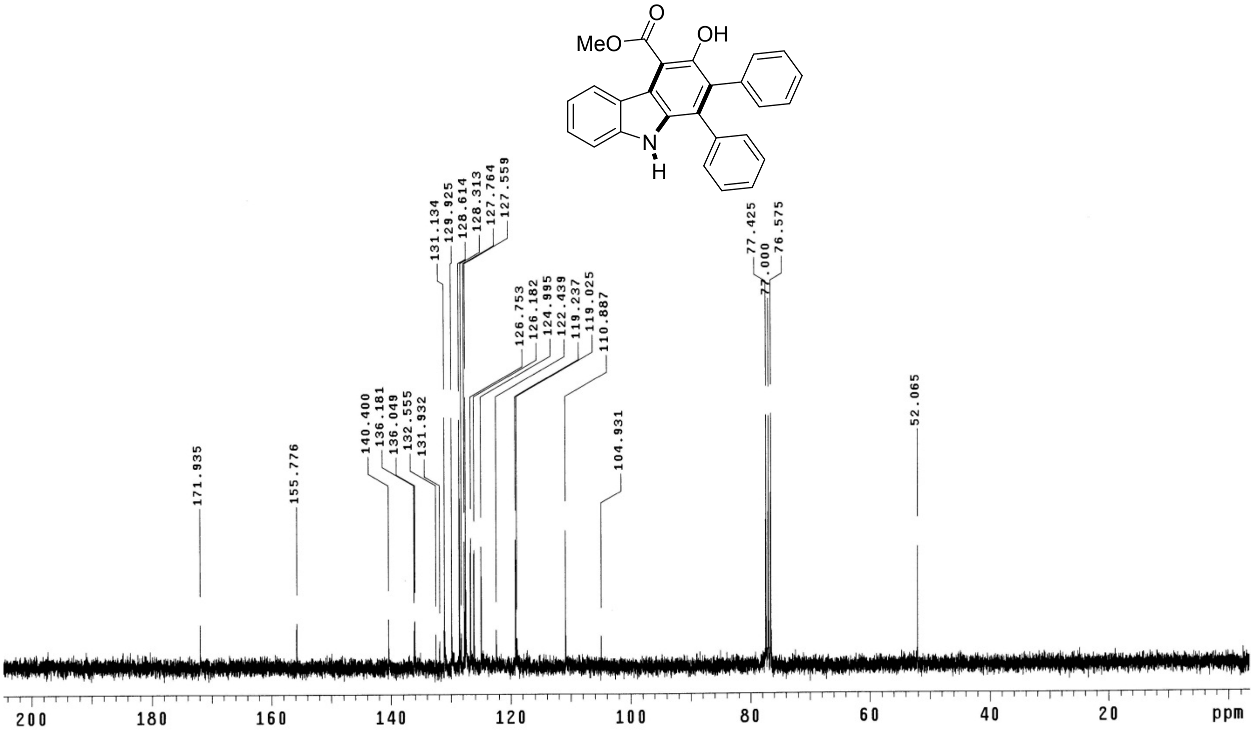

Compound-**5d**
^{1}H NMR/$CDCl_3$

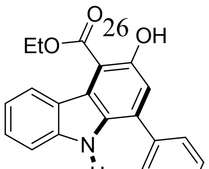

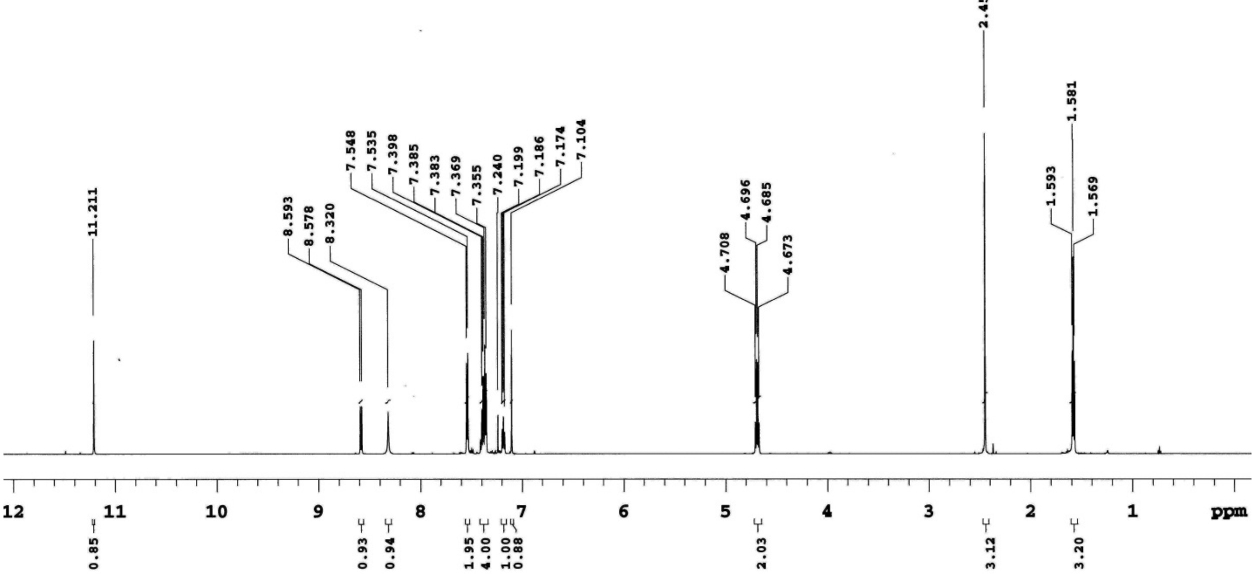

Compound-**5d**

^{13}C NMR/$CDCl_3$

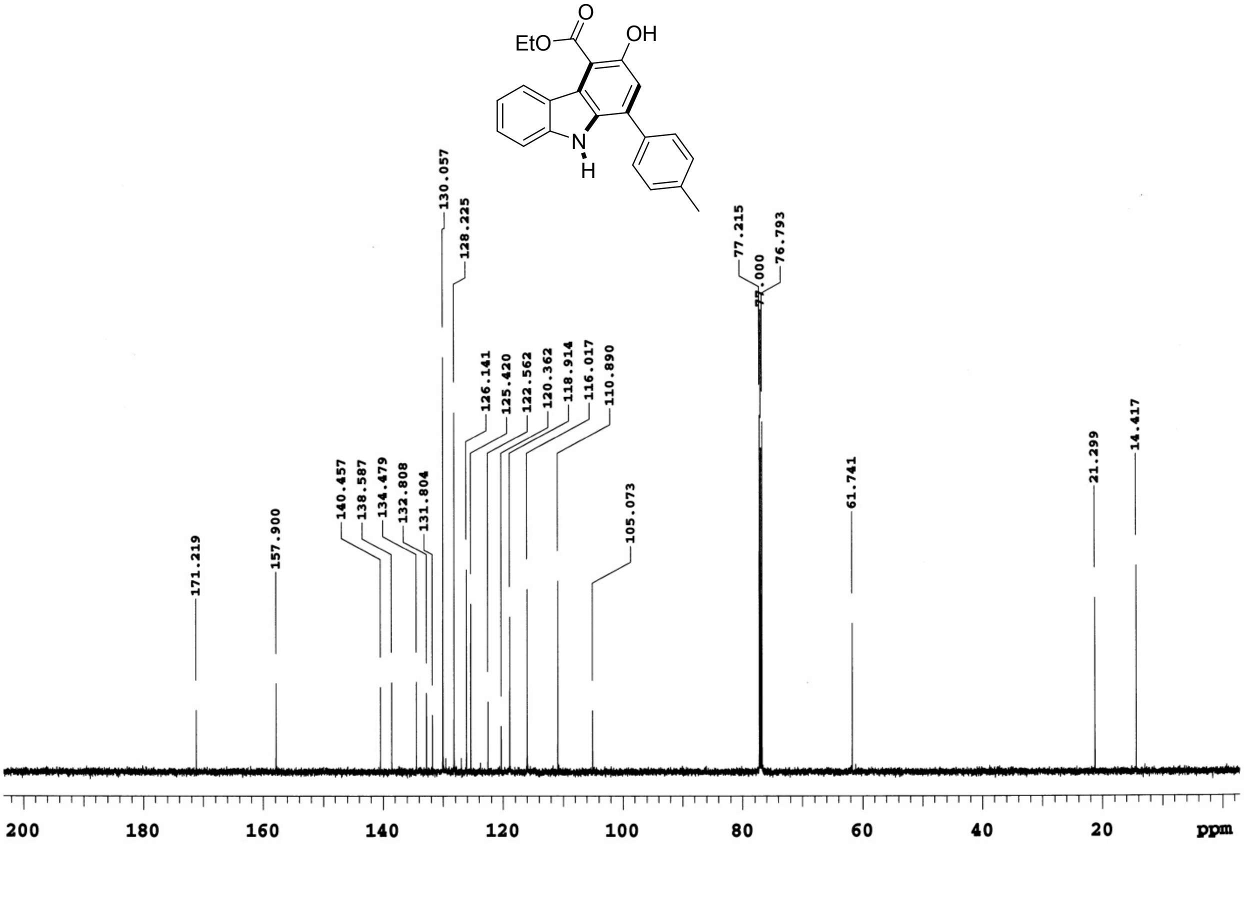

Compound-**5e**
^{1}H NMR/$CDCl_3$

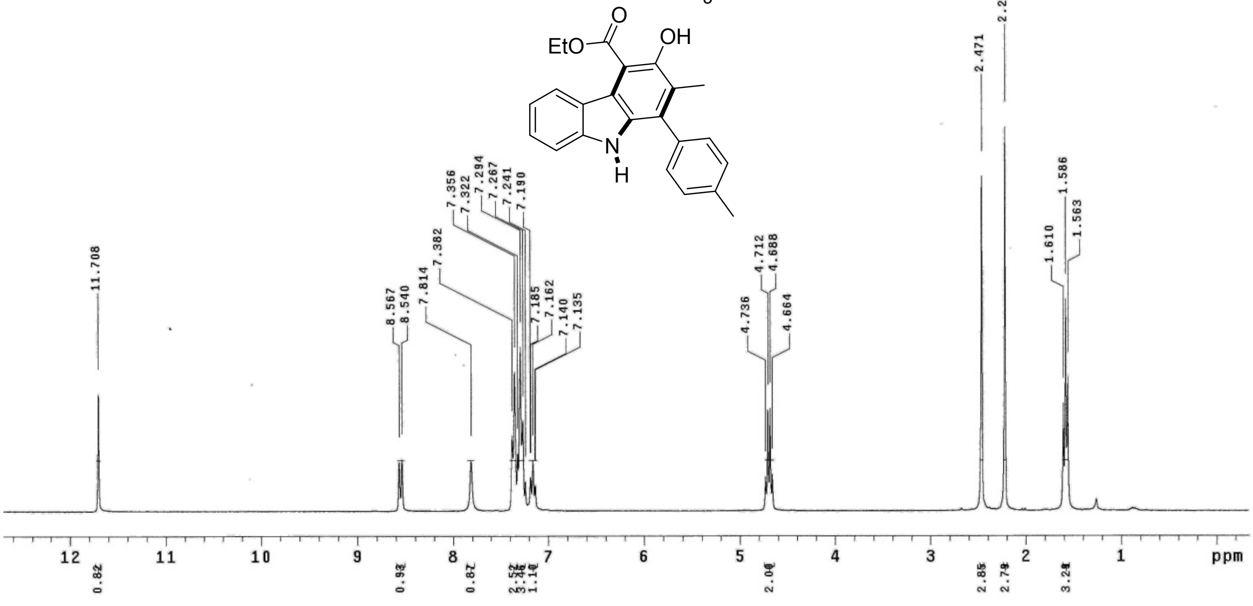

Compound-**5e**
^{13}C NMR/$CDCl_3$

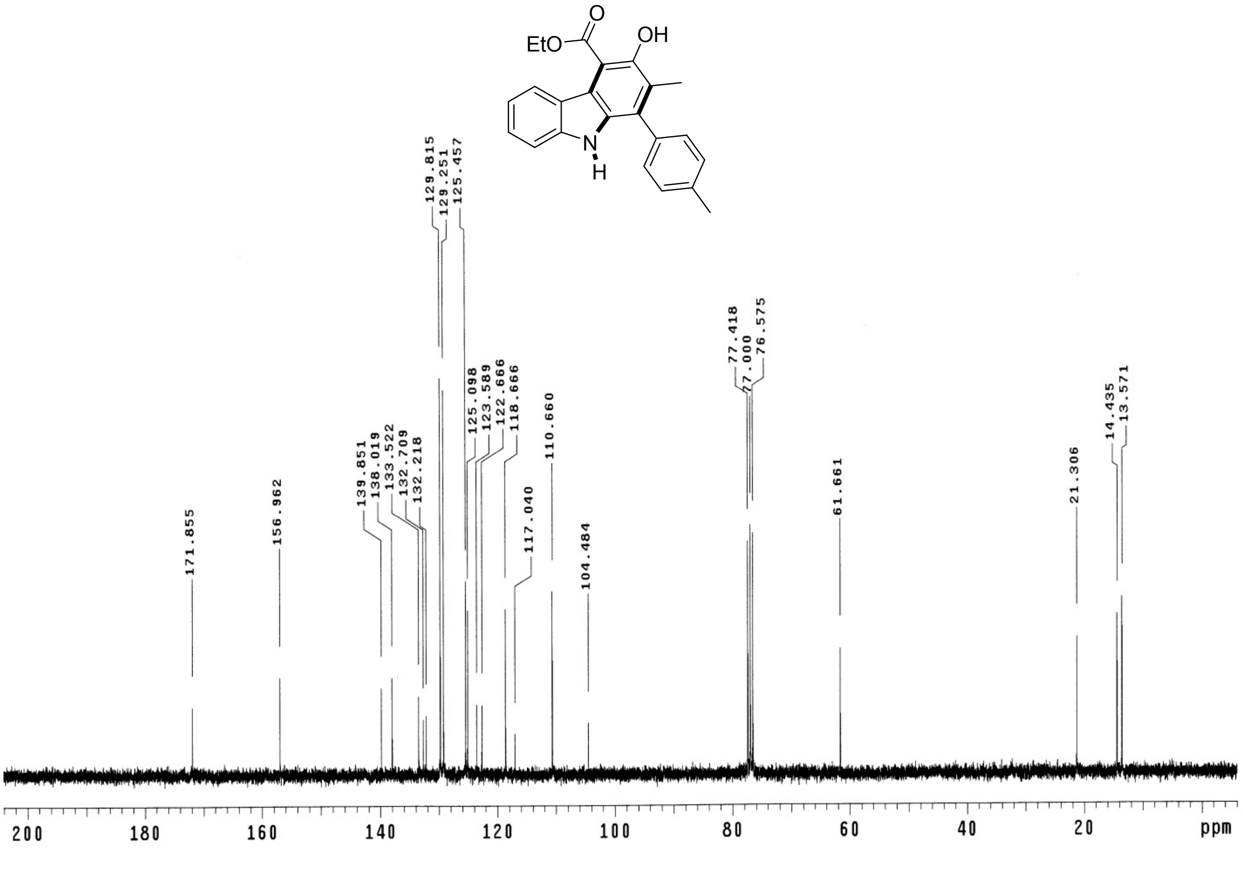

Compound-**5f**
^{1}H NMR/$CDCl_3$

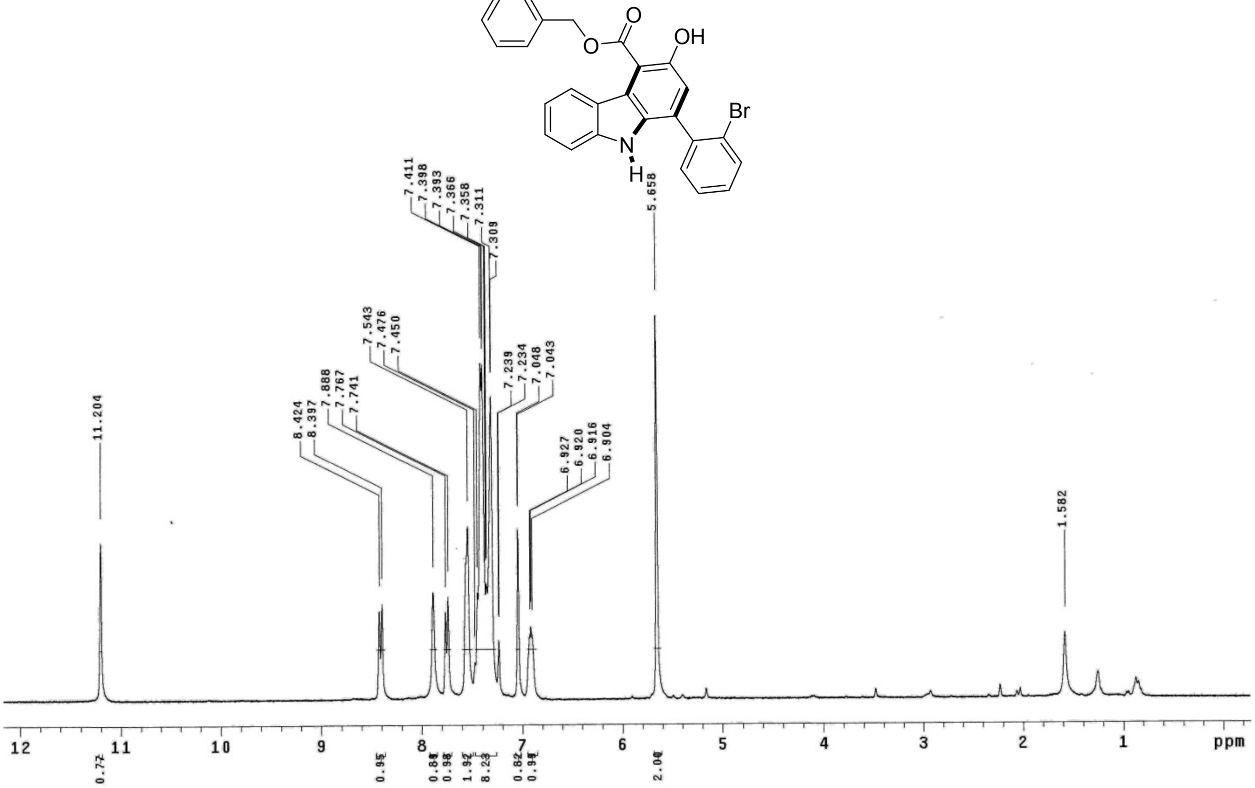

Compound-**5f**
^{13}C NMR/$CDCl_3$

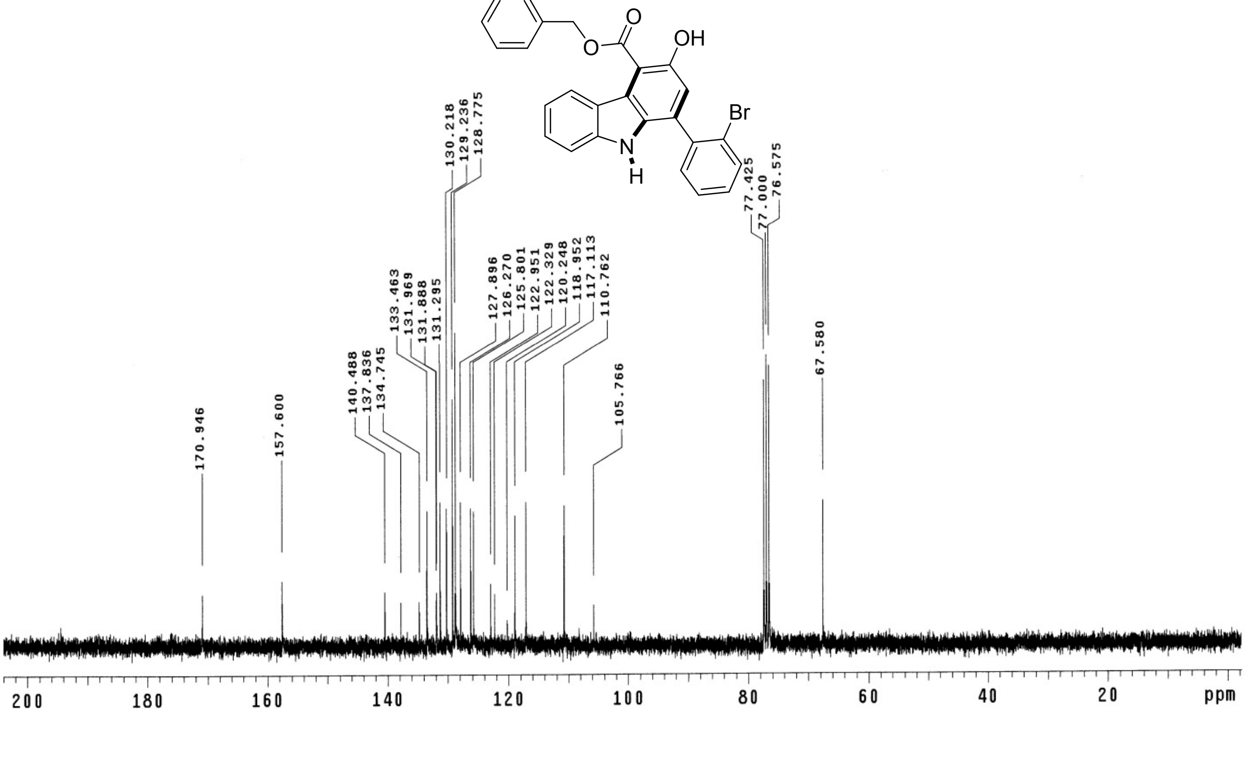

Compound-**5g**
^{1}H NMR/$CDCl_3$

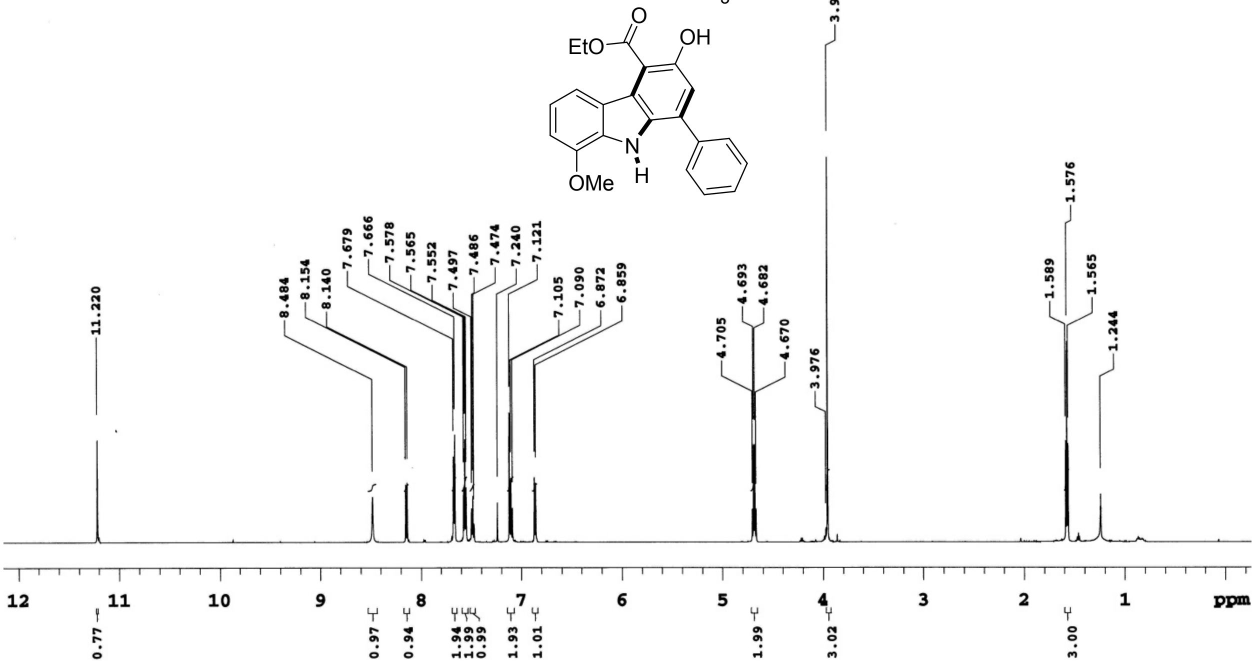

Compound-**5g**
^{13}C NMR/$CDCl_3$

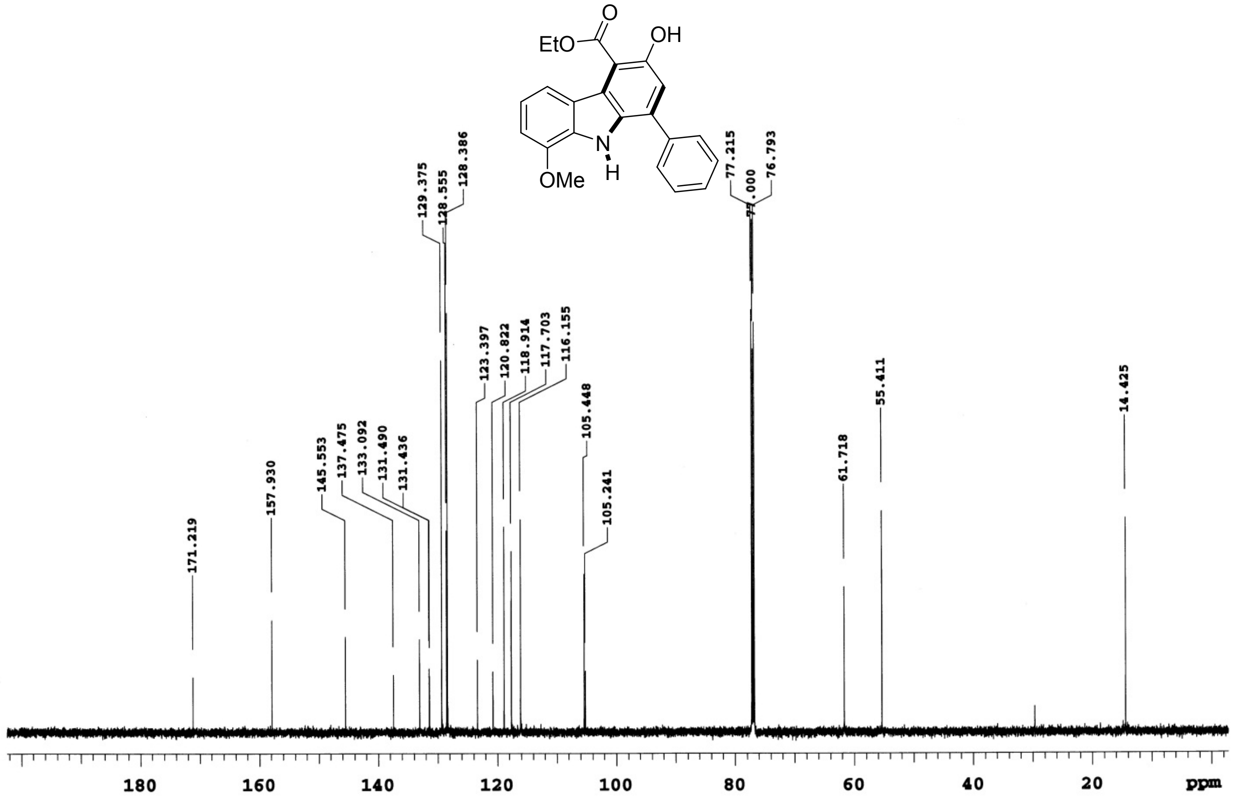

Compound-**5h**
^{1}H NMR/$CDCl_3$

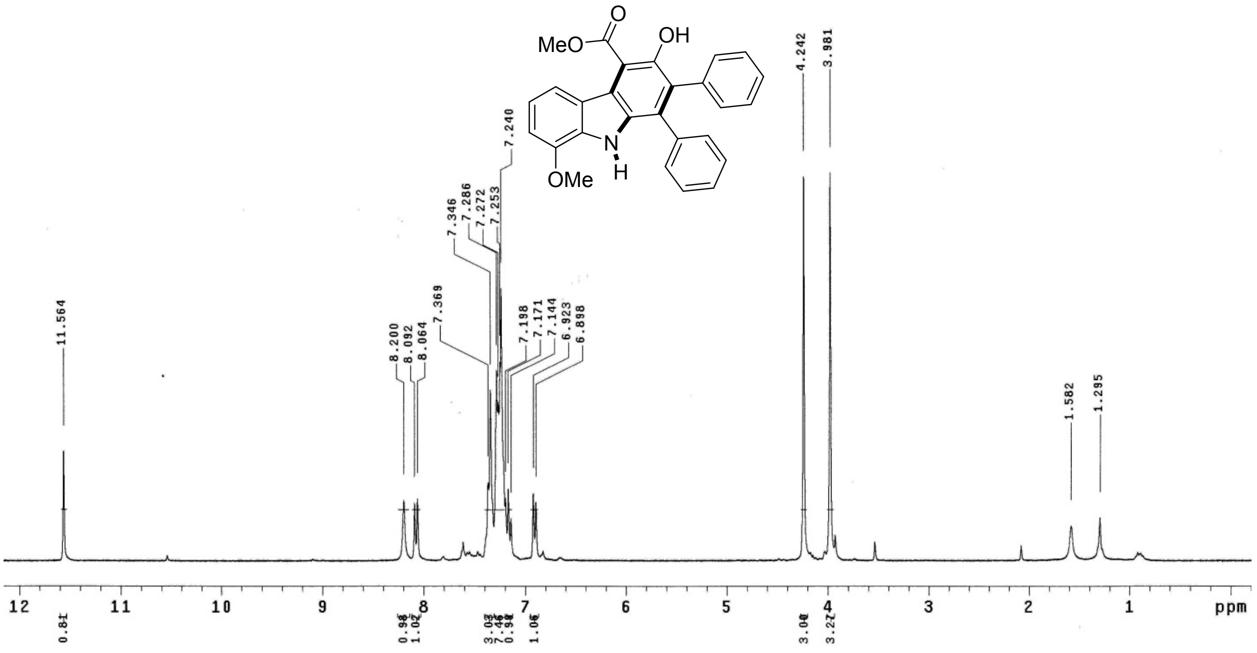

Compound-**5h**
^{13}C NMR/$CDCl_3$

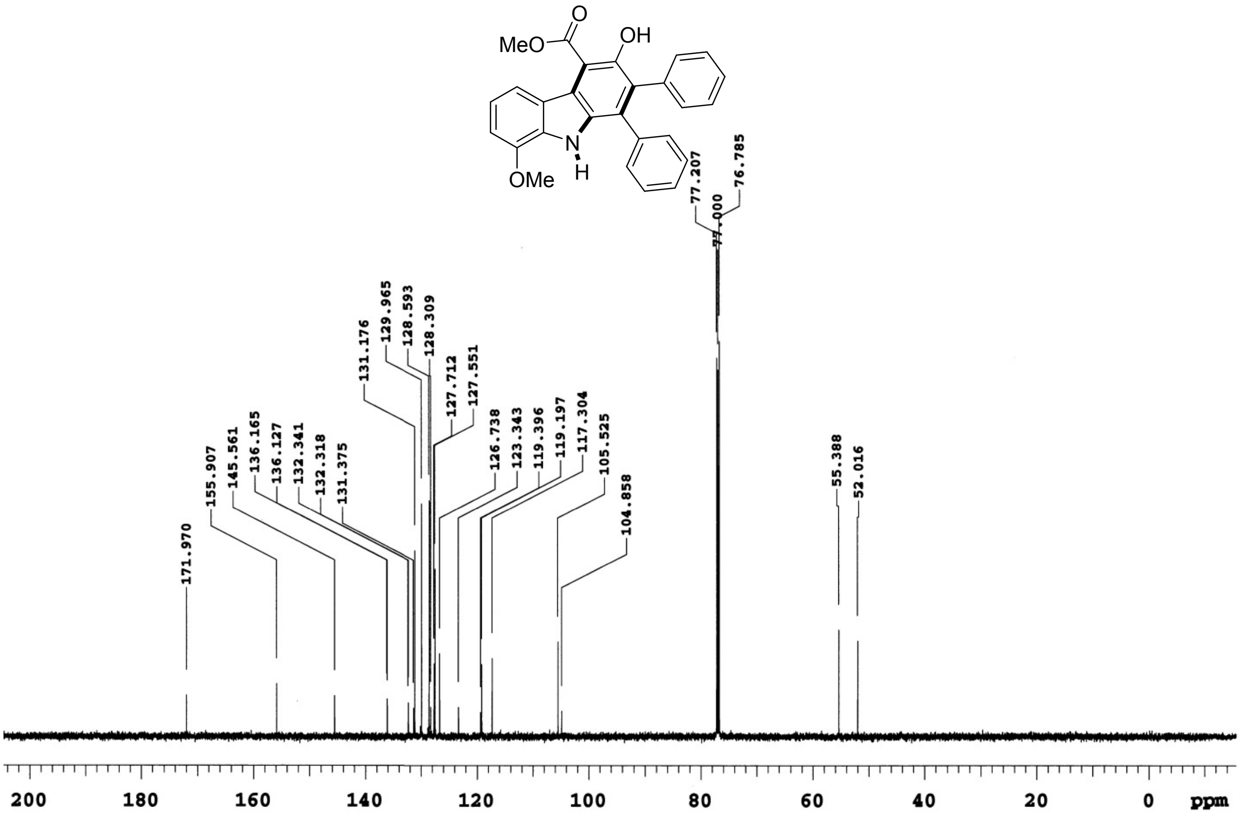

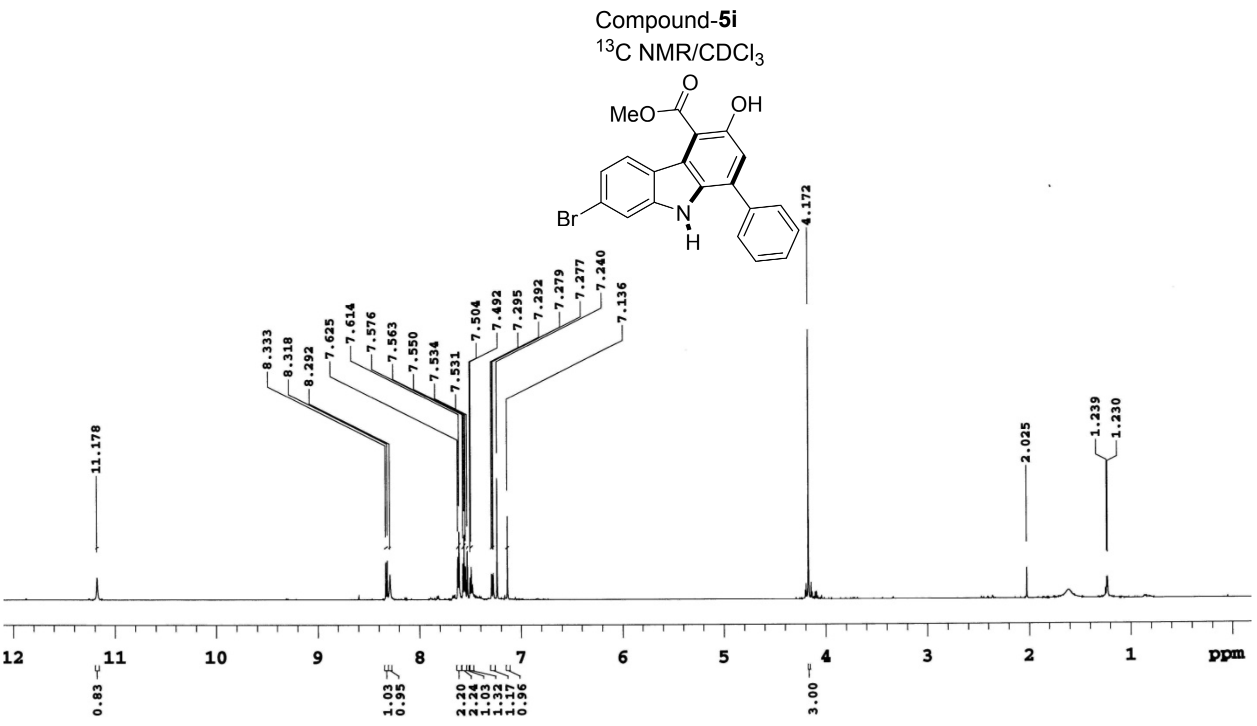
Compound-**5i**
^{13}C NMR/CDCl$_3$
MeO
O
OH
Br
N
H
11.178
8.333
8.318
8.292
7.625
7.614
7.576
7.563
7.550
7.534
7.531
7.504
7.492
7.295
7.292
7.279
7.277
7.240
7.136
4.172
2.025
1.239
1.230
12 11 10 9 8 7 6 5 4 3 2 1 ppm
0.83
1.03
0.95
2.20
2.24
1.03
1.32
1.17
0.96
3.00

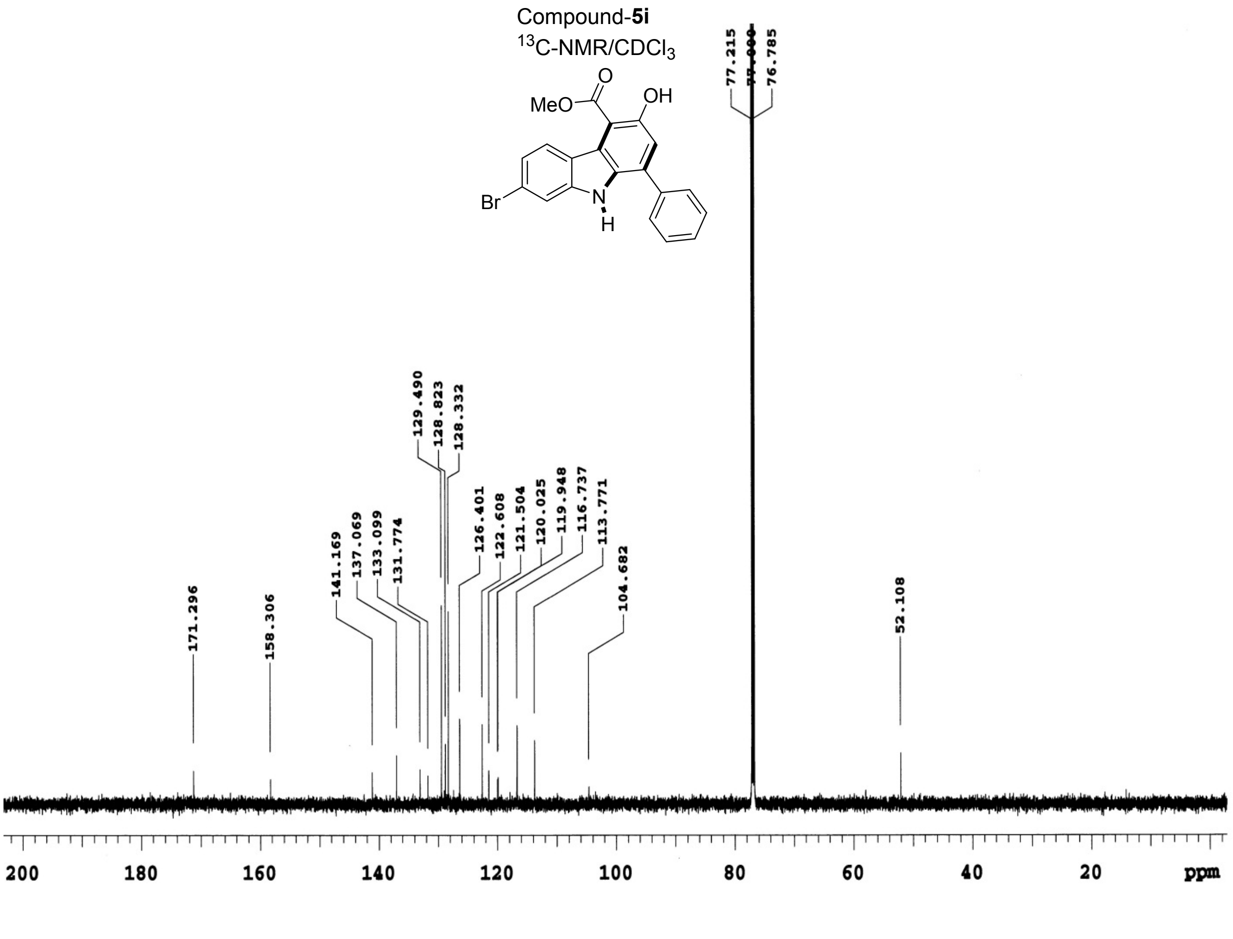
Compound-**5i**
^{13}C-NMR/CDCl$_3$
MeO
O
OH
Br
N
H
171.296
158.306
141.169
137.069
133.099
131.774
129.490
128.823
128.332
126.401
122.608
121.504
120.025
119.948
116.737
113.771
104.682
77.215
76.785
52.108
200 180 160 140 120 100 80 60 40 20 ppm

Compound-**5j**
^{1}H NMR/$CDCl_3$

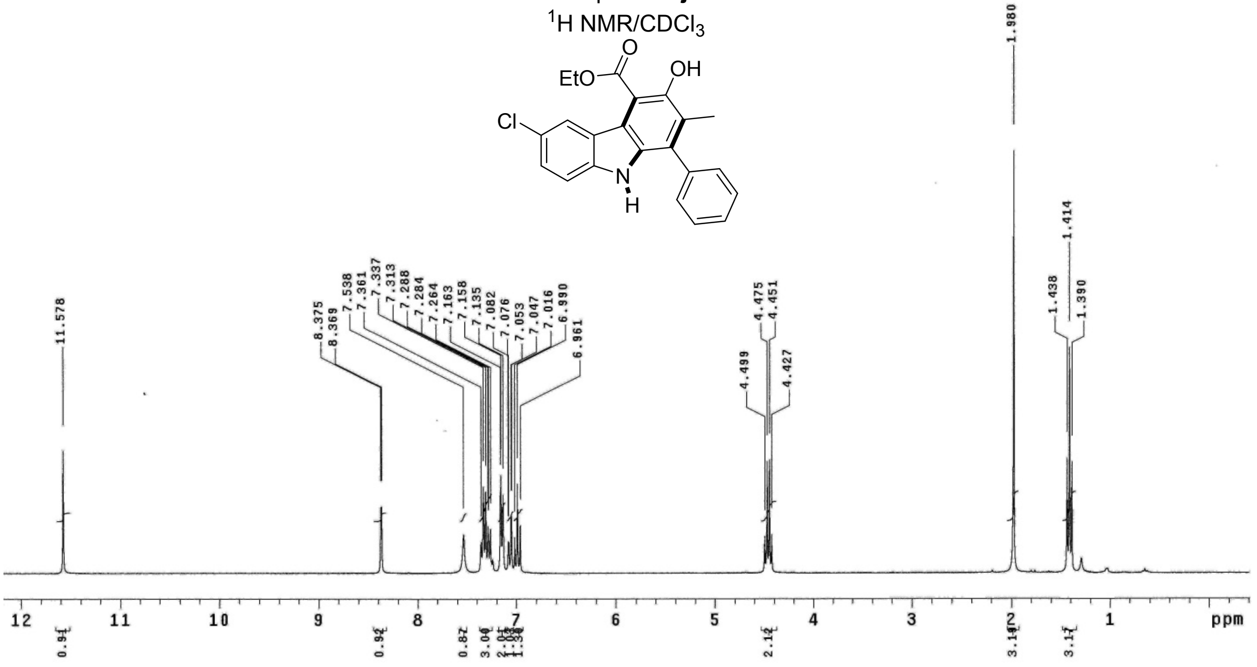

Compound-**5j**
^{13}C NMR/$CDCl_3$

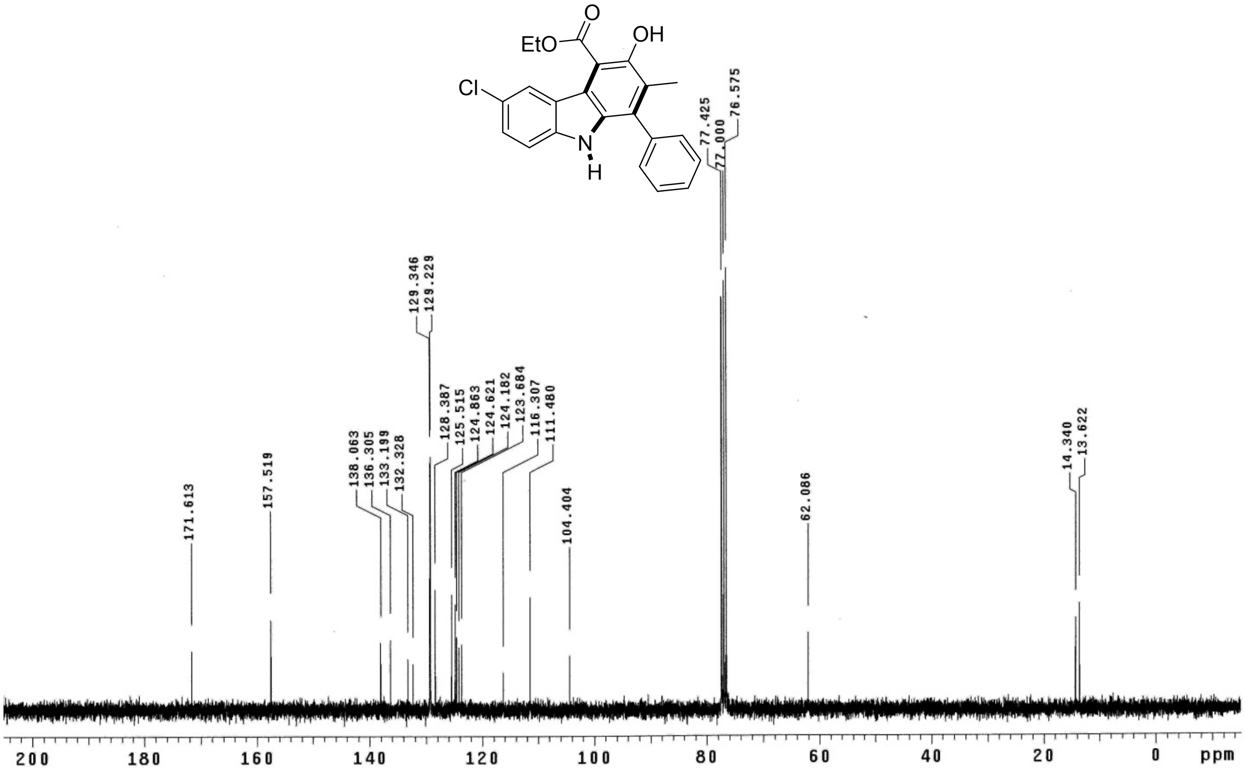

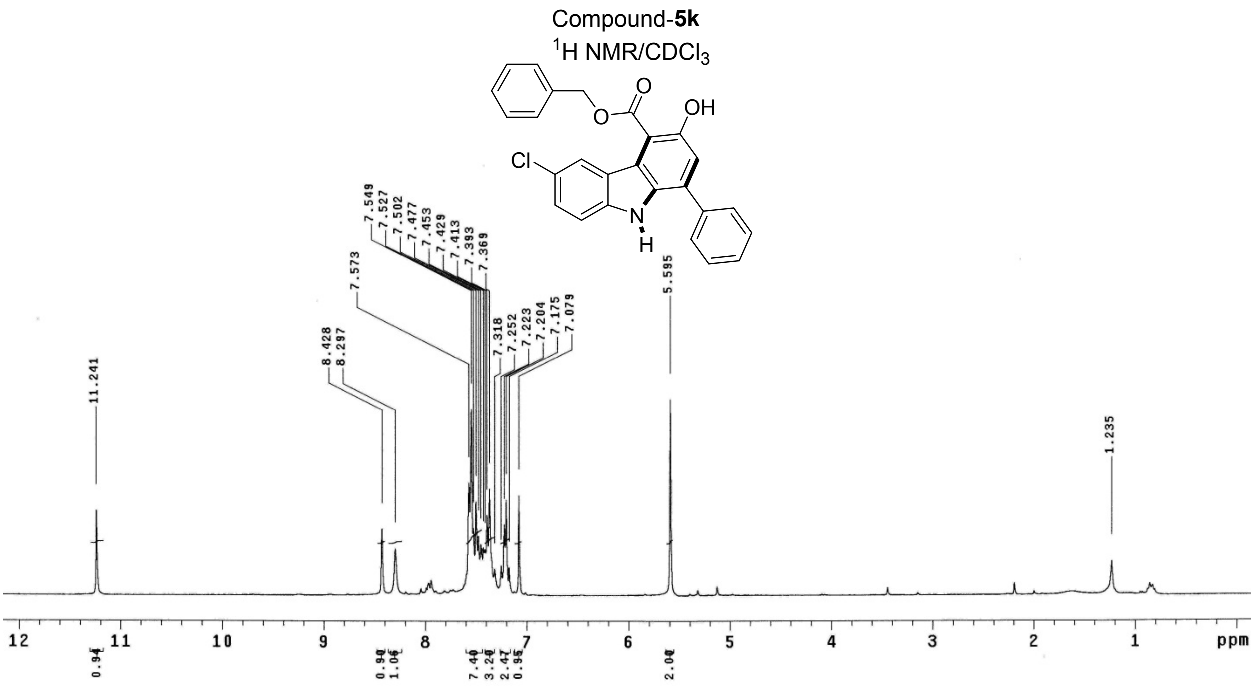
Compound-**5k**
^{1}H NMR/CDCl$_3$
OH
Cl
N
H
11.241
8.428
8.297
7.573
7.549
7.527
7.502
7.477
7.453
7.429
7.413
7.393
7.369
7.318
7.252
7.223
7.204
7.175
7.079
5.595
1.235
ppm

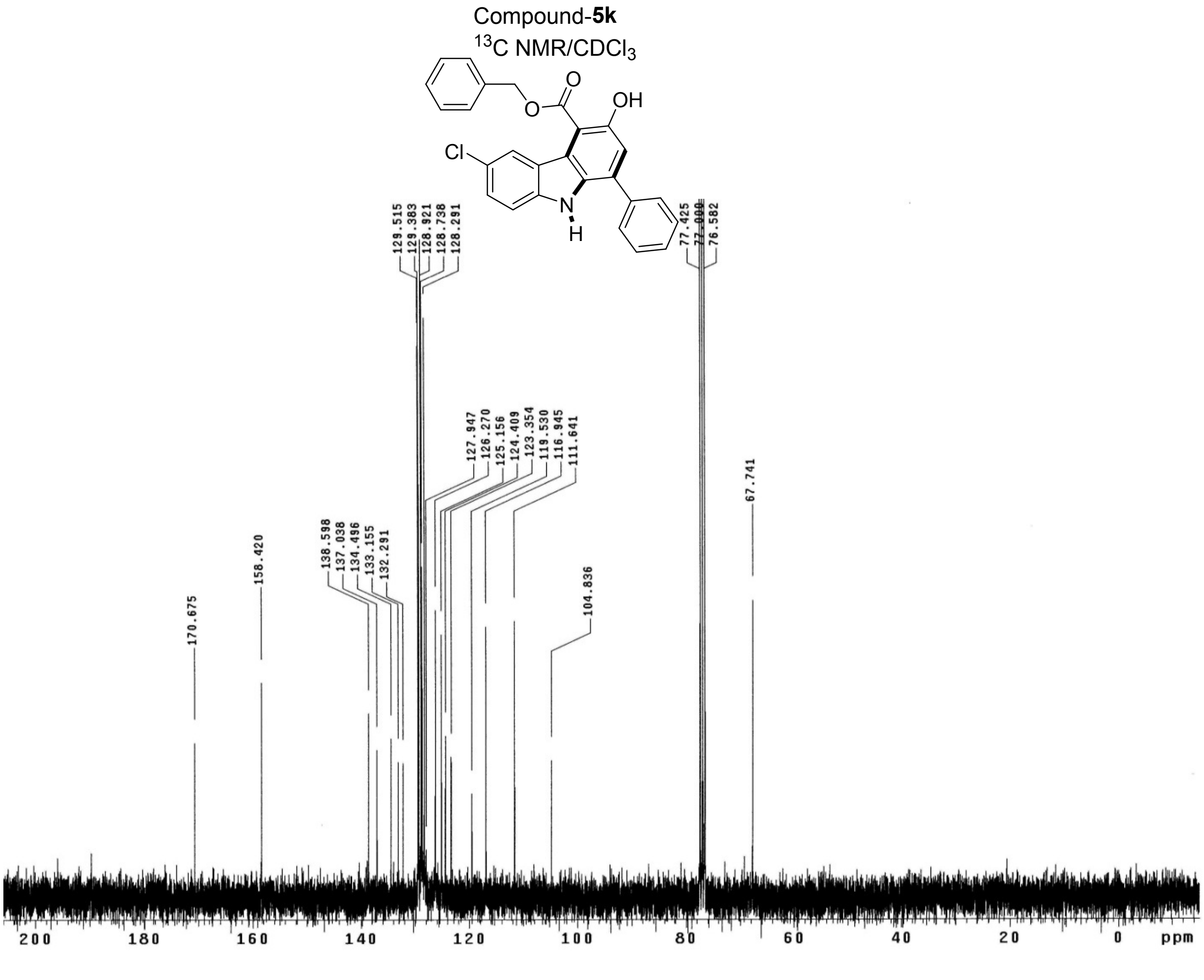
Compound-**5k**
^{13}C NMR/CDCl$_3$
OH
Cl
N
H
170.675
158.420
138.598
137.038
134.496
133.155
132.291
129.515
129.383
128.921
128.738
128.291
127.947
126.270
125.156
124.409
123.354
119.530
116.945
111.641
104.836
77.425
76.582
67.741
ppm

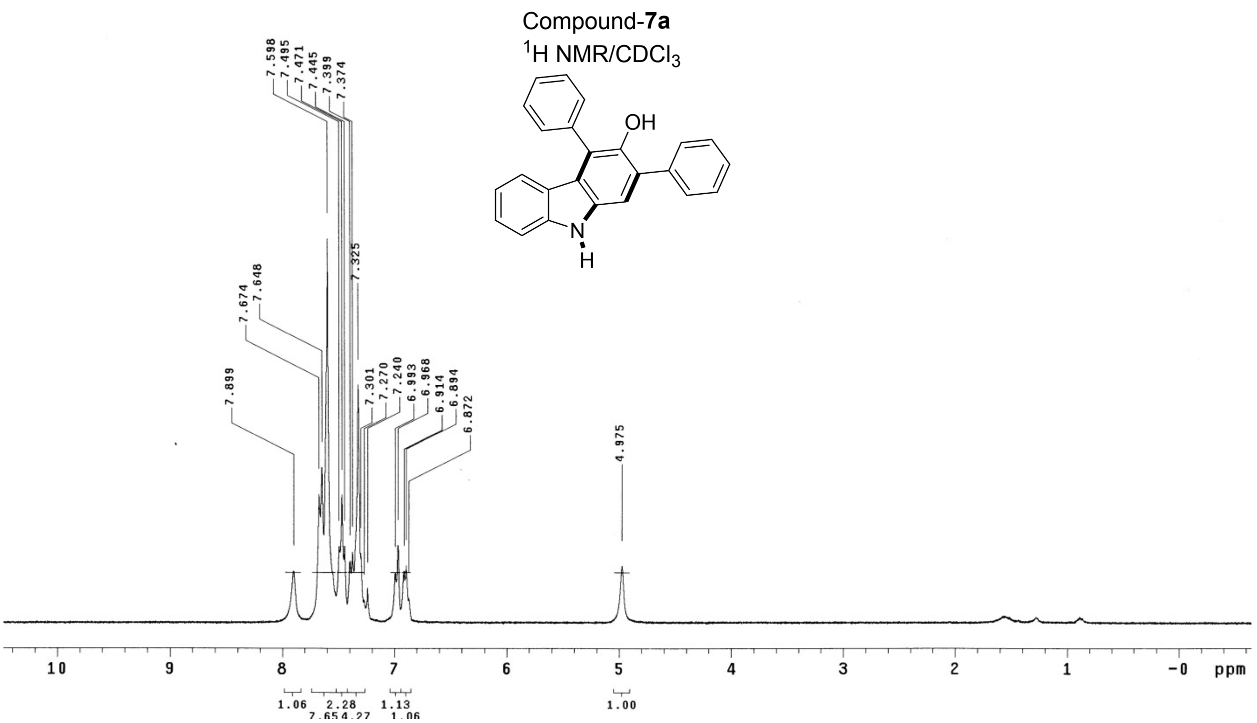
Compound-7a
1H NMR/CDCl3
OH
N
H
7.598
7.495
7.471
7.445
7.399
7.374
7.325
7.674
7.648
7.899
7.301
7.270
7.240
6.993
6.968
6.914
6.894
6.872
4.975
10 9 8 7 6 5 4 3 2 1 -0 ppm
1.06 2.28 1.13 1.00
7.65 4.27 1.06

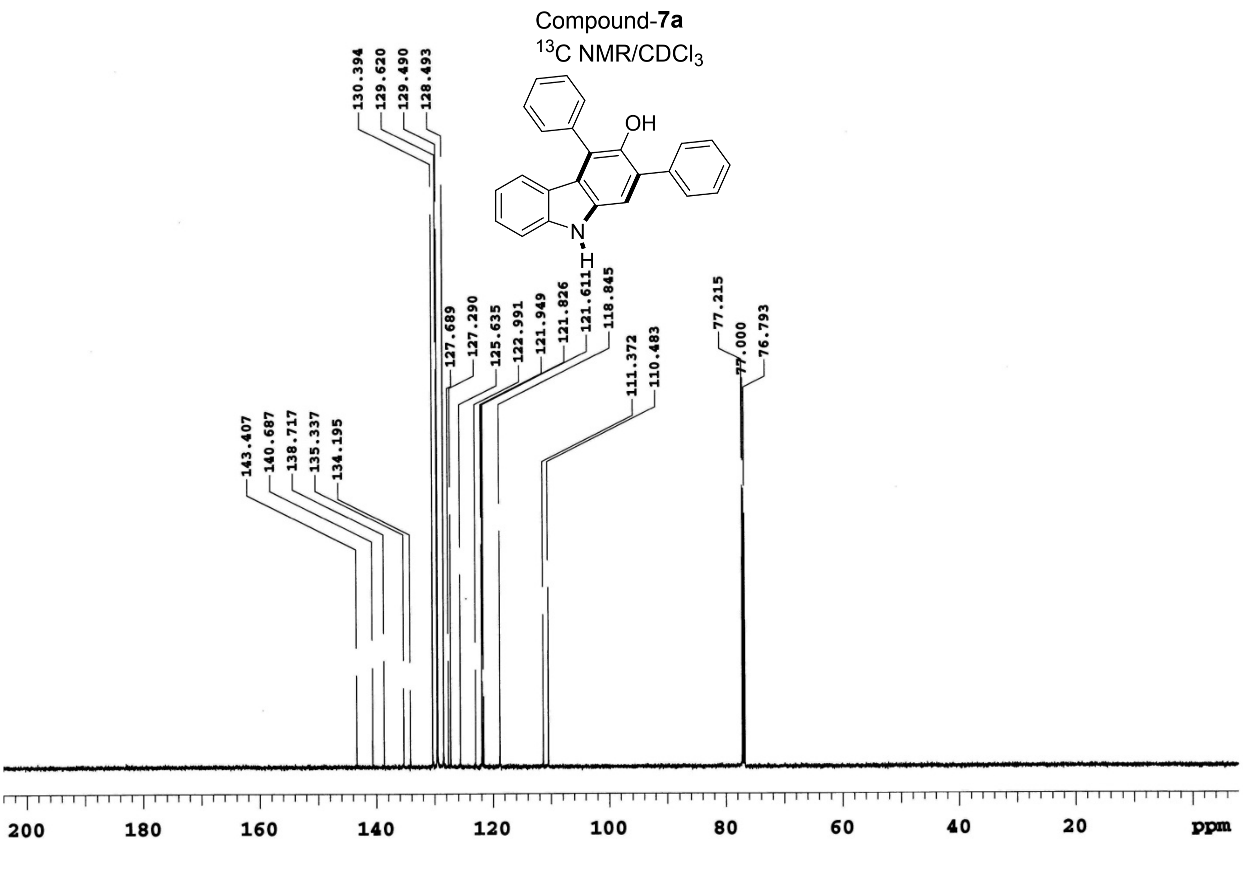
Compound-7a
13C NMR/CDCl3
OH
N
H
130.394
129.620
129.490
128.493
127.689
127.290
125.635
122.991
121.949
121.826
121.611
118.845
111.372
110.483
77.215
77.000
76.793
143.407
140.687
138.717
135.337
134.195
200 180 160 140 120 100 80 60 40 20 ppm

Compound-**7b**
^{1}H NMR/$CDCl_3$ + DMSO-d_6

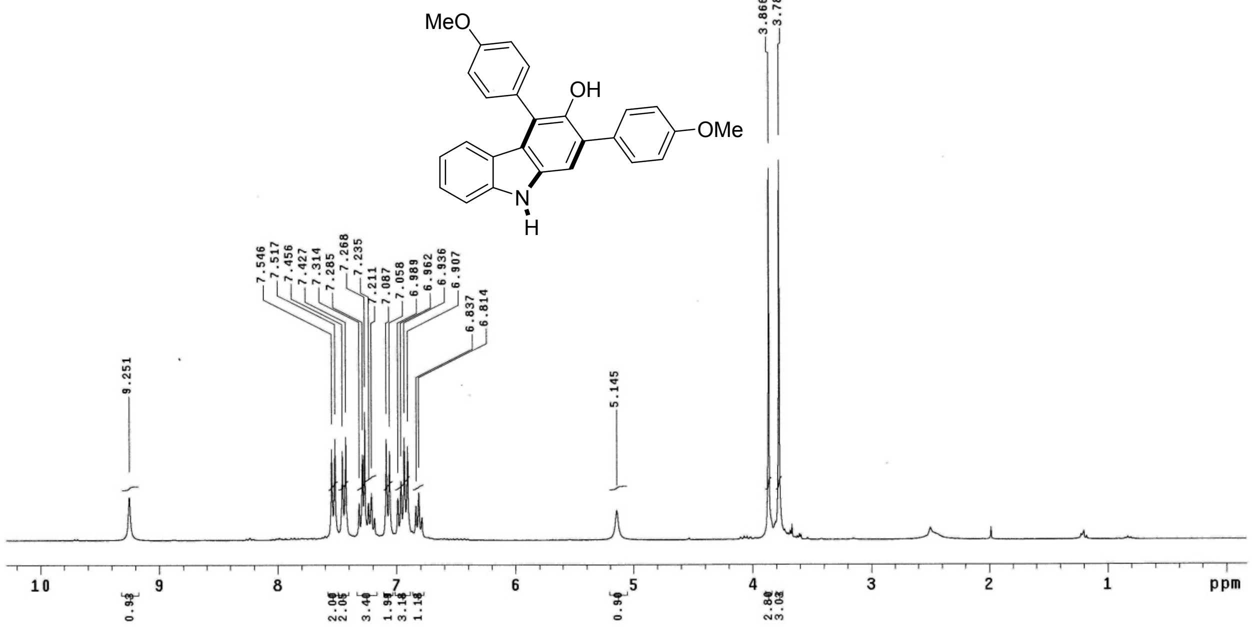

Compound-**7b**
^{13}C NMR/$CDCl_3$ + DMSO-d_6

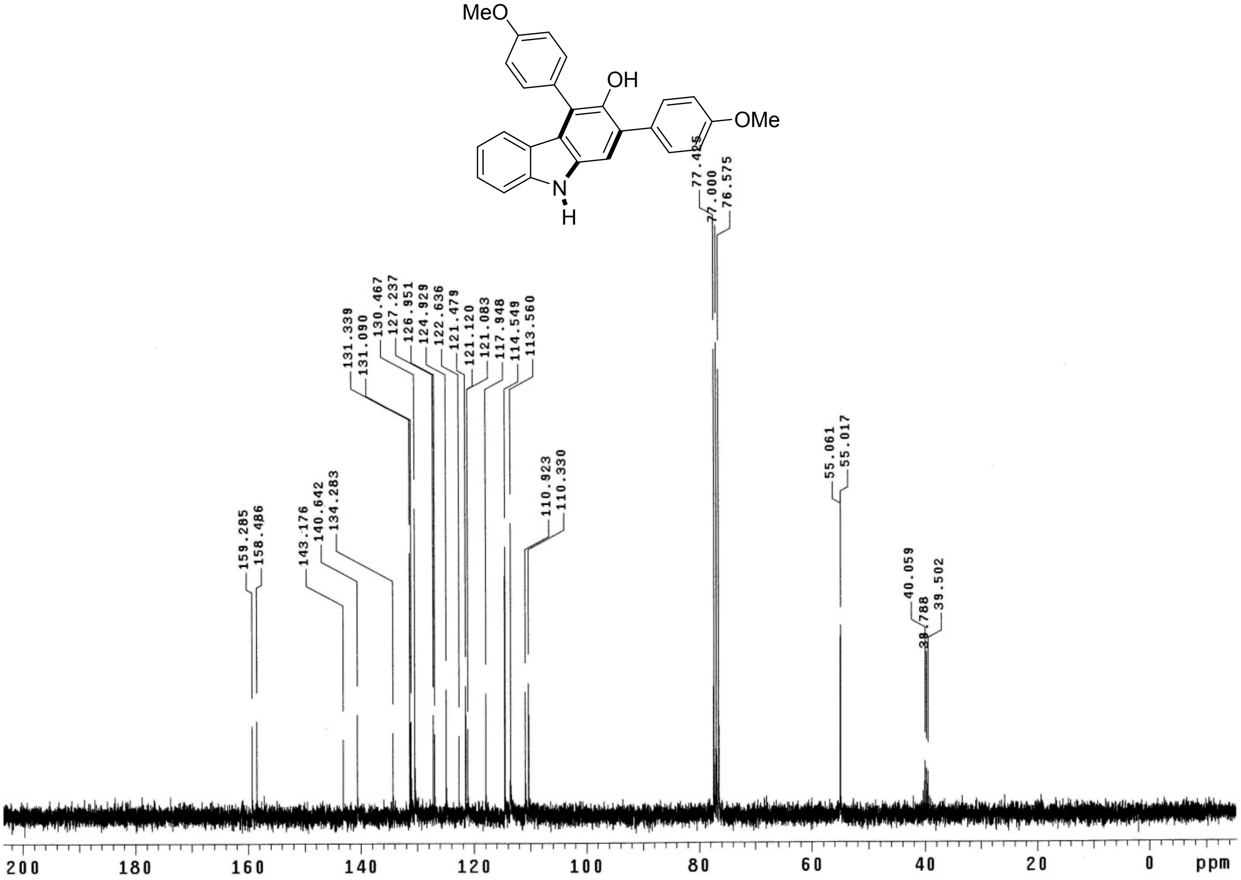

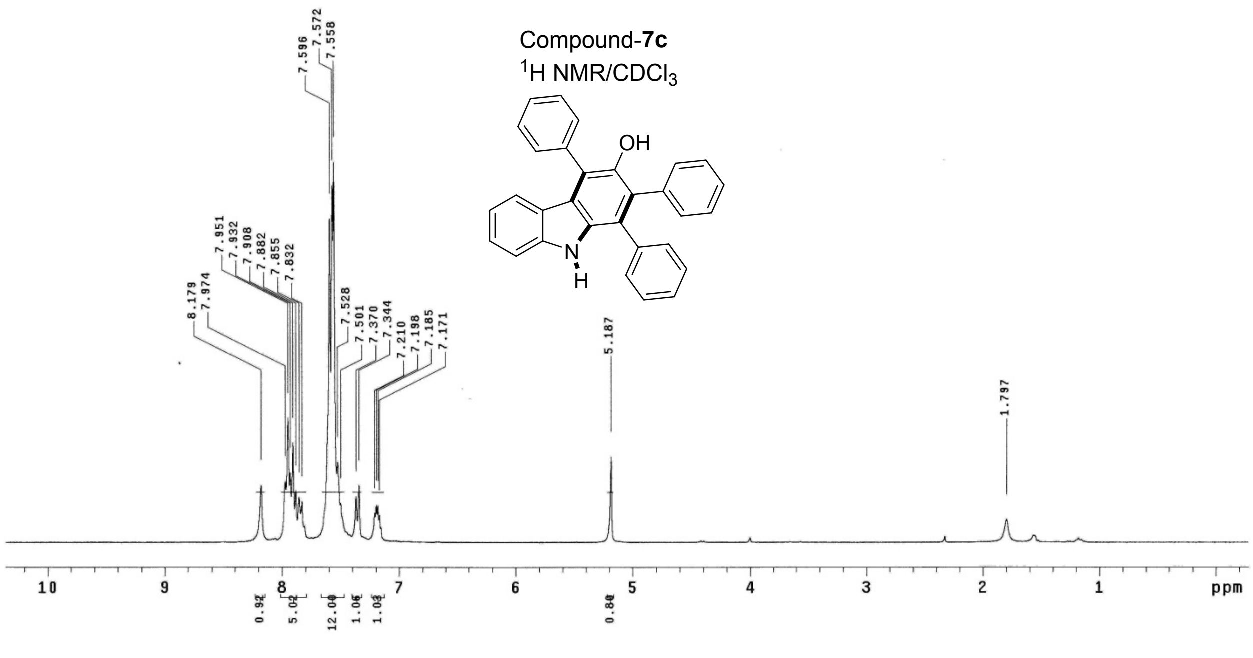
Compound-7c
^{1}H NMR/CDCl$_3$
OH
N
H
8.179
7.974
7.951
7.932
7.908
7.882
7.855
7.832
7.596
7.572
7.558
7.528
7.501
7.370
7.344
7.210
7.198
7.185
7.171
5.187
1.797
ppm

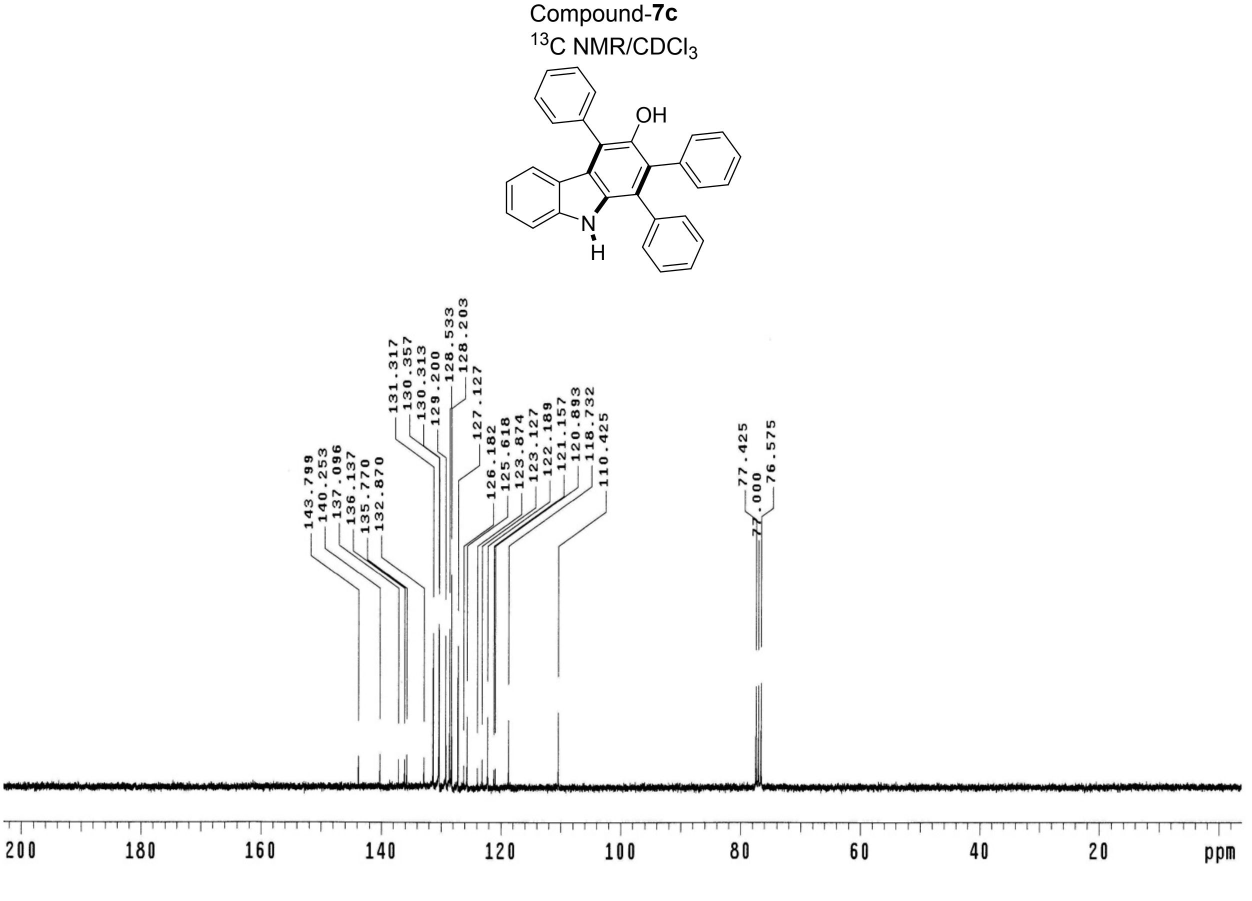
Compound-7c
^{13}C NMR/CDCl$_3$
OH
N
H
143.799
140.253
137.096
136.137
135.770
132.870
131.317
130.357
130.313
129.200
128.533
128.203
127.127
126.182
125.618
123.874
123.127
122.189
121.157
120.893
118.732
110.425
77.425
77.000
76.575
ppm

Compound-**7d**
^{1}H NMR/$CDCl_3$

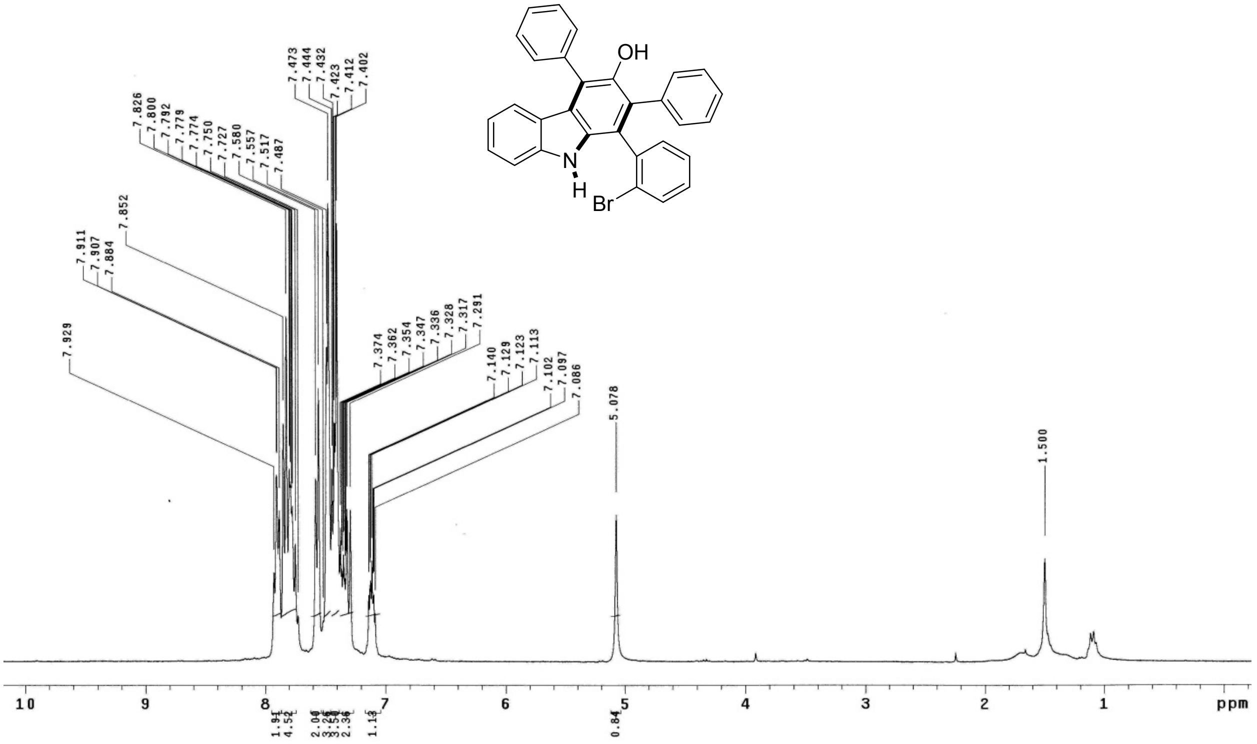

Compound-**7d**
^{13}C NMR/$CDCl_3$

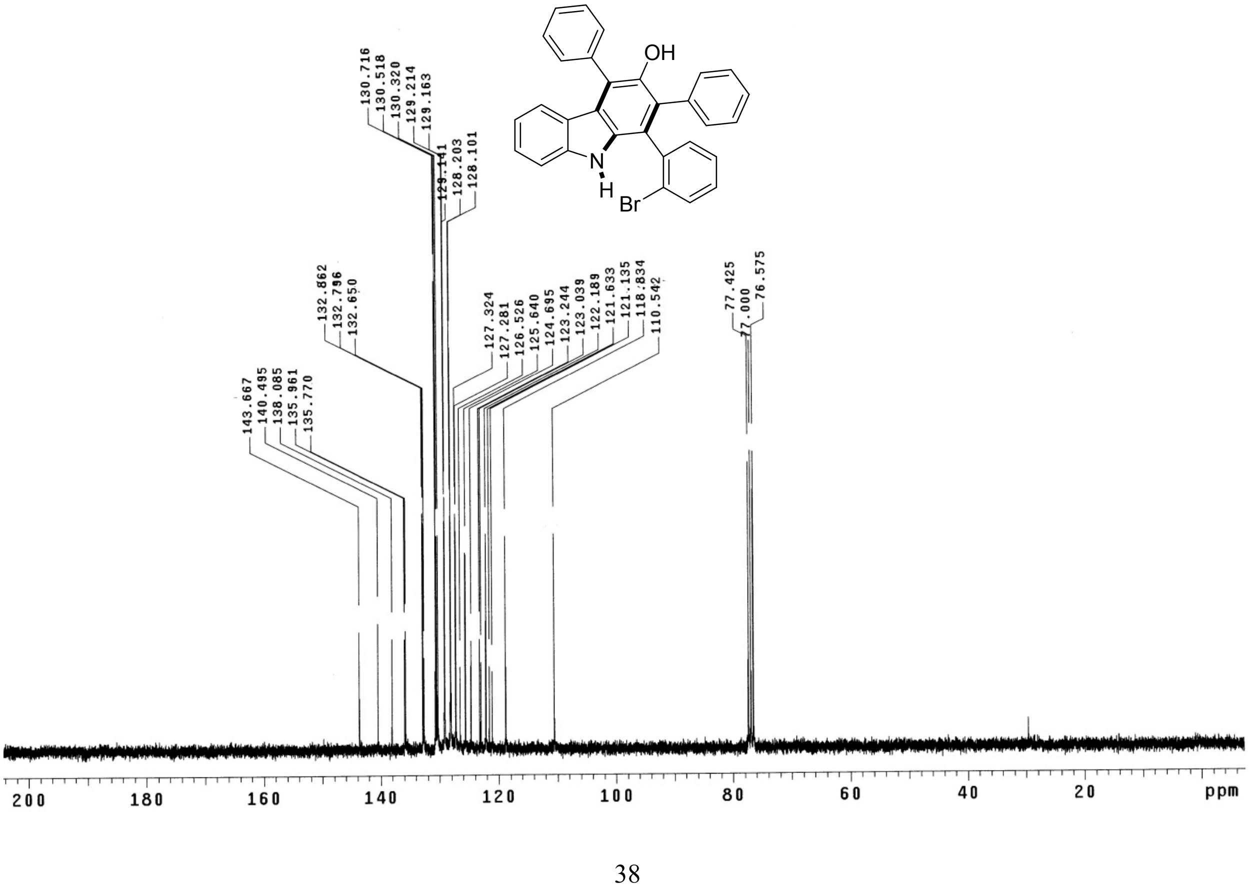

Compound-**7e**
^{1}H NMR/CDCl$_3$

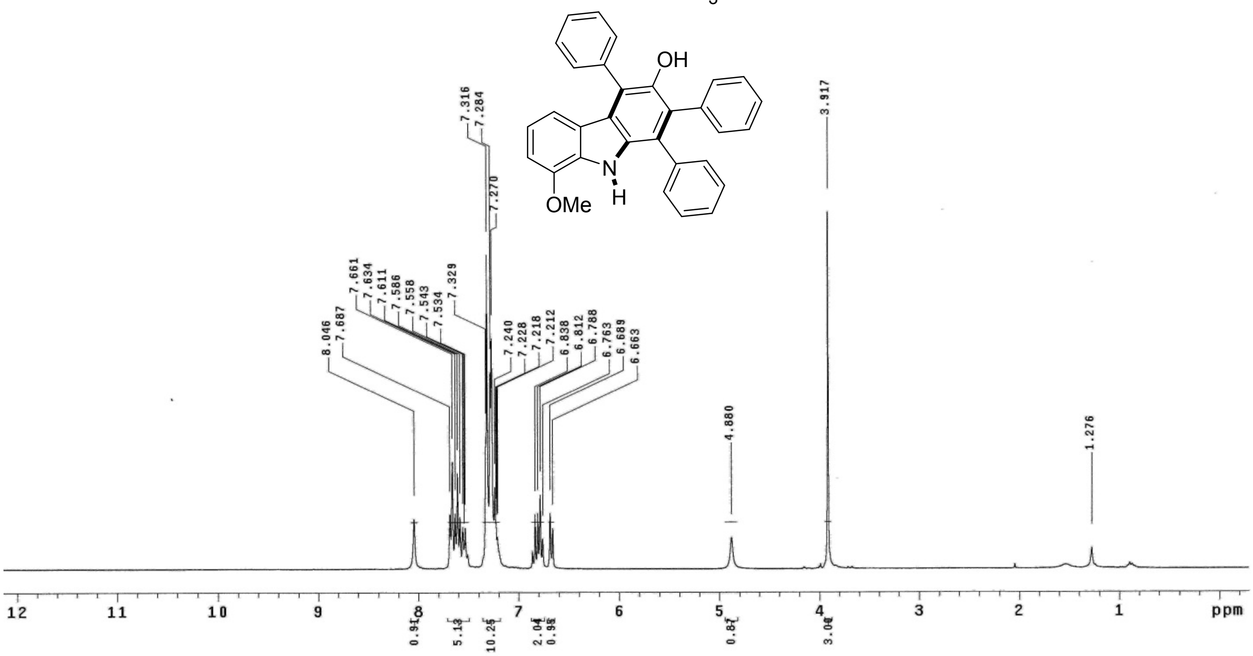

Compound-**7e**
^{13}C NMR/CDCl$_3$

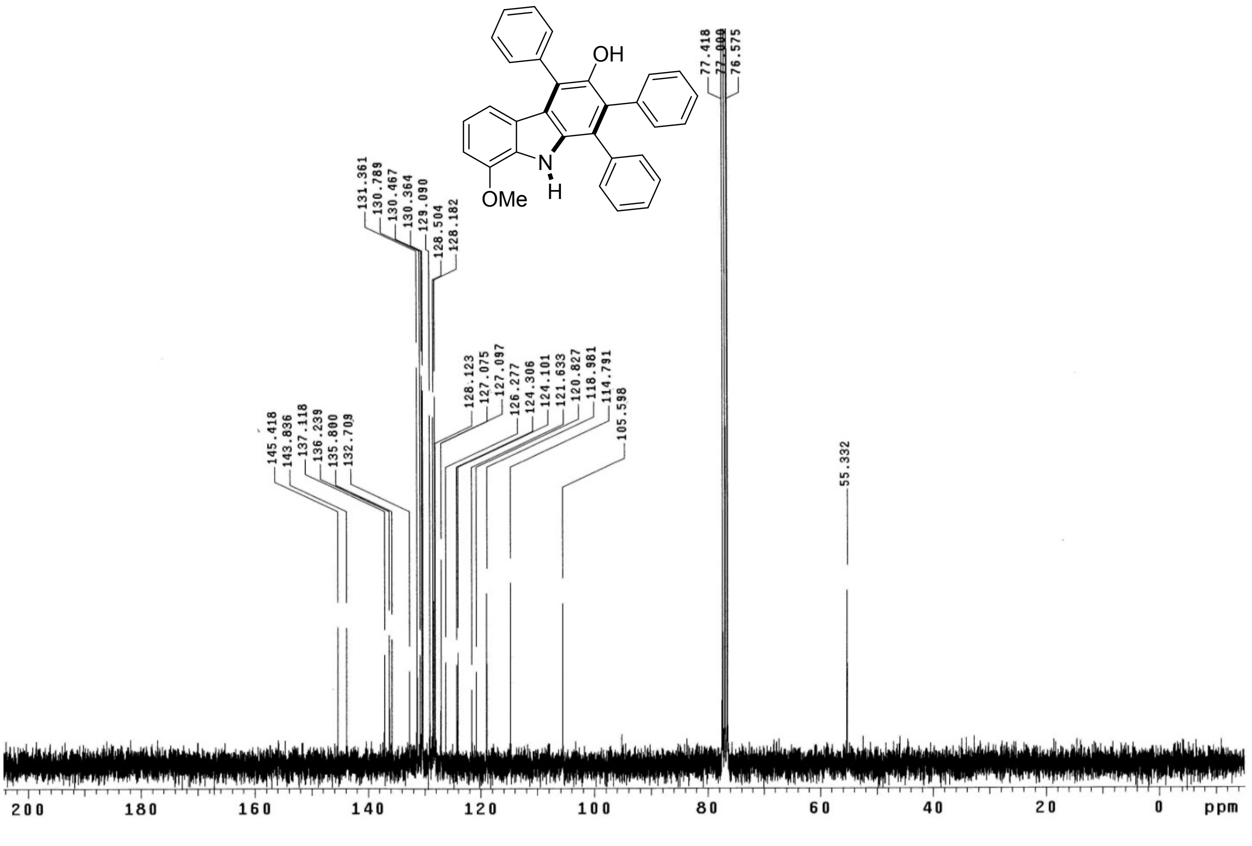

Compound-**7f**
^{1}H NMR/$CDCl_3$

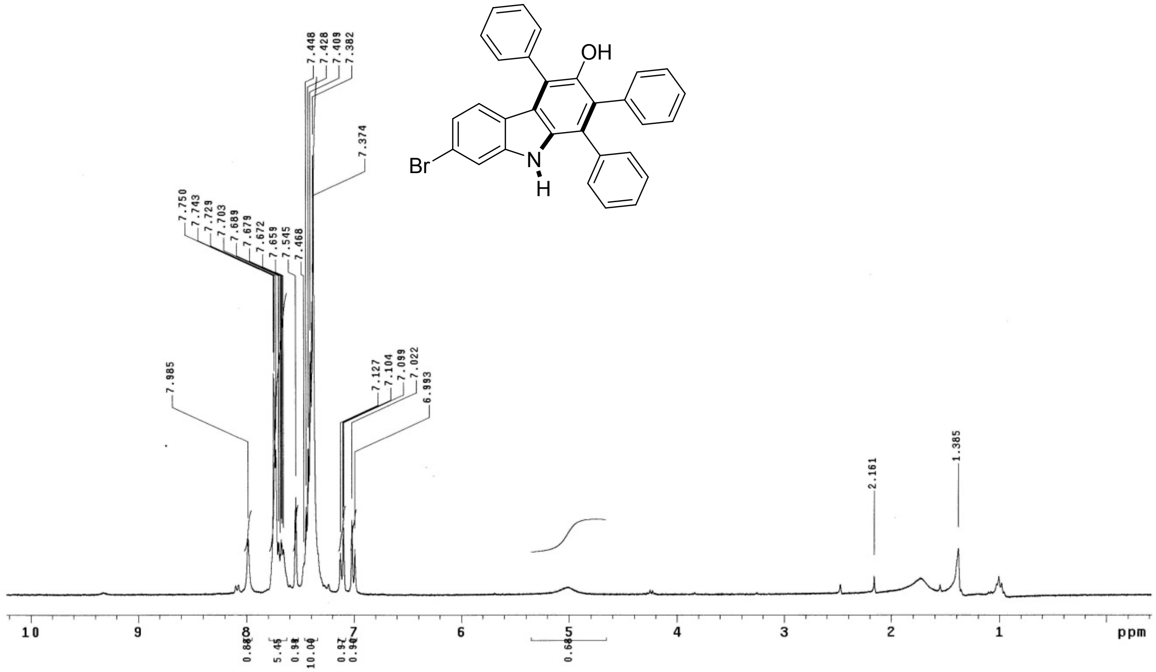

Compound-**7f**
^{13}C NMR/$CDCl_3$

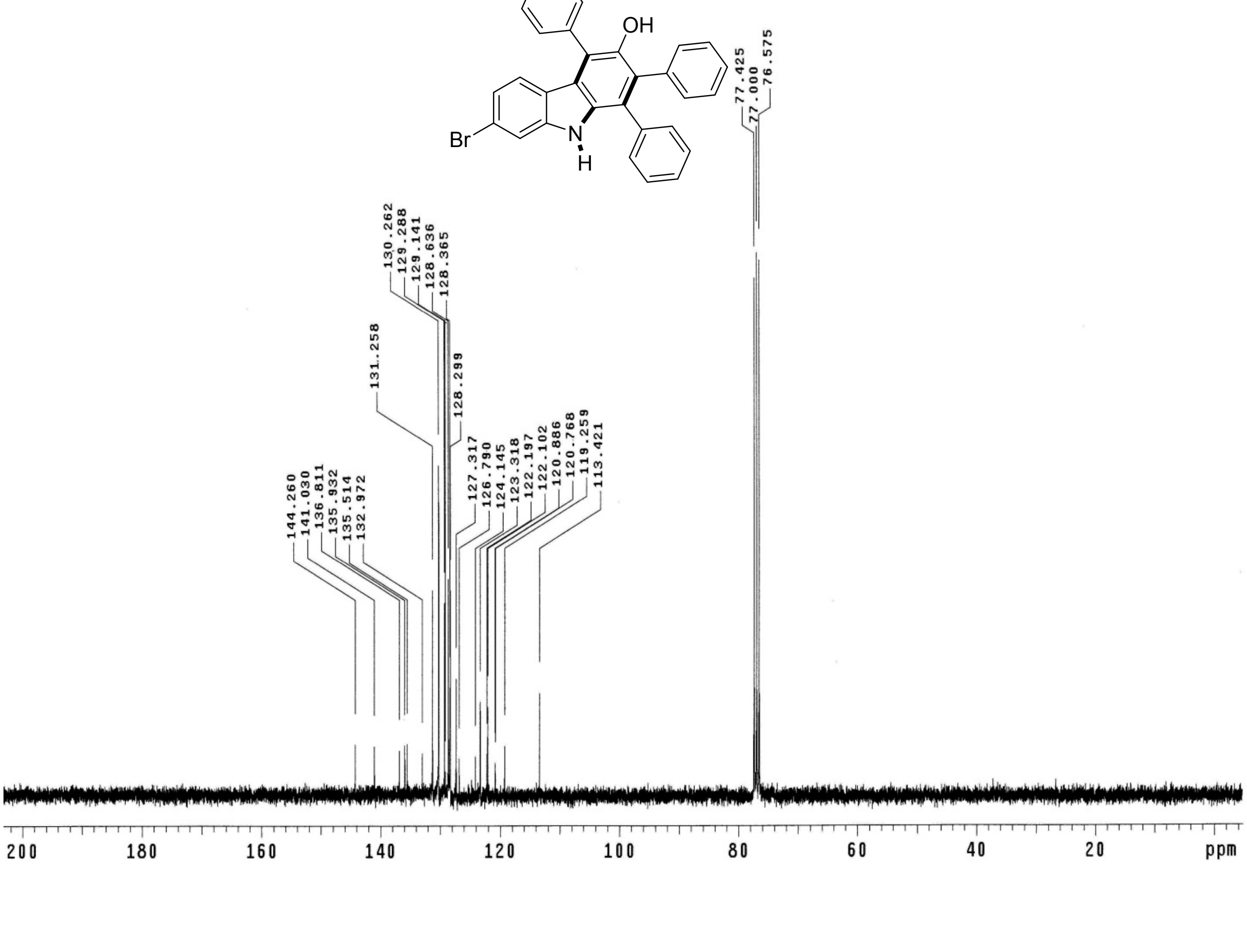

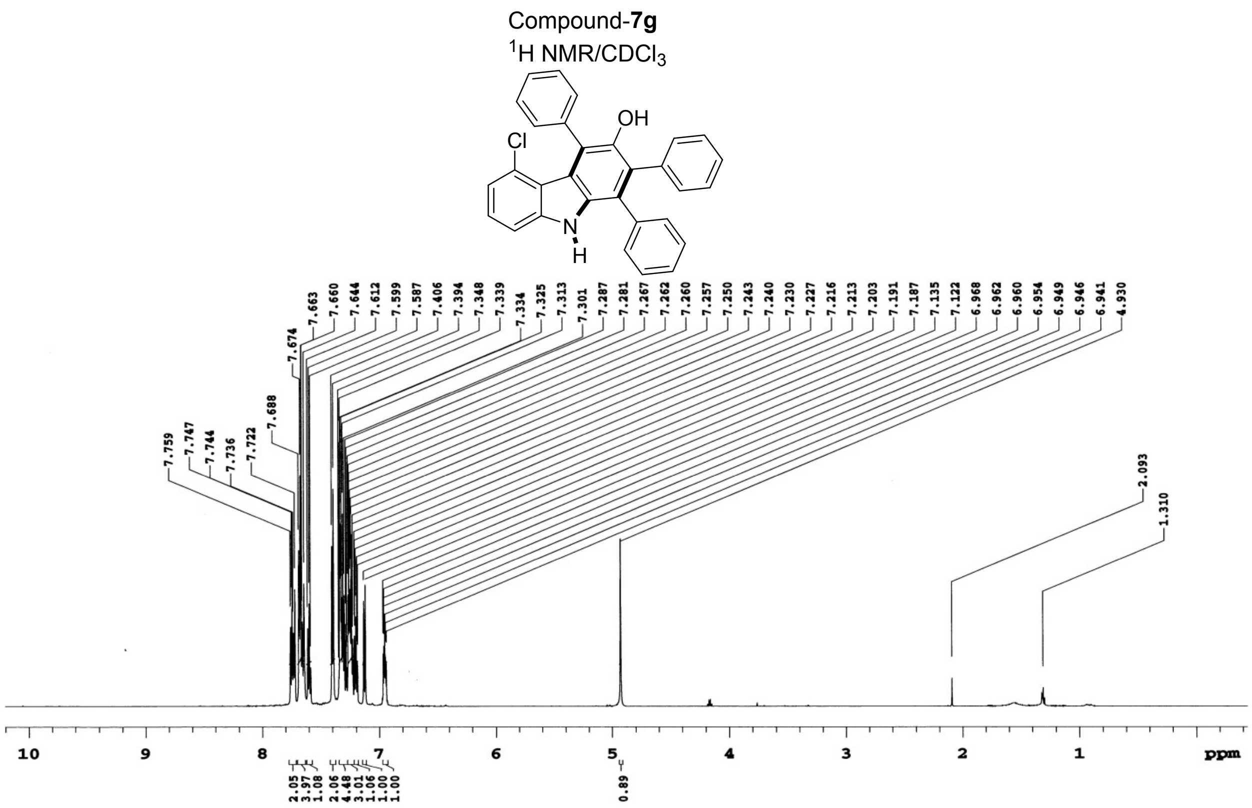

Compound-7g
1H NMR/CDCl3
OH
Cl
N
H
7.759
7.747
7.744
7.736
7.722
7.688
7.674
7.663
7.660
7.644
7.612
7.599
7.587
7.406
7.394
7.348
7.339
7.334
7.325
7.313
7.301
7.287
7.281
7.267
7.262
7.260
7.257
7.250
7.243
7.240
7.230
7.227
7.216
7.213
7.203
7.191
7.187
7.135
7.122
6.968
6.962
6.960
6.954
6.949
6.946
6.941
4.930
2.093
1.310
10
9
8
7
6
5
4
3
2
1
ppm
2.05
3.97
1.08
2.06
4.48
3.01
1.06
1.00
1.00
0.89

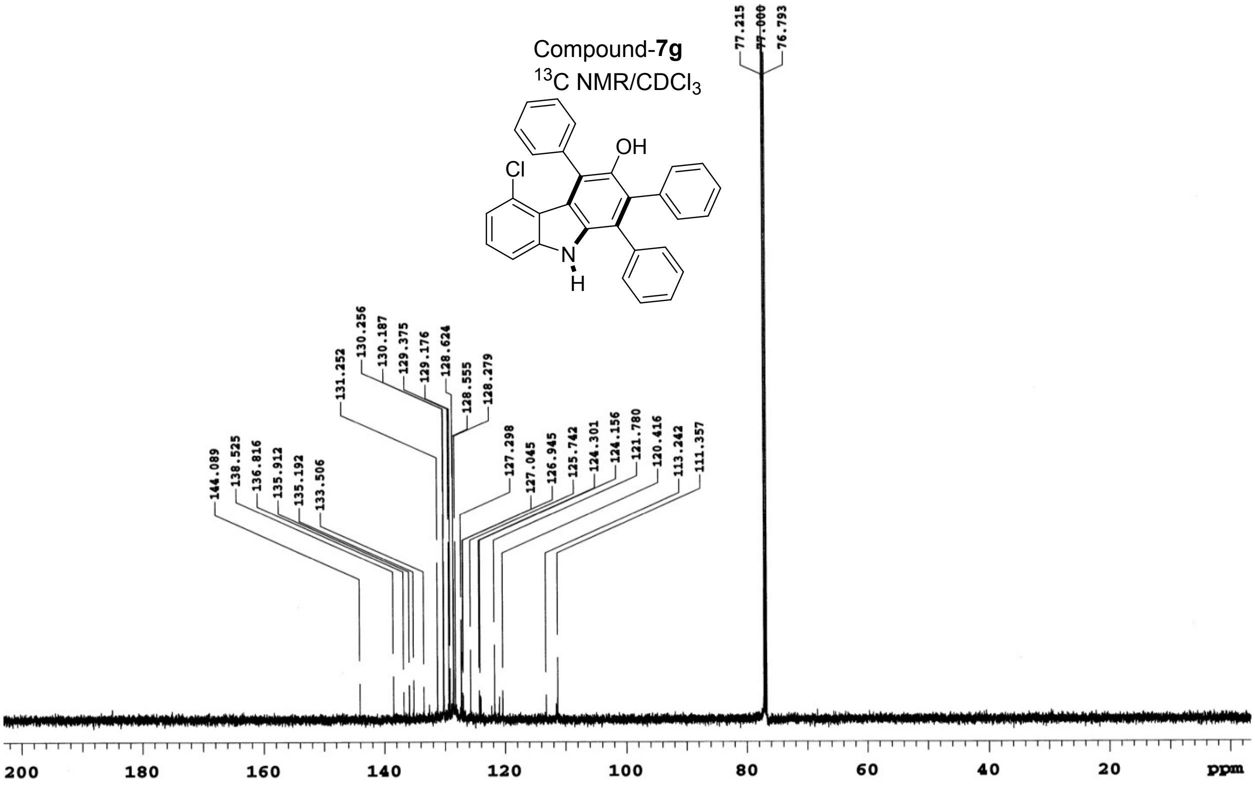

Compound-7g
13C NMR/CDCl3
OH
Cl
N
H
77.215
77.000
76.793
144.089
138.525
136.816
135.912
135.192
133.506
131.252
130.256
130.187
129.375
129.176
128.624
128.555
128.279
127.298
127.045
126.945
125.742
124.301
124.156
121.780
120.416
113.242
111.357
200
180
160
140
120
100
80
60
40
20
ppm

Compound-**9a**
^{1}H NMR/$CDCl_3$

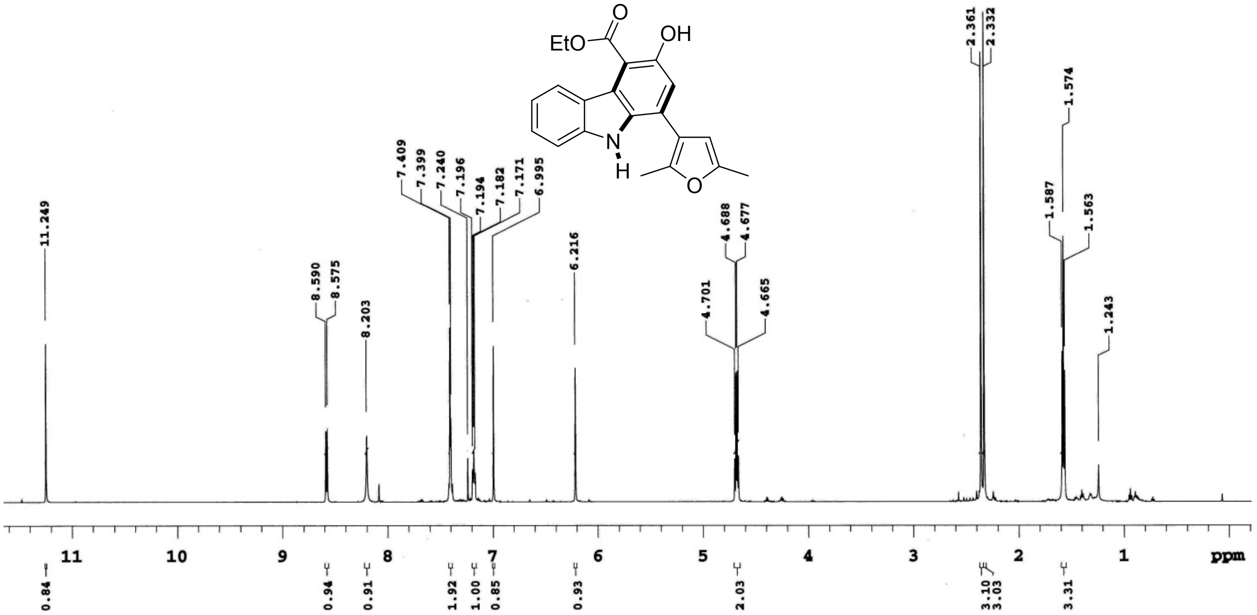

Compound-**9a**
^{13}C NMR/$CDCl_3$

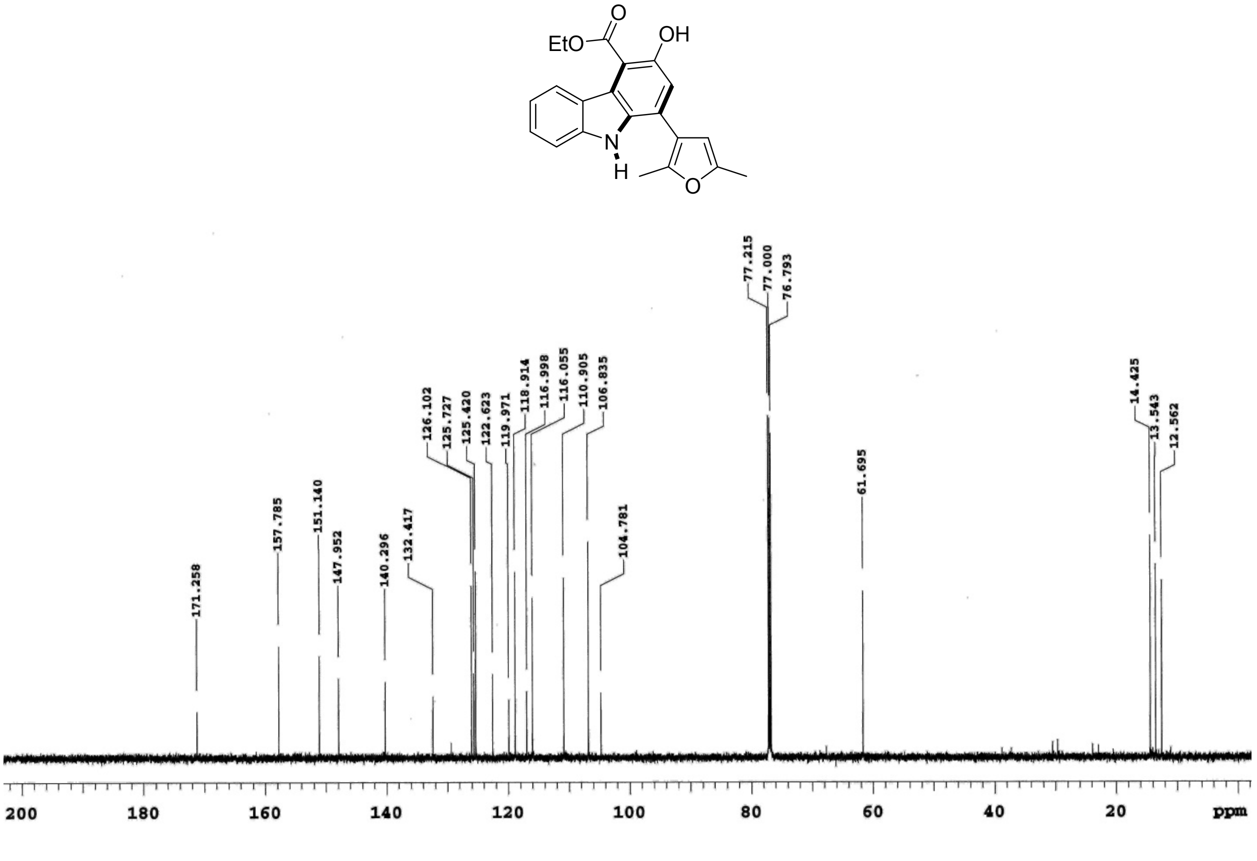

Compound-**9b**
^{1}H NMR/$CDCl_3$

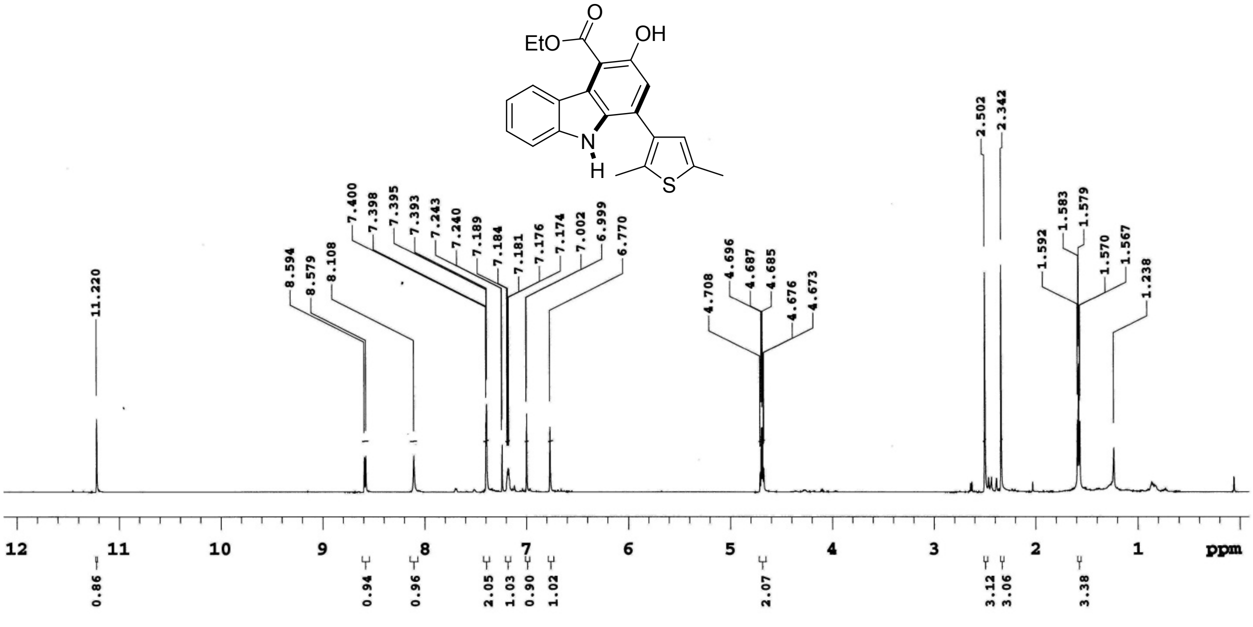

Compound-**9b**
^{13}C NMR/$CDCl_3$

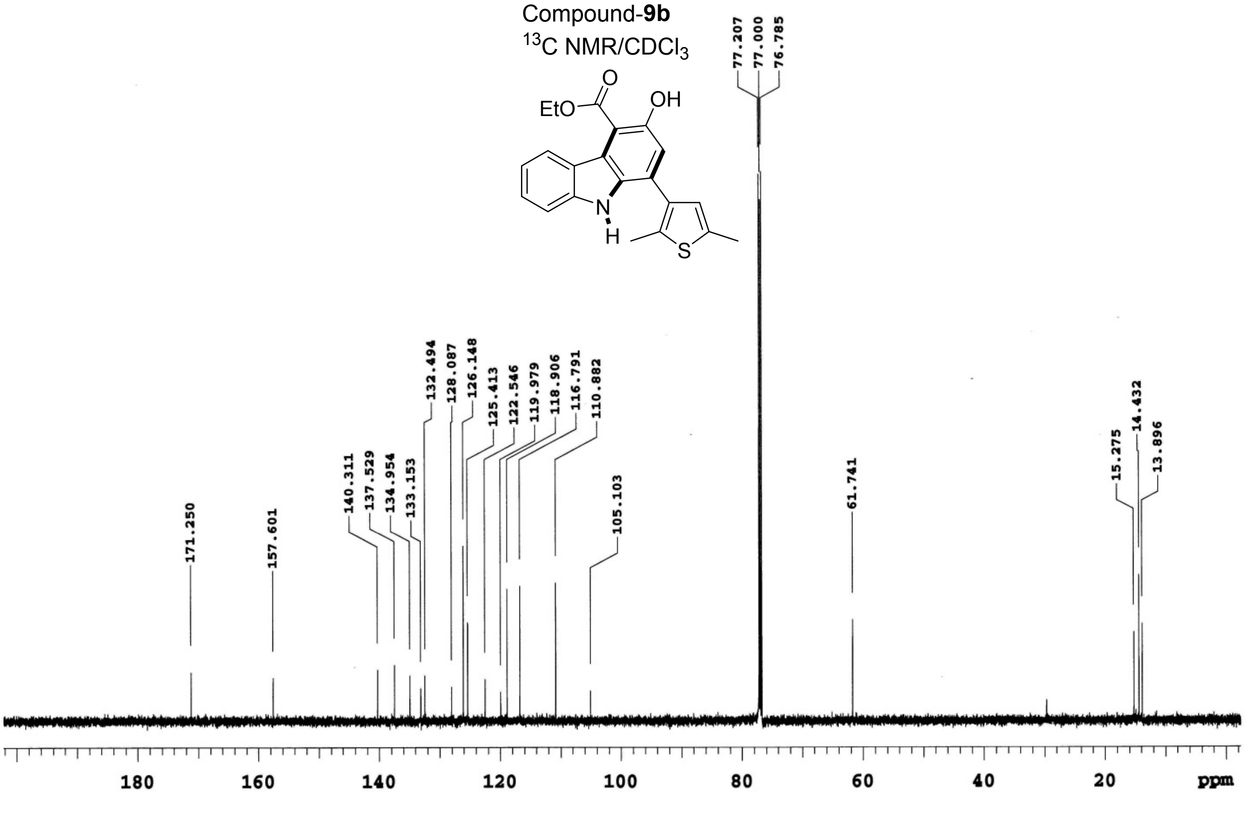

Compound-**9c**
^1H NMR/$CDCl_3$

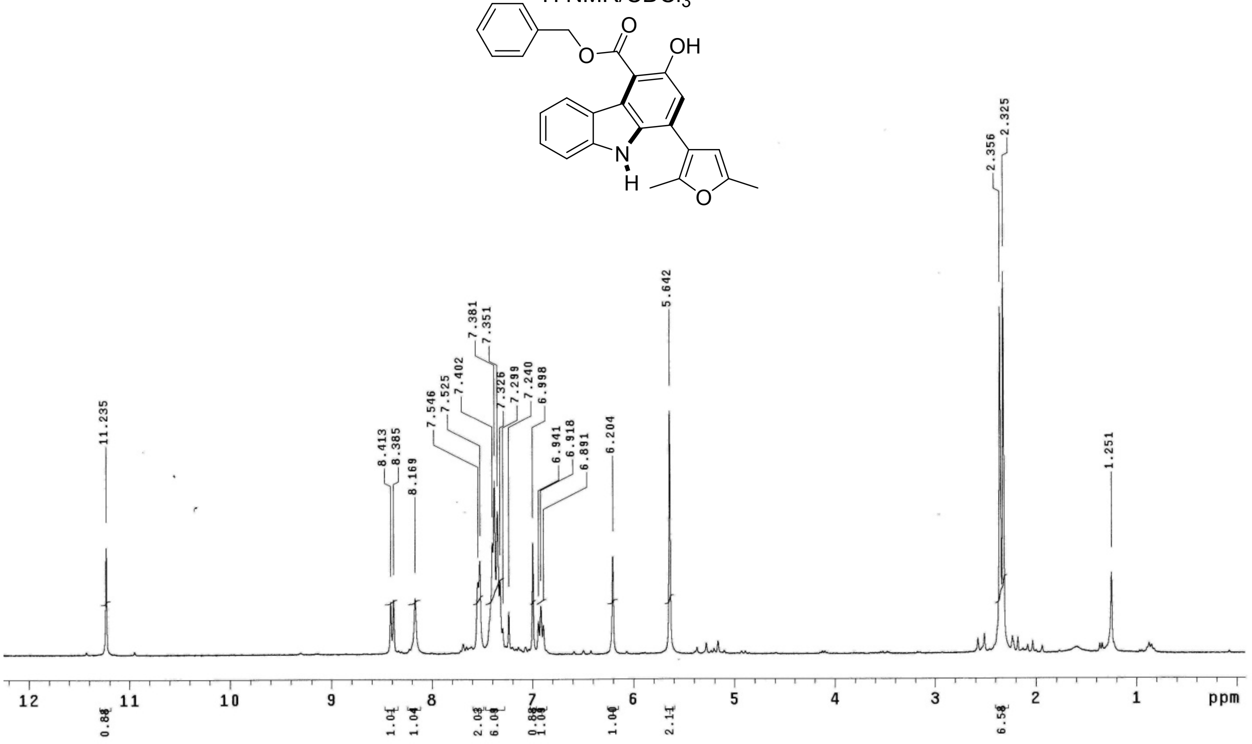

Compound-**9c**
^{13}C NMR/$CDCl_3$

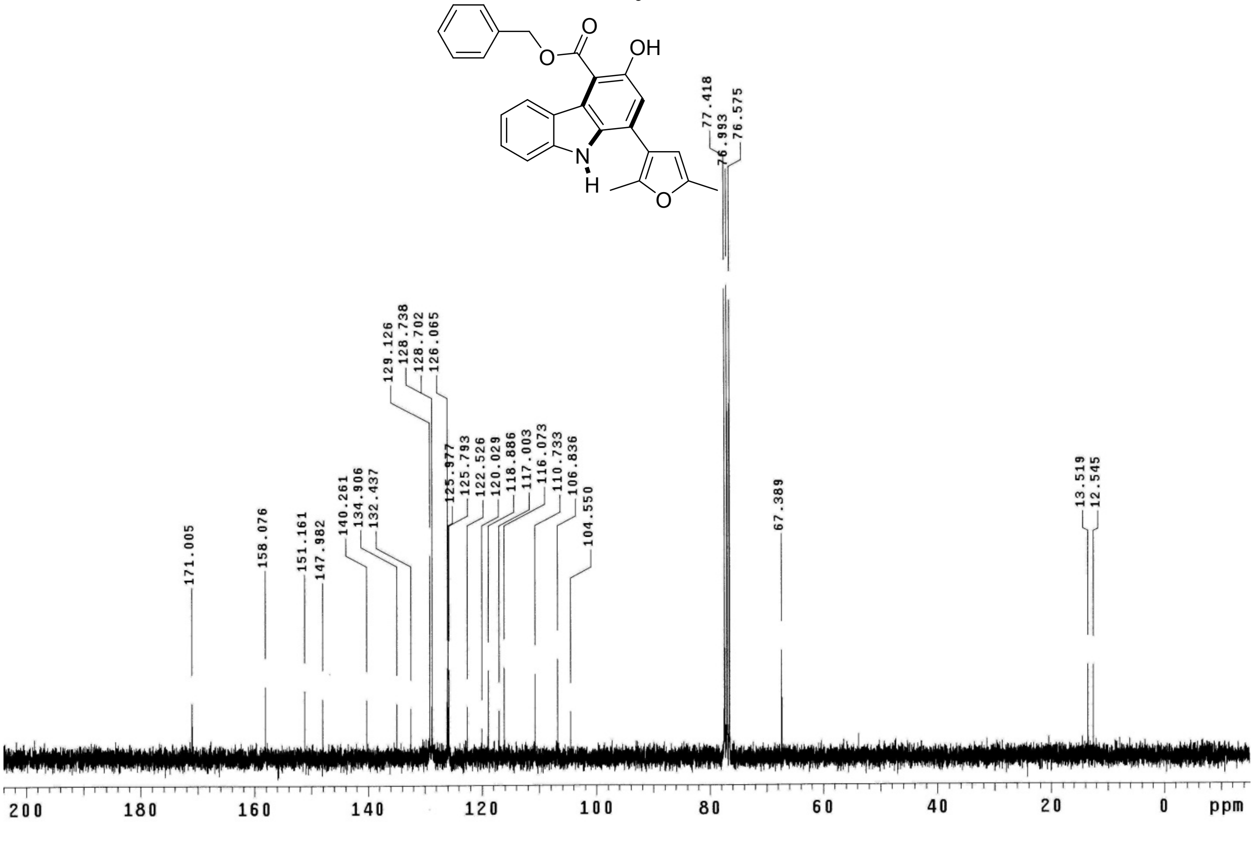

Compound-**9d**
^{1}H NMR/$CDCl_3$

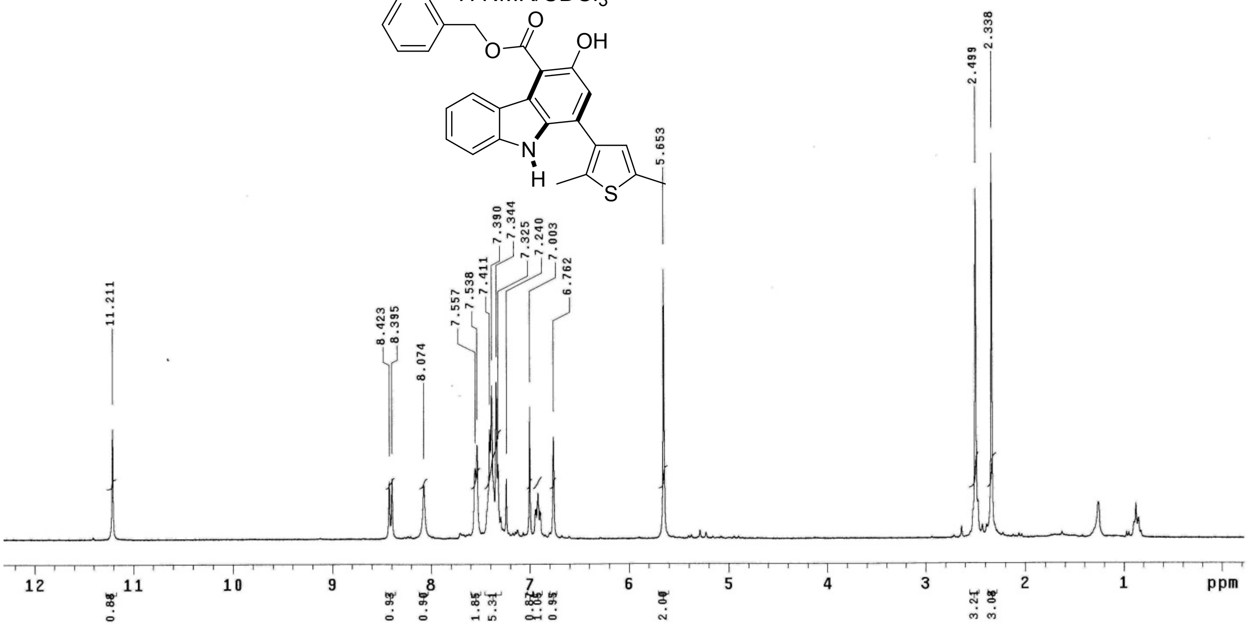

Compound-**9d**
^{13}C NMR/$CDCl_3$

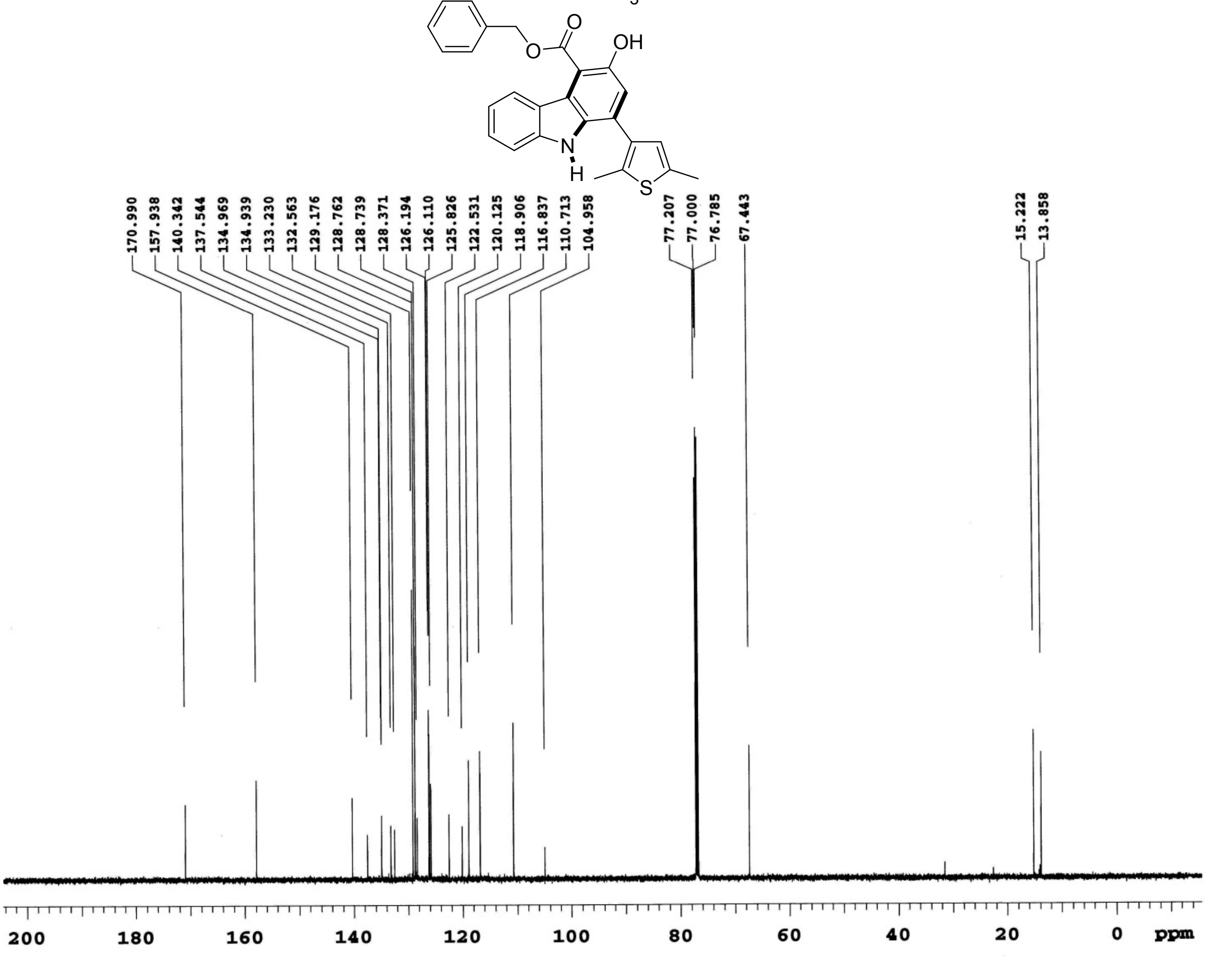

Compound-**9e**
^1H NMR/$CDCl_3$

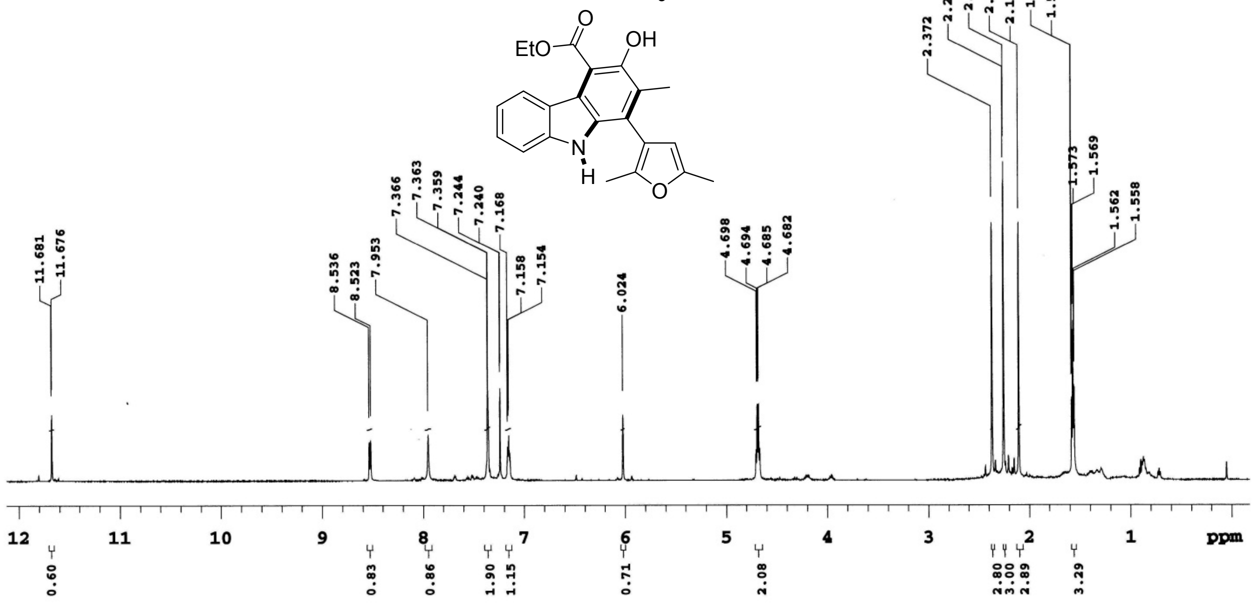

Compound-**9e**
^{13}C NMR/$CDCl_3$

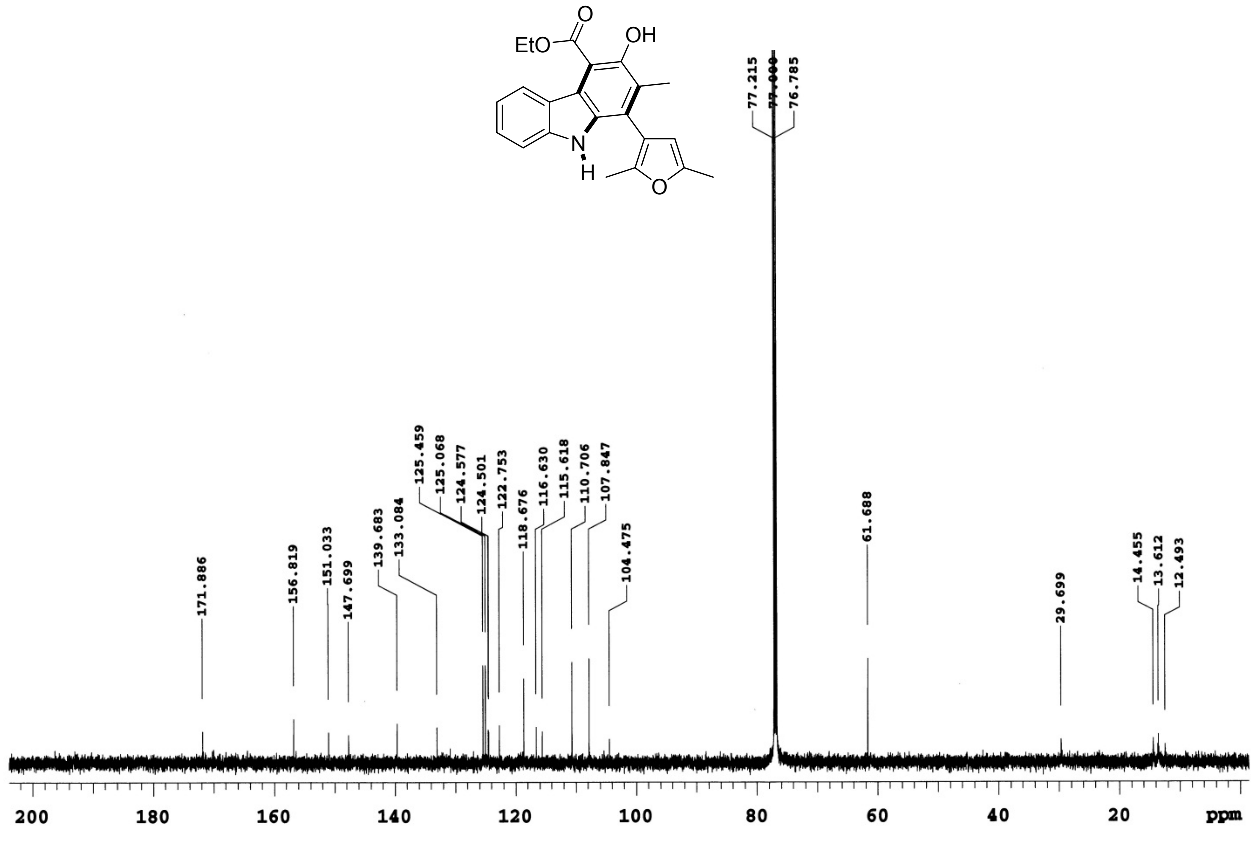

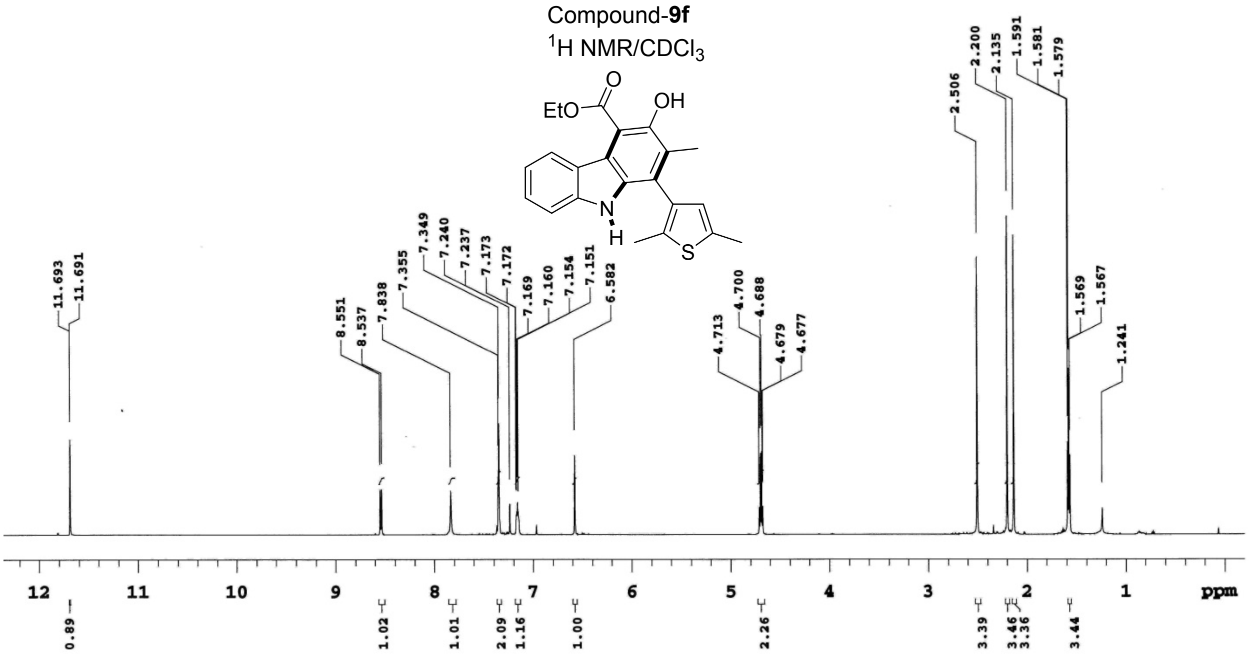
Compound-9f
1H NMR/CDCl3
EtO
O
OH
N
H
S
11.693
11.691
8.551
8.537
7.838
7.355
7.349
7.240
7.237
7.173
7.172
7.169
7.160
7.154
7.151
6.582
4.713
4.700
4.688
4.679
4.677
2.506
2.200
2.135
1.591
1.581
1.579
1.569
1.567
1.241
0.89
1.02
1.01
2.09
1.16
1.00
2.26
3.39
3.46
3.36
3.44
ppm

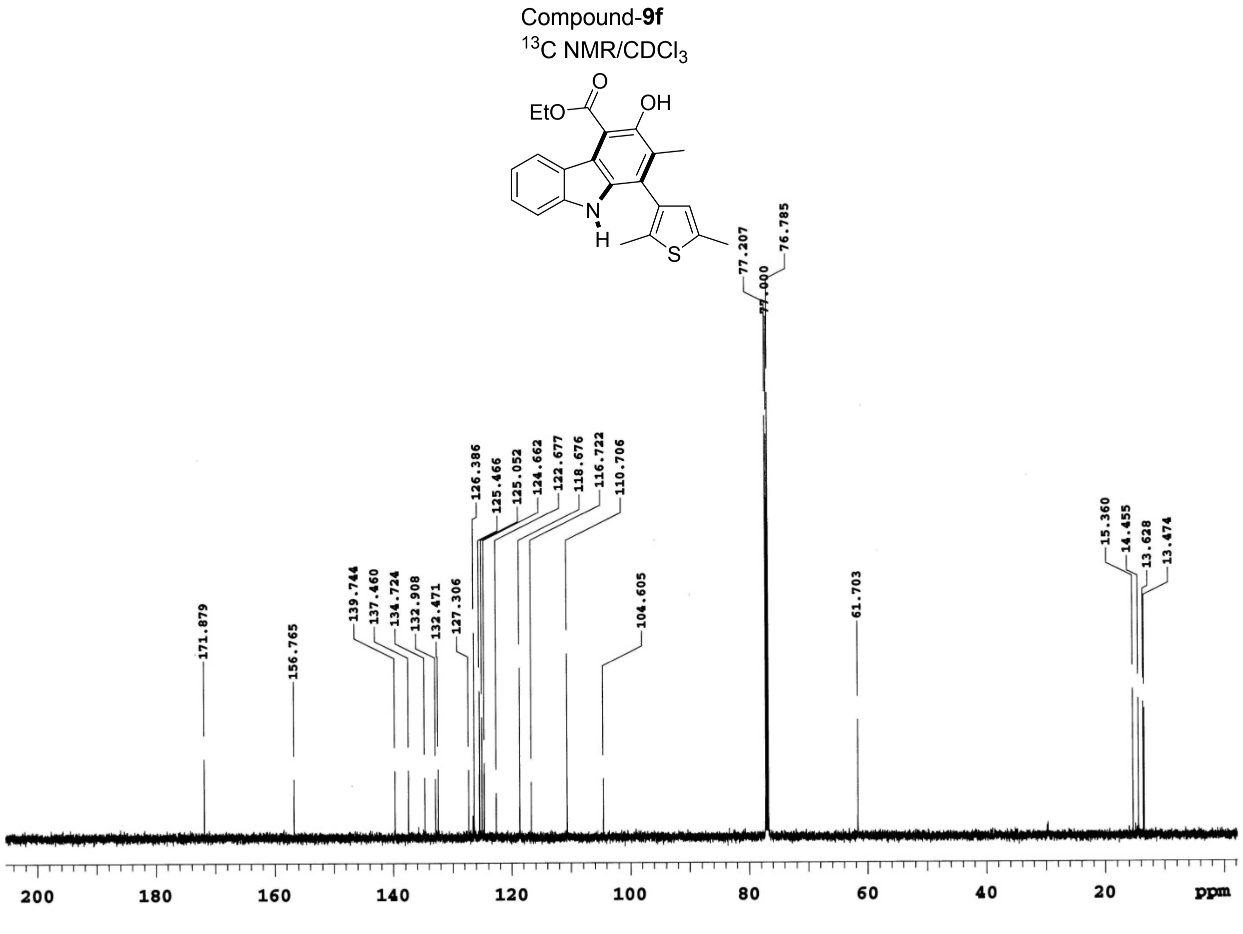
Compound-9f
13C NMR/CDCl3
EtO
O
OH
N
H
S
171.879
156.765
139.744
137.460
134.724
132.908
132.471
127.306
126.386
125.466
125.052
124.662
122.677
118.676
116.722
110.706
104.605
77.207
77.000
76.785
61.703
15.360
14.455
13.628
13.474
ppm

Compound-**20**

^1H NMR/$CDCl_3$

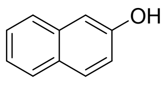

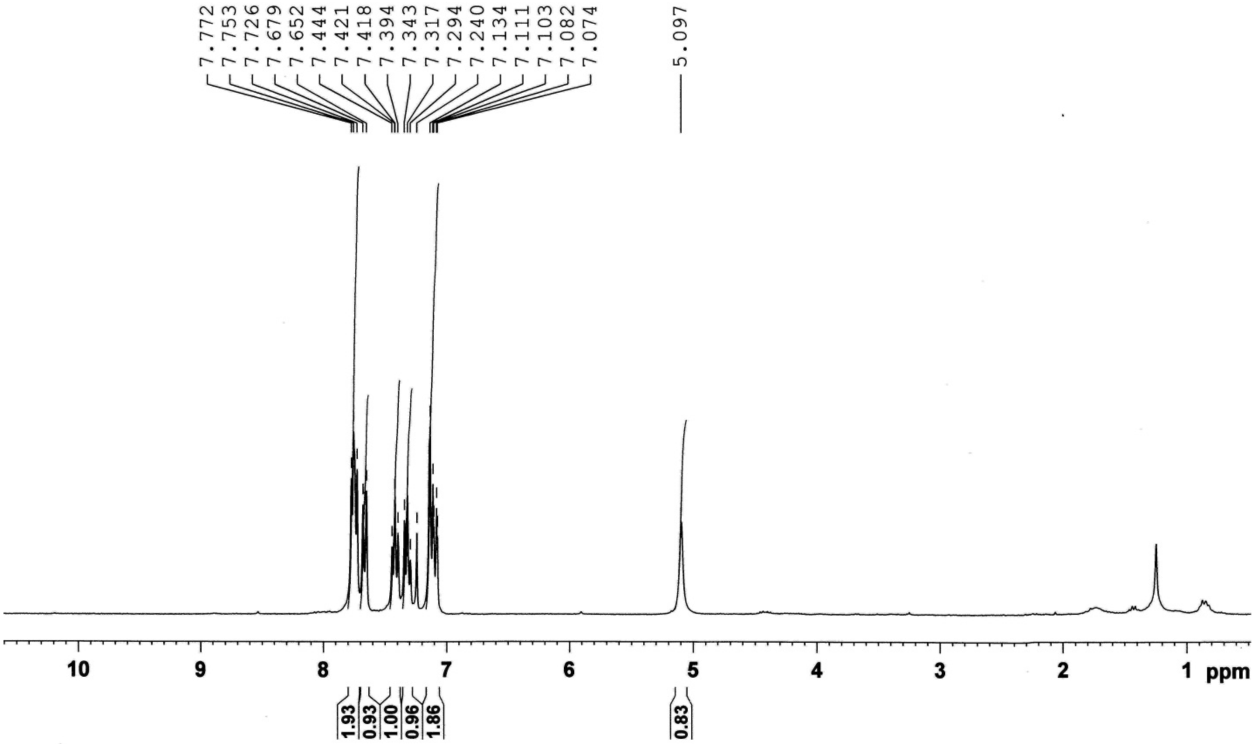

Compound-**21a**
^{1}H NMR/CDCl$_3$

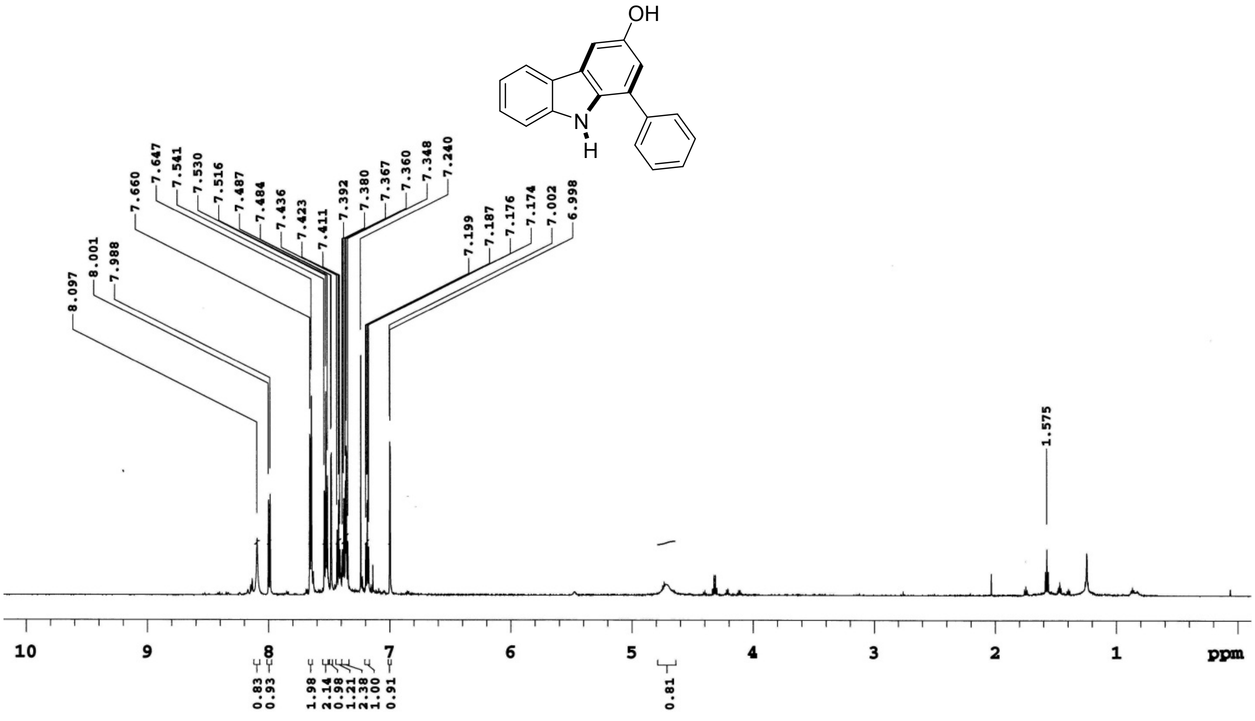

Compound-**21a**
^{13}C NMR/CDCl$_3$

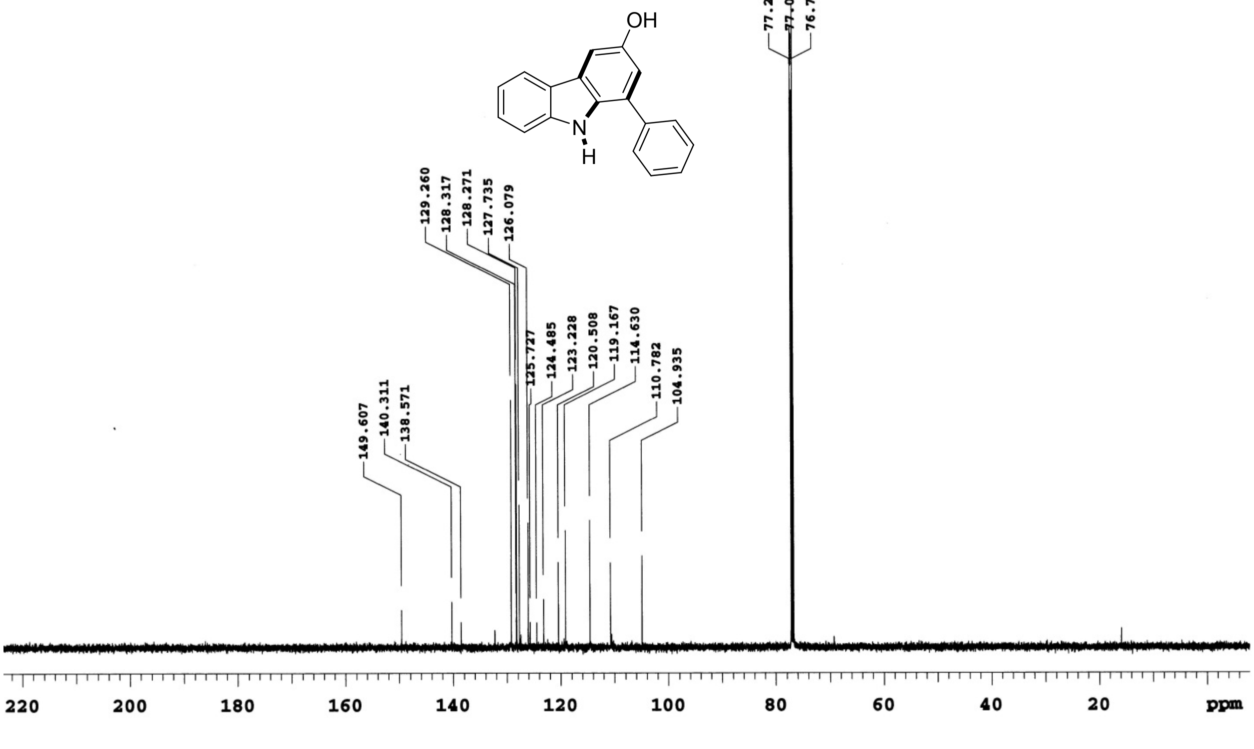

Compound-**21b**
^{1}H NMR/$CDCl_3$

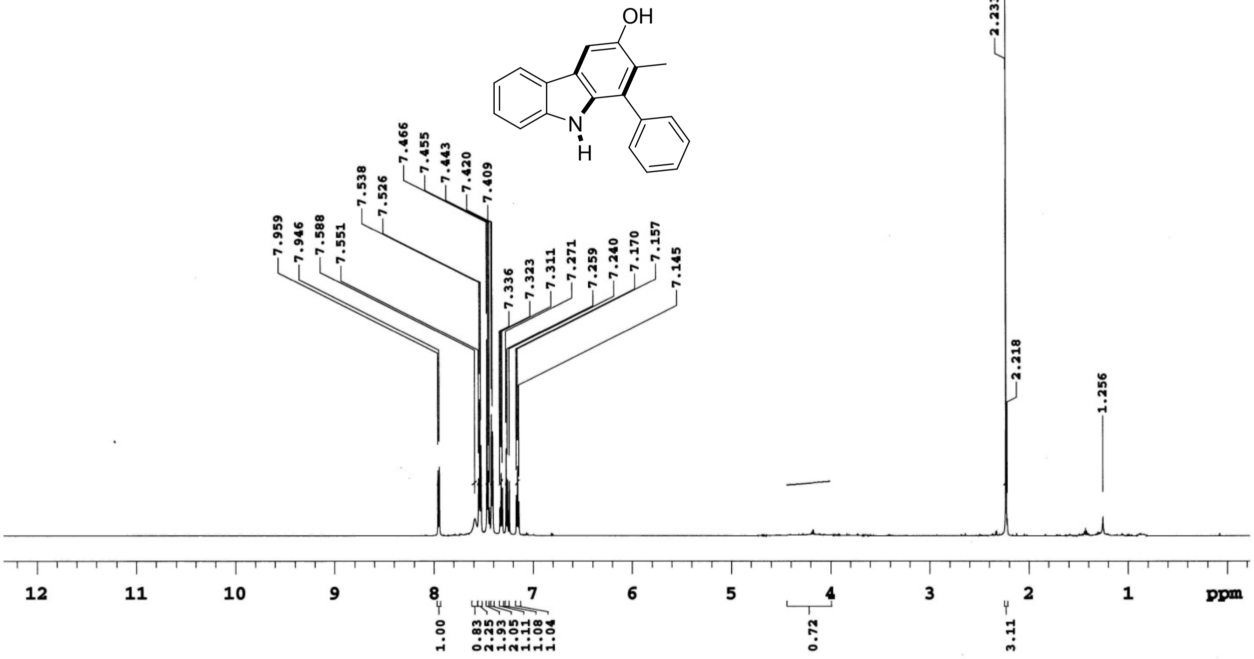

Compound-**21b**
^{13}C NMR/$CDCl_3$

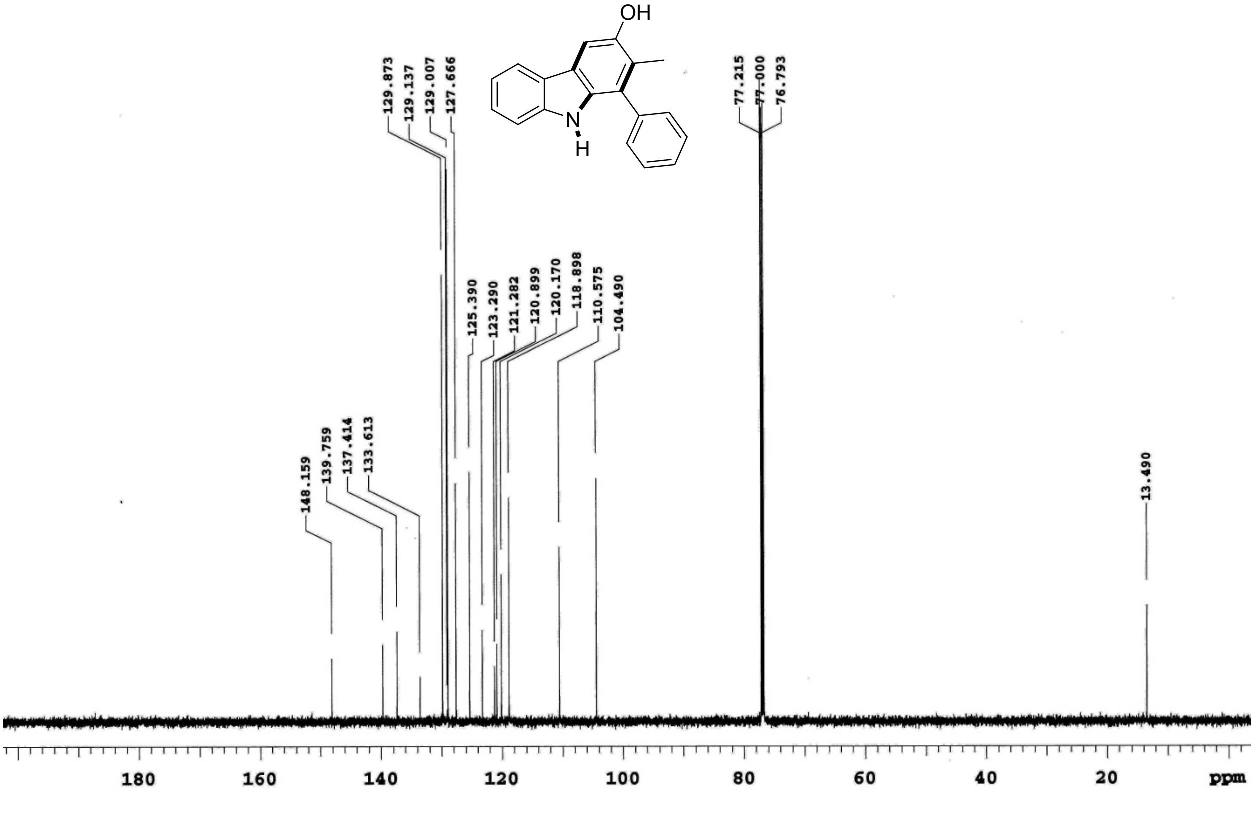

Compound-**21c**
^{1}H NMR/$CDCl_3$

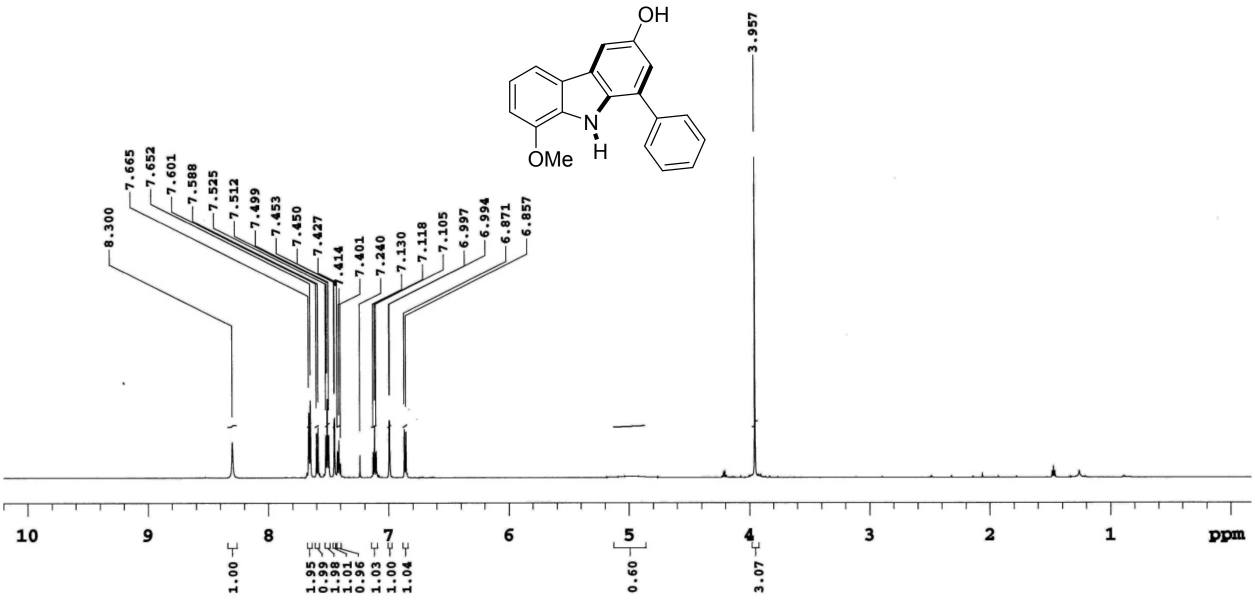

Compound-**21c**
^{13}C NMR/$CDCl_3$

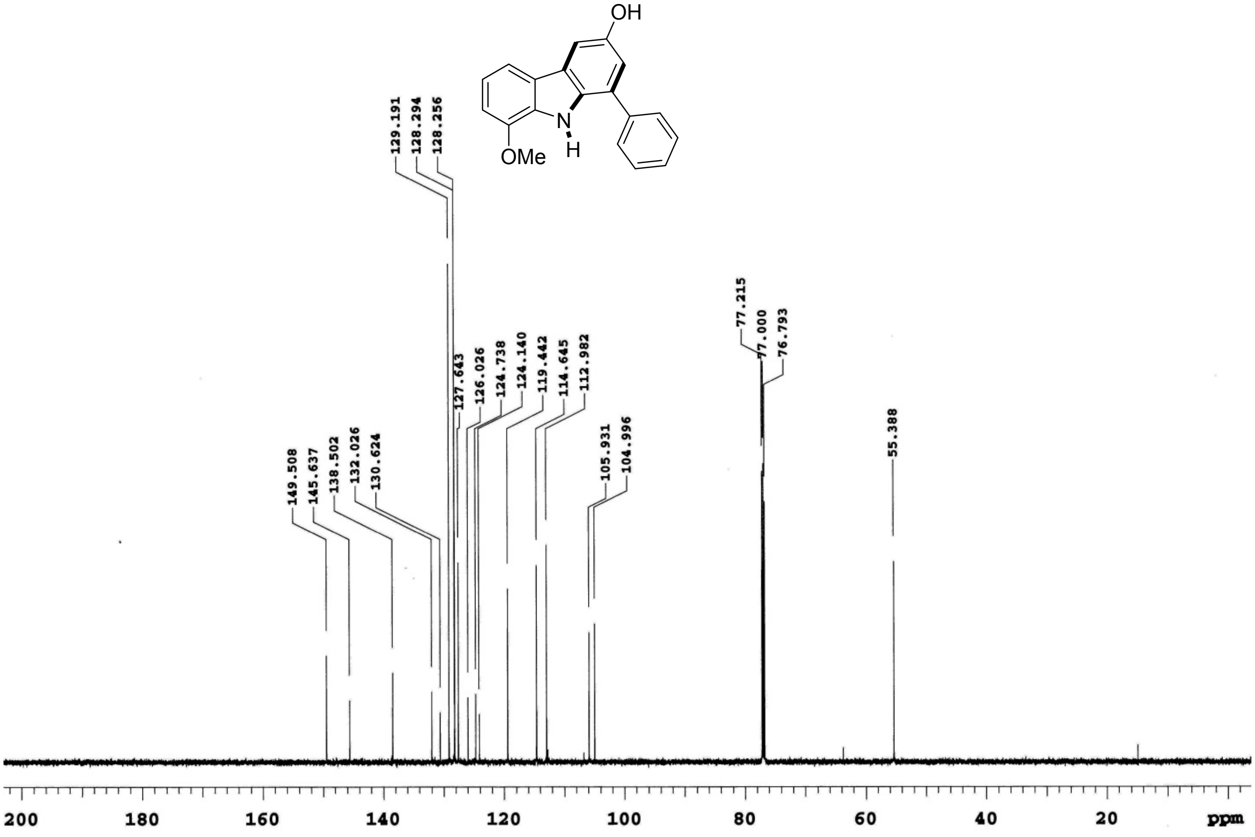

Compound-**21d**
^1H NMR/$CDCl_3$

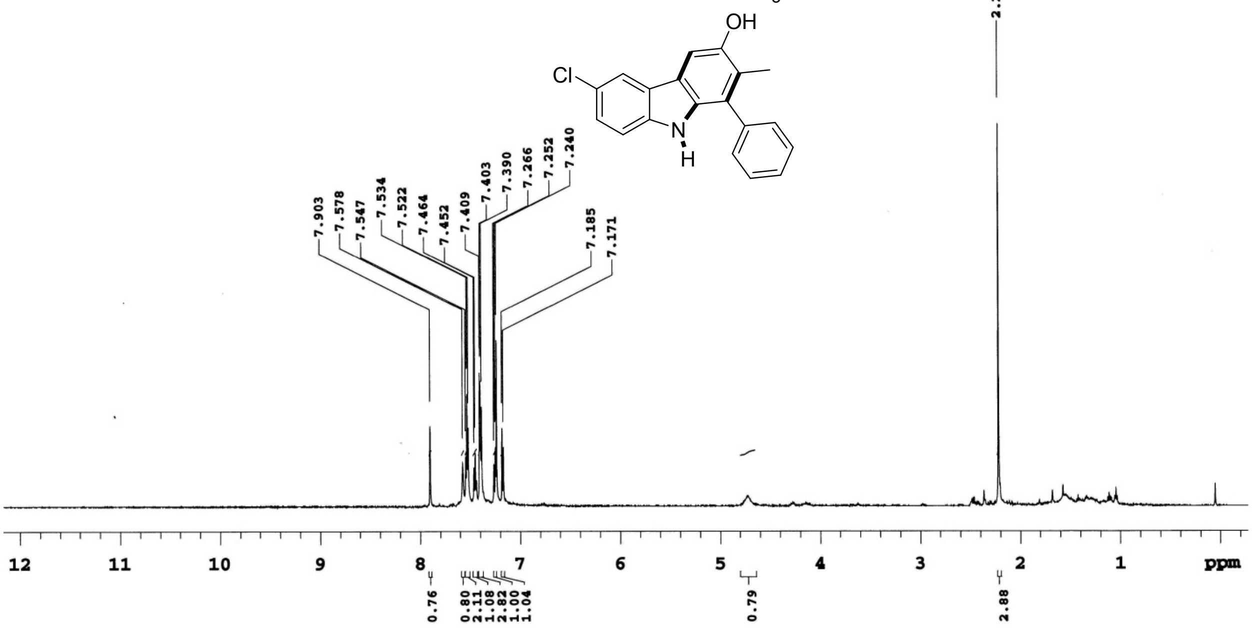

Compound-**21d**
^{13}C NMR/$CDCl_3$

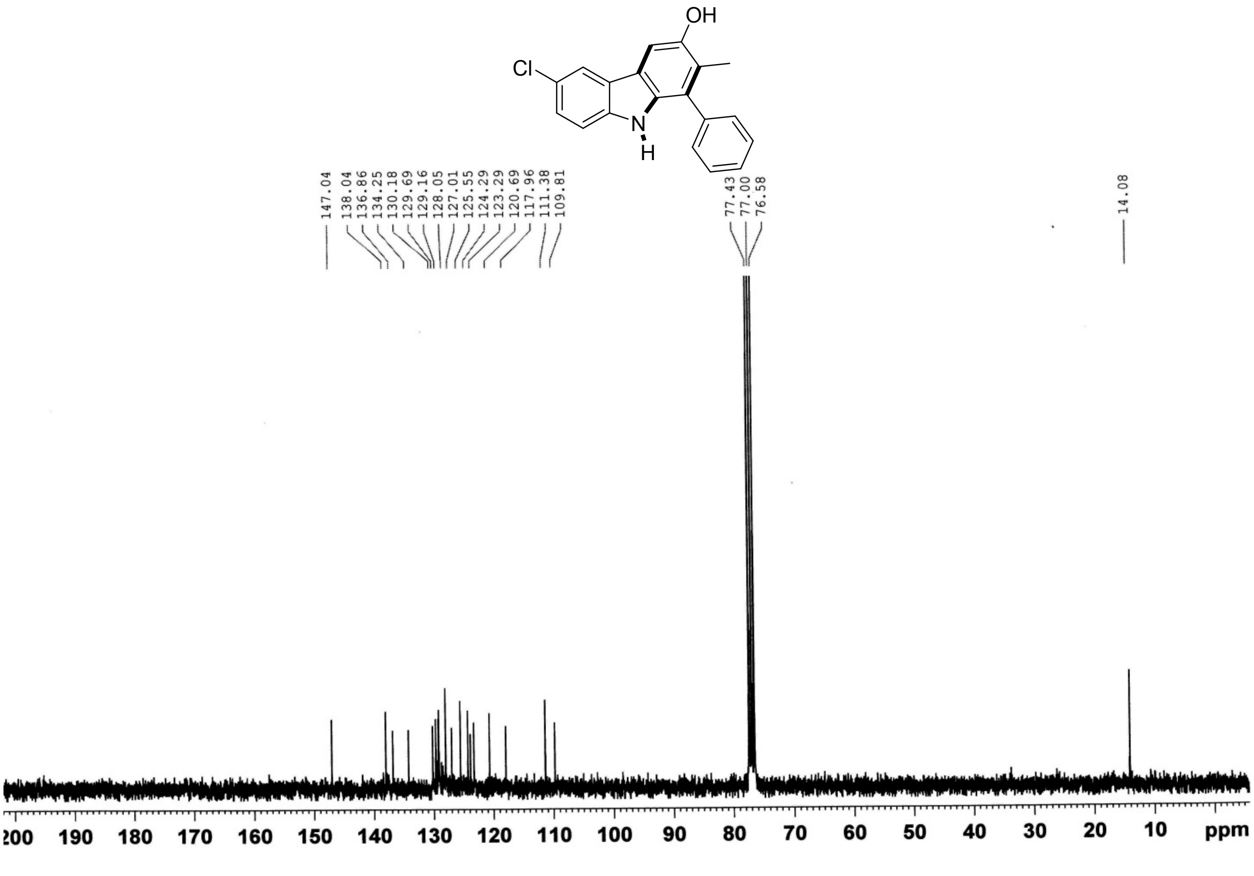

Compound-**22, Hyellazole**
^{1}H NMR/CDCl$_3$

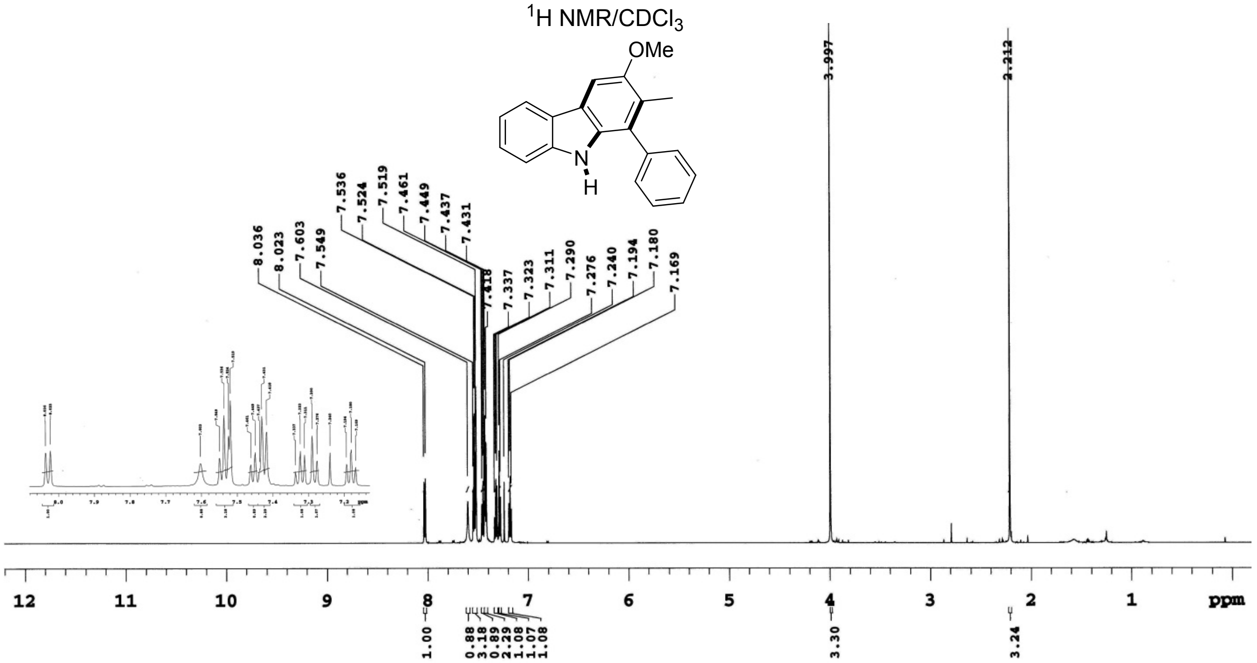

Compound-**22, Hyellazole**
^{13}C NMR/CDCl$_3$

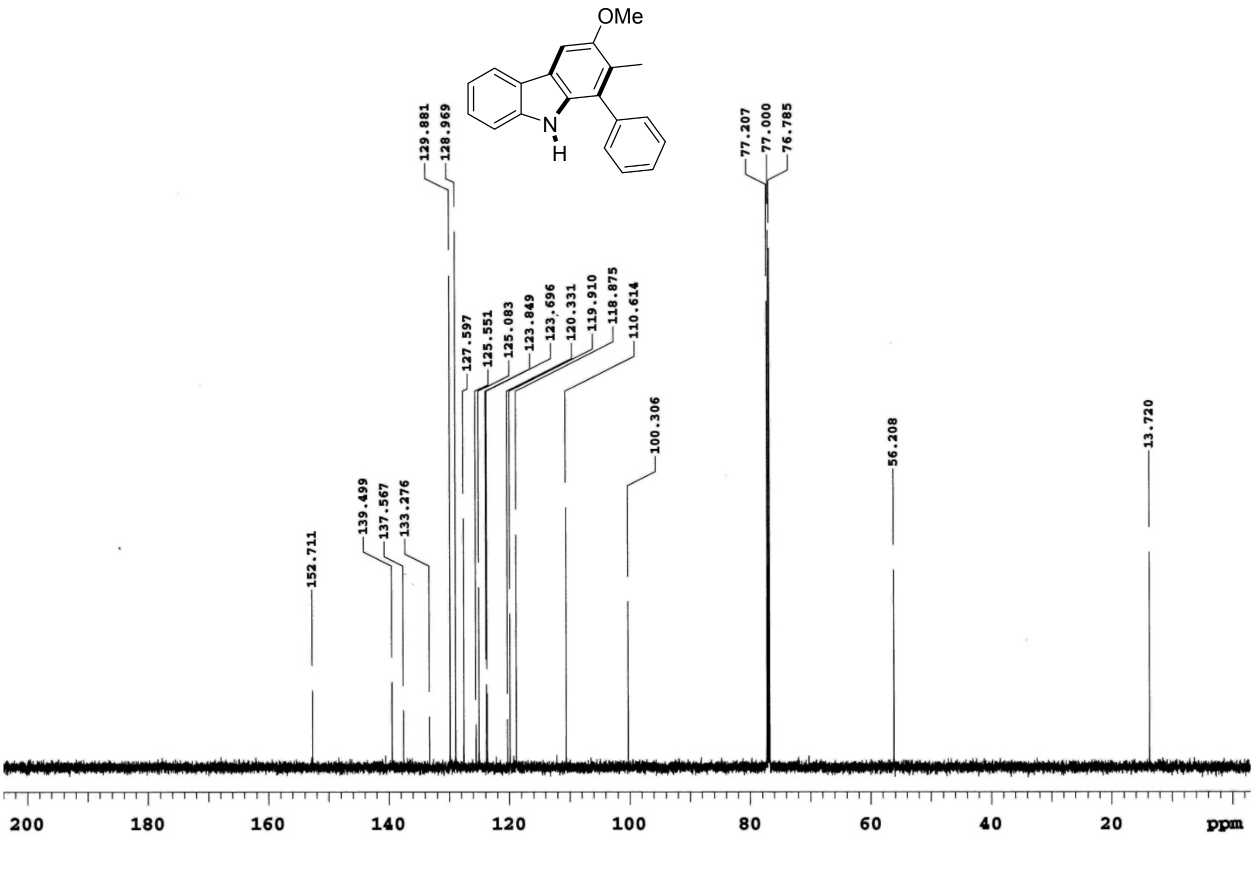

Compound-**23, Chlorohyellazole**

^{1}H NMR/$CDCl_3$

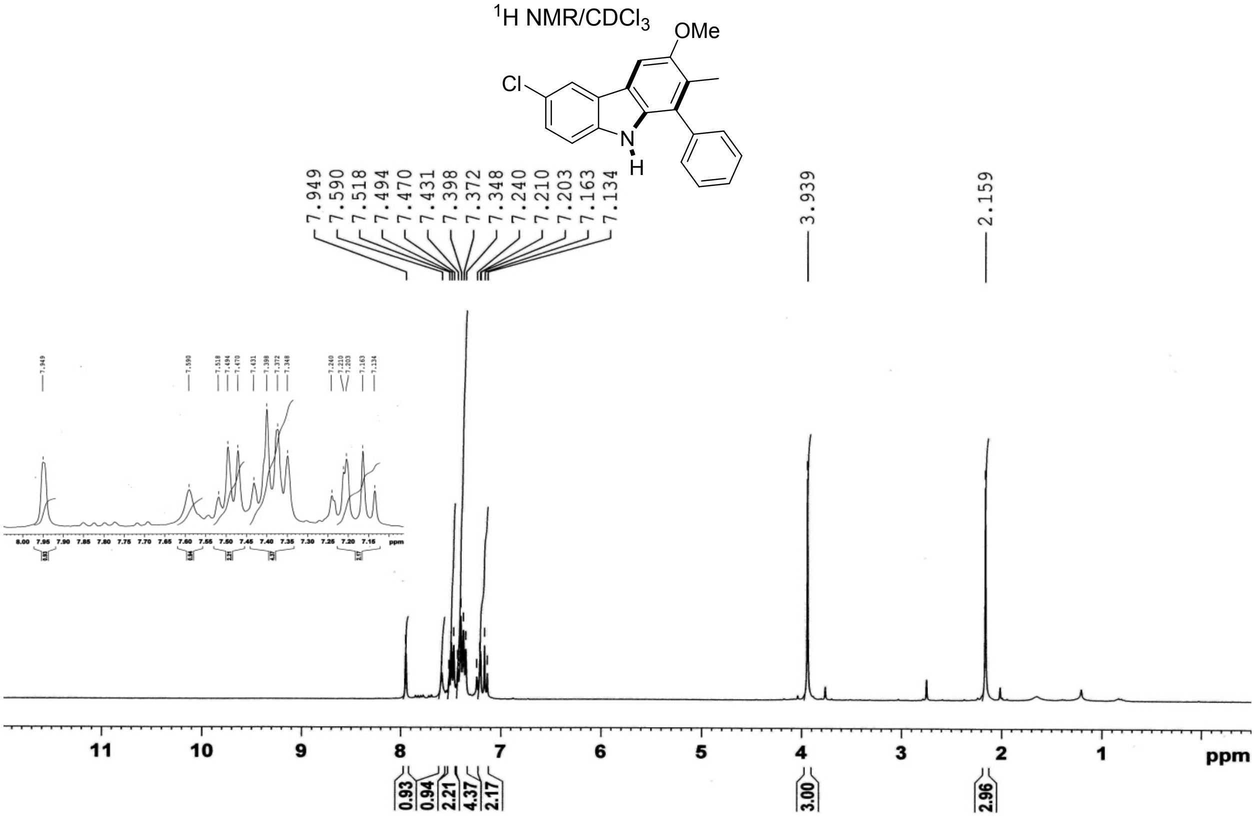

Compound-**23, Chlorohyellazole**

^{13}C NMR/$CDCl_3$

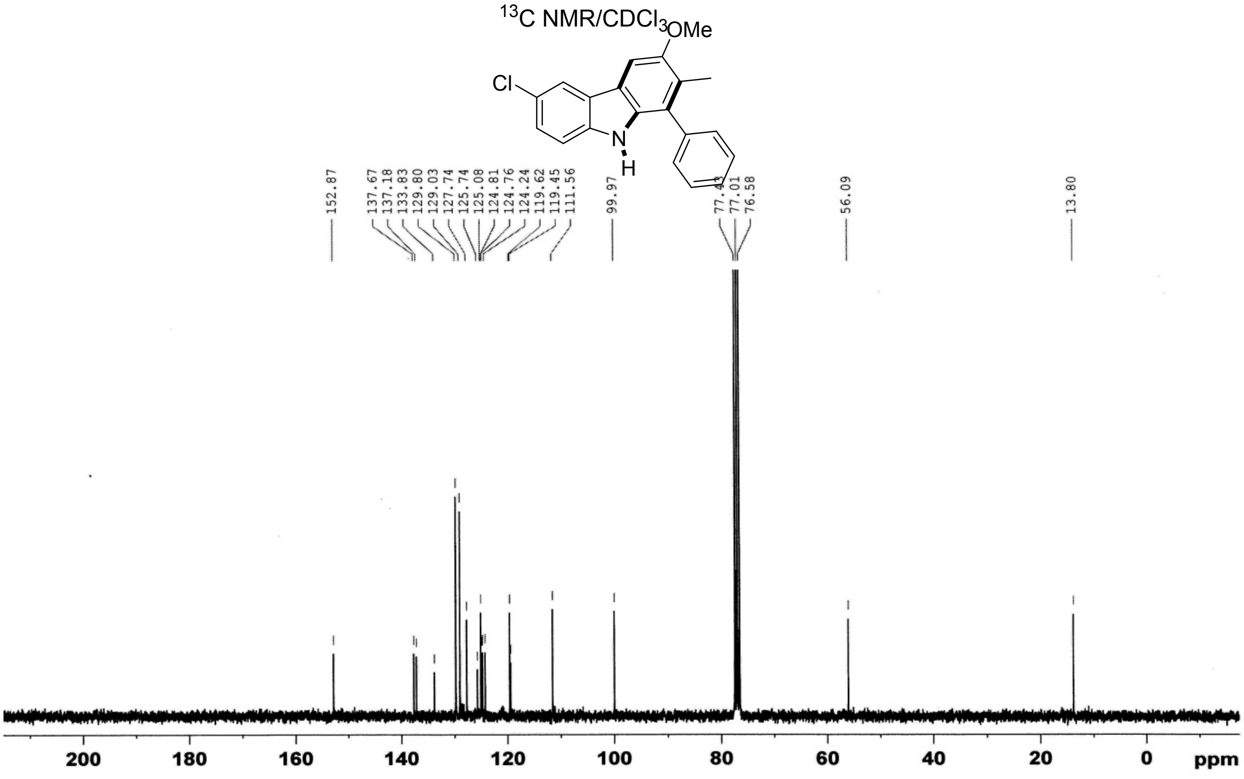

Crystal refinement data for compound 7a: Empirical Formula- $C_{24}H_{17}NO$, M = 335.39, Monoclinic, Space group P_{bca}, a = 12.9997(10) A°, b = 21.3361 (15) A°, c = 13.5020 (10) A°, V = 3592.6(5) A^{o3}, Z = 8, T = 200(2) K, ρ_{calcd} = 1.240 mg/m^3, $2\Theta_{max.}$ = 26.03^0, Refinement of 471 parameters on 7075 independent reflections out of 22336 collected reflections (R_{int} = 0.0875) led to R_1 = 0.0484 [I >2σ(I)], wR_2 = 0.1209 (all data) and S = 0.906 with the largest difference peak and hole of 0.158 and -0.195 e.A^{o-3} respectively. The crystal structure has been deposited at the Cambridge Crystallographic Data Centre (CCDC 1046362). The data can be obtained free of charge via the Internet at www.ccdc.cam.ac.uk/data_request/cif.

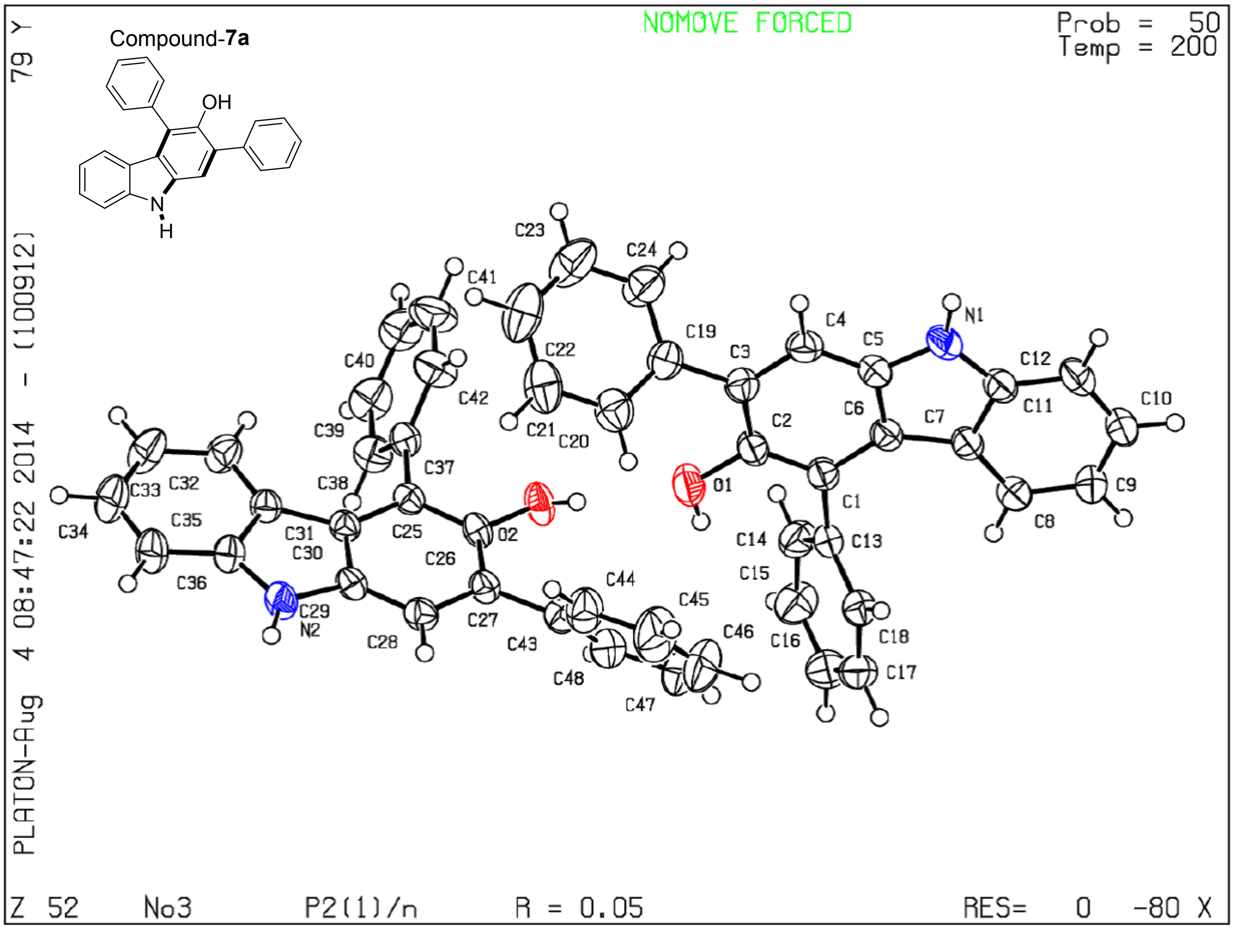

Supplementary Figure 1. X-ray Structure of compound **7a** containing two molecules in a unit.

Table 1. Crystal data and structure refinement for Compound No3

Identification code	No3	
Empirical formula	$C_{24} H_{17} N O$	
Formula weight	335.39	
Temperature	200(2) K	
Wavelength	0.71073 Å	
Crystal system	Monoclinic	
Space group	P2(1)/n	
Unit cell dimensions	a = 12.9997(10) Å	α= 90°.
	b = 21.3361(15) Å	β= 106.398(2)°.
	c = 13.5020(10) Å	γ = 90°.
Volume	3592.6(5) $Å^3$	
Z	8	
Density (calculated)	1.240 Mg/m^3	
Absorption coefficient	0.075 mm^{-1}	
F(000)	1408	
Crystal size	0.51 x 0.35 x 0.14 mm^3	
Theta range for data collection	1.84 to 26.03°.	
Index ranges	-16<=h<=10, -26<=k<=25, -16<=l<=16	
Reflections collected	22336	
Independent reflections	7075 [R(int) = 0.0875]	
Completeness to theta = 26.03°	99.9 %	
Absorption correction	None	
Refinement method	Full-matrix least-squares on F^2	
Data / restraints / parameters	7075 / 0 / 471	
Goodness-of-fit on F^2	0.906	
Final R indices [I>2sigma(I)]	R1 = 0.0484, wR2 = 0.0942	
R indices (all data)	R1 = 0.1063, wR2 = 0.1209	
Largest diff. peak and hole	0.158 and -0.195 e.$Å^{-3}$	

Table 2. Atomic coordinates (x 10^4) and equivalent isotropic displacement parameters (Å^{2}x 10^3)
for No3. U(eq) is defined as one third of the trace of the orthogonalized U^{ij} tensor.

	x	y	z	U(eq)
C(1)	3954(2)	1498(1)	1457(1)	36(1)
C(2)	4299(2)	1985(1)	2155(1)	40(1)
O(1)	4239(2)	1923(1)	3161(1)	53(1)
C(3)	4707(2)	2557(1)	1908(2)	40(1)
C(4)	4773(2)	2639(1)	907(2)	42(1)
C(5)	4449(2)	2159(1)	205(2)	39(1)
C(6)	4047(2)	1585(1)	449(1)	35(1)
C(7)	3809(2)	1205(1)	-480(1)	37(1)
C(8)	3393(2)	604(1)	-747(2)	42(1)
C(9)	3277(2)	386(1)	-1735(2)	47(1)
C(10)	3576(2)	754(1)	-2457(2)	50(1)
C(11)	3990(2)	1348(1)	-2224(2)	49(1)
C(12)	4096(2)	1565(1)	-1232(2)	41(1)
N(1)	4465(2)	2140(1)	-817(1)	47(1)
C(13)	3526(2)	913(1)	1801(1)	36(1)
C(14)	2612(2)	932(1)	2133(2)	48(1)
C(15)	2255(2)	403(1)	2538(2)	58(1)
C(16)	2808(2)	-152(1)	2603(2)	57(1)
C(17)	3709(2)	-181(1)	2267(2)	50(1)
C(18)	4070(2)	346(1)	1869(2)	43(1)
C(19)	5075(2)	3063(1)	2689(2)	44(1)
C(20)	5804(2)	2940(1)	3644(2)	54(1)
C(21)	6151(2)	3413(1)	4362(2)	63(1)
C(22)	5783(2)	4014(1)	4134(2)	74(1)
C(23)	5081(2)	4144(1)	3191(2)	75(1)
C(24)	4722(2)	3671(1)	2471(2)	59(1)
C(25)	4753(2)	2866(1)	6641(1)	32(1)
C(26)	5017(2)	2409(1)	6020(1)	33(1)

O(2)	4261(1)	2309(1)	5077(1)	45(1)
C(27)	5957(2)	2055(1)	6320(1)	35(1)
C(28)	6685(2)	2151(1)	7285(1)	37(1)
C(29)	6439(2)	2606(1)	7920(1)	35(1)
C(30)	5503(2)	2968(1)	7613(1)	32(1)
C(31)	5540(2)	3408(1)	8444(1)	35(1)
C(32)	4885(2)	3888(1)	8605(2)	46(1)
C(33)	5183(2)	4217(1)	9520(2)	58(1)
C(34)	6136(2)	4079(1)	10278(2)	58(1)
C(35)	6811(2)	3610(1)	10143(2)	52(1)
C(36)	6504(2)	3278(1)	9218(2)	40(1)
N(2)	7031(1)	2792(1)	8900(1)	42(1)
C(37)	3738(2)	3222(1)	6283(1)	36(1)
C(38)	2918(2)	3132(1)	6734(2)	44(1)
C(39)	1959(2)	3453(1)	6388(2)	54(1)
C(40)	1808(2)	3864(1)	5577(2)	59(1)
C(41)	2611(2)	3960(1)	5120(2)	64(1)
C(42)	3569(2)	3641(1)	5471(2)	53(1)
C(43)	6209(2)	1585(1)	5602(2)	40(1)
C(44)	7128(2)	1650(1)	5299(2)	55(1)
C(45)	7364(3)	1240(1)	4603(2)	81(1)
C(46)	6676(3)	755(2)	4208(2)	89(1)
C(47)	5756(3)	676(1)	4508(2)	73(1)
C(48)	5517(2)	1091(1)	5204(2)	52(1)

Table 3. Bond lengths [Å] and angles [°] for No3.

C(1)-C(2)	1.389(3)
C(1)-C(6)	1.413(2)
C(1)-C(13)	1.491(3)
C(2)-O(1)	1.388(2)
C(2)-C(3)	1.408(3)
O(1)-H(1)	0.8400
C(3)-C(4)	1.389(3)
C(3)-C(19)	1.490(3)
C(4)-C(5)	1.378(3)
C(4)-H(4)	0.9500
C(5)-N(1)	1.386(2)
C(5)-C(6)	1.406(3)
C(6)-C(7)	1.452(3)
C(7)-C(8)	1.399(3)
C(7)-C(12)	1.406(3)
C(8)-C(9)	1.380(3)
C(8)-H(8)	0.9500
C(9)-C(10)	1.389(3)
C(9)-H(9)	0.9500
C(10)-C(11)	1.377(3)
C(10)-H(10)	0.9500
C(11)-C(12)	1.386(3)
C(11)-H(11)	0.9500
C(12)-N(1)	1.379(3)
N(1)-H(1A)	0.8800
C(13)-C(14)	1.383(3)
C(13)-C(18)	1.391(3)
C(14)-C(15)	1.390(3)
C(14)-H(14)	0.9500
C(15)-C(16)	1.376(3)
C(15)-H(15)	0.9500
C(16)-C(17)	1.371(3)
C(16)-H(16)	0.9500
C(17)-C(18)	1.384(3)

C(17)-H(17)	0.9500
C(18)-H(18)	0.9500
C(19)-C(24)	1.381(3)
C(19)-C(20)	1.393(3)
C(20)-C(21)	1.383(3)
C(20)-H(20)	0.9500
C(21)-C(22)	1.372(4)
C(21)-H(21)	0.9500
C(22)-C(23)	1.370(4)
C(22)-H(22)	0.9500
C(23)-C(24)	1.387(3)
C(23)-H(23)	0.9500
C(24)-H(24)	0.9500
C(25)-C(26)	1.391(3)
C(25)-C(30)	1.413(3)
C(25)-C(37)	1.480(3)
C(26)-O(2)	1.387(2)
C(26)-C(27)	1.397(3)
O(2)-H(2)	0.8400
C(27)-C(28)	1.392(3)
C(27)-C(43)	1.493(3)
C(28)-C(29)	1.391(3)
C(28)-H(28)	0.9500
C(29)-N(2)	1.387(2)
C(29)-C(30)	1.403(3)
C(30)-C(31)	1.453(3)
C(31)-C(32)	1.389(3)
C(31)-C(36)	1.414(3)
C(32)-C(33)	1.378(3)
C(32)-H(32)	0.9500
C(33)-C(34)	1.397(3)
C(33)-H(33)	0.9500
C(34)-C(35)	1.377(3)
C(34)-H(34)	0.9500
C(35)-C(36)	1.393(3)
C(35)-H(35)	0.9500

C(36)-N(2)	1.375(3)
N(2)-H(2A)	0.8800
C(37)-C(38)	1.382(3)
C(37)-C(42)	1.384(3)
C(38)-C(39)	1.381(3)
C(38)-H(38)	0.9500
C(39)-C(40)	1.374(3)
C(39)-H(39)	0.9500
C(40)-C(41)	1.370(4)
C(40)-H(40)	0.9500
C(41)-C(42)	1.380(3)
C(41)-H(41)	0.9500
C(42)-H(42)	0.9500
C(43)-C(44)	1.375(3)
C(43)-C(48)	1.391(3)
C(44)-C(45)	1.381(3)
C(44)-H(44)	0.9500
C(45)-C(46)	1.374(4)
C(45)-H(45)	0.9500
C(46)-C(47)	1.377(4)
C(46)-H(46)	0.9500
C(47)-C(48)	1.388(3)
C(47)-H(47)	0.9500
C(48)-H(48)	0.9500
C(2)-C(1)-C(6)	117.10(19)
C(2)-C(1)-C(13)	119.41(17)
C(6)-C(1)-C(13)	123.47(18)
O(1)-C(2)-C(1)	119.71(18)
O(1)-C(2)-C(3)	116.34(18)
C(1)-C(2)-C(3)	123.95(18)
C(2)-O(1)-H(1)	109.5
C(4)-C(3)-C(2)	118.14(19)
C(4)-C(3)-C(19)	120.04(19)
C(2)-C(3)-C(19)	121.82(18)
C(5)-C(4)-C(3)	118.9(2)

C(5)-C(4)-H(4)	120.5
C(3)-C(4)-H(4)	120.5
C(4)-C(5)-N(1)	128.25(19)
C(4)-C(5)-C(6)	123.19(19)
N(1)-C(5)-C(6)	108.56(18)
C(5)-C(6)-C(1)	118.64(18)
C(5)-C(6)-C(7)	106.72(17)
C(1)-C(6)-C(7)	134.63(19)
C(8)-C(7)-C(12)	118.52(18)
C(8)-C(7)-C(6)	135.06(19)
C(12)-C(7)-C(6)	106.42(18)
C(9)-C(8)-C(7)	119.03(19)
C(9)-C(8)-H(8)	120.5
C(7)-C(8)-H(8)	120.5
C(8)-C(9)-C(10)	120.8(2)
C(8)-C(9)-H(9)	119.6
C(10)-C(9)-H(9)	119.6
C(11)-C(10)-C(9)	121.9(2)
C(11)-C(10)-H(10)	119.1
C(9)-C(10)-H(10)	119.1
C(10)-C(11)-C(12)	117.0(2)
C(10)-C(11)-H(11)	121.5
C(12)-C(11)-H(11)	121.5
N(1)-C(12)-C(11)	128.3(2)
N(1)-C(12)-C(7)	108.99(17)
C(11)-C(12)-C(7)	122.7(2)
C(12)-N(1)-C(5)	109.29(17)
C(12)-N(1)-H(1A)	125.4
C(5)-N(1)-H(1A)	125.4
C(14)-C(13)-C(18)	118.0(2)
C(14)-C(13)-C(1)	120.42(19)
C(18)-C(13)-C(1)	121.4(2)
C(13)-C(14)-C(15)	121.1(2)
C(13)-C(14)-H(14)	119.4
C(15)-C(14)-H(14)	119.4
C(16)-C(15)-C(14)	119.8(2)

C(16)-C(15)-H(15)	120.1
C(14)-C(15)-H(15)	120.1
C(17)-C(16)-C(15)	119.8(2)
C(17)-C(16)-H(16)	120.1
C(15)-C(16)-H(16)	120.1
C(16)-C(17)-C(18)	120.4(2)
C(16)-C(17)-H(17)	119.8
C(18)-C(17)-H(17)	119.8
C(17)-C(18)-C(13)	120.7(2)
C(17)-C(18)-H(18)	119.6
C(13)-C(18)-H(18)	119.6
C(24)-C(19)-C(20)	118.2(2)
C(24)-C(19)-C(3)	120.6(2)
C(20)-C(19)-C(3)	121.1(2)
C(21)-C(20)-C(19)	120.8(2)
C(21)-C(20)-H(20)	119.6
C(19)-C(20)-H(20)	119.6
C(22)-C(21)-C(20)	120.1(2)
C(22)-C(21)-H(21)	120.0
C(20)-C(21)-H(21)	120.0
C(23)-C(22)-C(21)	119.8(2)
C(23)-C(22)-H(22)	120.1
C(21)-C(22)-H(22)	120.1
C(22)-C(23)-C(24)	120.5(3)
C(22)-C(23)-H(23)	119.7
C(24)-C(23)-H(23)	119.7
C(19)-C(24)-C(23)	120.6(2)
C(19)-C(24)-H(24)	119.7
C(23)-C(24)-H(24)	119.7
C(26)-C(25)-C(30)	116.42(19)
C(26)-C(25)-C(37)	120.75(17)
C(30)-C(25)-C(37)	122.83(17)
O(2)-C(26)-C(25)	115.44(18)
O(2)-C(26)-C(27)	121.20(17)
C(25)-C(26)-C(27)	123.33(17)
C(26)-O(2)-H(2)	109.5

C(28)-C(27)-C(26)	119.98(18)
C(28)-C(27)-C(43)	119.39(19)
C(26)-C(27)-C(43)	120.61(17)
C(29)-C(28)-C(27)	117.8(2)
C(29)-C(28)-H(28)	121.1
C(27)-C(28)-H(28)	121.1
N(2)-C(29)-C(28)	128.8(2)
N(2)-C(29)-C(30)	108.96(17)
C(28)-C(29)-C(30)	122.23(18)
C(29)-C(30)-C(25)	120.23(18)
C(29)-C(30)-C(31)	106.88(17)
C(25)-C(30)-C(31)	132.89(19)
C(32)-C(31)-C(36)	118.94(19)
C(32)-C(31)-C(30)	135.2(2)
C(36)-C(31)-C(30)	105.89(18)
C(33)-C(32)-C(31)	119.1(2)
C(33)-C(32)-H(32)	120.5
C(31)-C(32)-H(32)	120.5
C(32)-C(33)-C(34)	121.1(2)
C(32)-C(33)-H(33)	119.4
C(34)-C(33)-H(33)	119.4
C(35)-C(34)-C(33)	121.5(2)
C(35)-C(34)-H(34)	119.3
C(33)-C(34)-H(34)	119.3
C(34)-C(35)-C(36)	117.2(2)
C(34)-C(35)-H(35)	121.4
C(36)-C(35)-H(35)	121.4
N(2)-C(36)-C(35)	128.4(2)
N(2)-C(36)-C(31)	109.39(17)
C(35)-C(36)-C(31)	122.2(2)
C(36)-N(2)-C(29)	108.86(17)
C(36)-N(2)-H(2A)	125.6
C(29)-N(2)-H(2A)	125.6
C(38)-C(37)-C(42)	117.9(2)
C(38)-C(37)-C(25)	120.83(18)
C(42)-C(37)-C(25)	121.2(2)

C(39)-C(38)-C(37)	121.2(2)
C(39)-C(38)-H(38)	119.4
C(37)-C(38)-H(38)	119.4
C(40)-C(39)-C(38)	119.9(2)
C(40)-C(39)-H(39)	120.1
C(38)-C(39)-H(39)	120.1
C(41)-C(40)-C(39)	119.9(2)
C(41)-C(40)-H(40)	120.0
C(39)-C(40)-H(40)	120.0
C(40)-C(41)-C(42)	120.0(2)
C(40)-C(41)-H(41)	120.0
C(42)-C(41)-H(41)	120.0
C(41)-C(42)-C(37)	121.1(2)
C(41)-C(42)-H(42)	119.4
C(37)-C(42)-H(42)	119.4
C(44)-C(43)-C(48)	118.9(2)
C(44)-C(43)-C(27)	119.5(2)
C(48)-C(43)-C(27)	121.6(2)
C(43)-C(44)-C(45)	121.1(3)
C(43)-C(44)-H(44)	119.4
C(45)-C(44)-H(44)	119.4
C(46)-C(45)-C(44)	119.7(3)
C(46)-C(45)-H(45)	120.1
C(44)-C(45)-H(45)	120.1
C(45)-C(46)-C(47)	120.2(3)
C(45)-C(46)-H(46)	119.9
C(47)-C(46)-H(46)	119.9
C(46)-C(47)-C(48)	120.0(3)
C(46)-C(47)-H(47)	120.0
C(48)-C(47)-H(47)	120.0
C(47)-C(48)-C(43)	120.1(3)
C(47)-C(48)-H(48)	120.0
C(43)-C(48)-H(48)	120.0

Symmetry transformations used to generate equivalent atoms:

Table 4. Anisotropic displacement parameters ($Å^2 x 10^3$) for No3. The anisotropic displacement factor exponent takes the form: $-2\pi^2[h^2 a^{*2}U^{11} + ... + 2 h k a^* b^* U^{12}]$

	U^{11}	U^{22}	U^{33}	U^{23}	U^{13}	U^{12}
C(1)	37(1)	39(1)	32(1)	0(1)	10(1)	2(1)
C(2)	44(2)	46(1)	29(1)	0(1)	10(1)	2(1)
O(1)	76(1)	51(1)	35(1)	-7(1)	20(1)	-18(1)
C(3)	41(2)	39(1)	38(1)	-3(1)	7(1)	1(1)
C(4)	47(2)	36(1)	43(1)	5(1)	11(1)	-2(1)
C(5)	42(2)	41(1)	32(1)	4(1)	9(1)	2(1)
C(6)	35(1)	38(1)	32(1)	2(1)	8(1)	2(1)
C(7)	38(1)	41(1)	30(1)	2(1)	8(1)	4(1)
C(8)	46(2)	44(1)	36(1)	3(1)	11(1)	2(1)
C(9)	56(2)	47(1)	36(1)	-6(1)	9(1)	3(1)
C(10)	62(2)	56(2)	31(1)	-1(1)	9(1)	9(1)
C(11)	59(2)	57(2)	31(1)	7(1)	14(1)	0(1)
C(12)	44(2)	46(1)	33(1)	4(1)	9(1)	6(1)
N(1)	64(2)	44(1)	34(1)	7(1)	17(1)	-5(1)
C(13)	42(2)	38(1)	27(1)	-2(1)	8(1)	1(1)
C(14)	55(2)	41(1)	52(1)	-2(1)	23(1)	3(1)
C(15)	64(2)	49(2)	70(2)	-2(1)	36(2)	-8(2)
C(16)	71(2)	44(2)	57(2)	4(1)	20(2)	-13(2)
C(17)	57(2)	40(1)	47(1)	2(1)	3(1)	2(1)
C(18)	45(2)	43(1)	37(1)	-3(1)	7(1)	1(1)
C(19)	41(2)	45(1)	46(1)	-6(1)	12(1)	-4(1)
C(20)	49(2)	59(2)	50(1)	-5(1)	9(1)	-9(1)
C(21)	49(2)	84(2)	51(2)	-18(2)	6(1)	-22(2)
C(22)	58(2)	78(2)	85(2)	-38(2)	21(2)	-27(2)
C(23)	65(2)	52(2)	98(2)	-24(2)	9(2)	-1(2)
C(24)	57(2)	45(1)	67(2)	-9(1)	5(1)	3(1)
C(25)	30(1)	35(1)	29(1)	1(1)	5(1)	-1(1)
C(26)	27(1)	45(1)	25(1)	-1(1)	2(1)	-3(1)
O(2)	34(1)	66(1)	28(1)	-9(1)	-1(1)	4(1)
C(27)	33(1)	39(1)	29(1)	0(1)	5(1)	1(1)
C(28)	31(1)	43(1)	34(1)	1(1)	4(1)	6(1)

C(29)	32(1)	41(1)	28(1)	1(1)	2(1)	1(1)
C(30)	31(1)	35(1)	29(1)	0(1)	4(1)	-2(1)
C(31)	34(1)	36(1)	32(1)	-2(1)	4(1)	-3(1)
C(32)	42(2)	39(1)	53(1)	-10(1)	6(1)	-4(1)
C(33)	52(2)	47(1)	71(2)	-23(1)	11(1)	-2(1)
C(34)	59(2)	57(2)	55(2)	-25(1)	8(1)	-11(2)
C(35)	51(2)	59(2)	40(1)	-14(1)	1(1)	-9(1)
C(36)	40(2)	42(1)	37(1)	-7(1)	8(1)	-4(1)
N(2)	34(1)	52(1)	32(1)	-5(1)	-5(1)	6(1)
C(37)	33(1)	40(1)	31(1)	-2(1)	3(1)	-2(1)
C(38)	35(2)	49(1)	46(1)	2(1)	7(1)	0(1)
C(39)	37(2)	59(2)	67(2)	-5(1)	15(1)	4(1)
C(40)	41(2)	60(2)	66(2)	-6(1)	-2(1)	15(1)
C(41)	64(2)	66(2)	58(2)	22(1)	9(2)	21(2)
C(42)	50(2)	62(2)	48(1)	14(1)	15(1)	10(1)
C(43)	43(2)	42(1)	31(1)	0(1)	4(1)	6(1)
C(44)	50(2)	65(2)	52(2)	-11(1)	15(1)	8(1)
C(45)	78(2)	98(2)	77(2)	-16(2)	37(2)	22(2)
C(46)	121(3)	77(2)	71(2)	-27(2)	32(2)	22(2)
C(47)	102(3)	52(2)	52(2)	-18(1)	2(2)	1(2)
C(48)	60(2)	49(1)	44(1)	-4(1)	7(1)	0(1)

Table 5. Hydrogen coordinates (x 10^4) and isotropic displacement parameters ($Å^2$x 10^3) for No3.

	x	y	z	U(eq)
H(1)	3986	1569	3235	79
H(4)	5036	3021	709	51
H(8)	3192	349	-255	50
H(9)	2990	-20	-1924	56
H(10)	3493	592	-3130	60
H(11)	4193	1597	-2720	58
H(1A)	4677	2447	-1148	56
H(14)	2222	1313	2082	57
H(15)	1631	425	2770	69
H(16)	2566	-514	2879	68
H(17)	4087	-565	2307	60
H(18)	4696	321	1641	51
H(20)	6066	2525	3805	64
H(21)	6643	3321	5012	76
H(22)	6015	4338	4629	88
H(23)	4838	4562	3029	90
H(24)	4230	3767	1822	71
H(2)	4574	2227	4627	67
H(28)	7328	1913	7502	45
H(32)	4240	3989	8090	55
H(33)	4733	4542	9639	69
H(34)	6323	4315	10900	70
H(35)	7459	3517	10659	62
H(2A)	7642	2627	9259	50
H(38)	3015	2846	7291	53
H(39)	1406	3388	6710	65
H(40)	1147	4082	5334	71
H(41)	2509	4246	4561	77

H(42)	4121	3711	5149	64
H(44)	7610	1983	5574	66
H(45)	8001	1293	4398	98
H(46)	6834	474	3726	107
H(47)	5285	337	4240	87
H(48)	4880	1038	5408	63

Table 6. Torsion angles [°] for No3.

C(6)-C(1)-C(2)-O(1)	-179.23(19)
C(13)-C(1)-C(2)-O(1)	-0.2(3)
C(6)-C(1)-C(2)-C(3)	1.5(3)
C(13)-C(1)-C(2)-C(3)	-179.5(2)
O(1)-C(2)-C(3)-C(4)	-179.5(2)
C(1)-C(2)-C(3)-C(4)	-0.2(3)
O(1)-C(2)-C(3)-C(19)	1.2(3)
C(1)-C(2)-C(3)-C(19)	-179.5(2)
C(2)-C(3)-C(4)-C(5)	-0.8(3)
C(19)-C(3)-C(4)-C(5)	178.6(2)
C(3)-C(4)-C(5)-N(1)	-178.8(2)
C(3)-C(4)-C(5)-C(6)	0.4(3)
C(4)-C(5)-C(6)-C(1)	1.0(3)
N(1)-C(5)-C(6)-C(1)	-179.76(19)
C(4)-C(5)-C(6)-C(7)	-179.4(2)
N(1)-C(5)-C(6)-C(7)	-0.1(2)
C(2)-C(1)-C(6)-C(5)	-1.8(3)
C(13)-C(1)-C(6)-C(5)	179.2(2)
C(2)-C(1)-C(6)-C(7)	178.7(2)
C(13)-C(1)-C(6)-C(7)	-0.3(4)
C(5)-C(6)-C(7)-C(8)	-179.4(2)
C(1)-C(6)-C(7)-C(8)	0.1(4)
C(5)-C(6)-C(7)-C(12)	1.0(2)
C(1)-C(6)-C(7)-C(12)	-179.5(2)
C(12)-C(7)-C(8)-C(9)	-0.1(3)
C(6)-C(7)-C(8)-C(9)	-179.7(2)

C(7)-C(8)-C(9)-C(10)	0.5(3)
C(8)-C(9)-C(10)-C(11)	-0.4(4)
C(9)-C(10)-C(11)-C(12)	0.0(4)
C(10)-C(11)-C(12)-N(1)	-178.6(2)
C(10)-C(11)-C(12)-C(7)	0.4(3)
C(8)-C(7)-C(12)-N(1)	178.85(19)
C(6)-C(7)-C(12)-N(1)	-1.5(2)
C(8)-C(7)-C(12)-C(11)	-0.4(3)
C(6)-C(7)-C(12)-C(11)	179.3(2)
C(11)-C(12)-N(1)-C(5)	-179.4(2)
C(7)-C(12)-N(1)-C(5)	1.4(3)
C(4)-C(5)-N(1)-C(12)	178.4(2)
C(6)-C(5)-N(1)-C(12)	-0.8(3)
C(2)-C(1)-C(13)-C(14)	62.5(3)
C(6)-C(1)-C(13)-C(14)	-118.6(2)
C(2)-C(1)-C(13)-C(18)	-112.9(2)
C(6)-C(1)-C(13)-C(18)	66.1(3)
C(18)-C(13)-C(14)-C(15)	1.0(3)
C(1)-C(13)-C(14)-C(15)	-174.4(2)
C(13)-C(14)-C(15)-C(16)	-0.8(4)
C(14)-C(15)-C(16)-C(17)	0.0(4)
C(15)-C(16)-C(17)-C(18)	0.4(4)
C(16)-C(17)-C(18)-C(13)	-0.1(3)
C(14)-C(13)-C(18)-C(17)	-0.6(3)
C(1)-C(13)-C(18)-C(17)	174.85(19)
C(4)-C(3)-C(19)-C(24)	50.4(3)
C(2)-C(3)-C(19)-C(24)	-130.2(3)
C(4)-C(3)-C(19)-C(20)	-127.8(2)
C(2)-C(3)-C(19)-C(20)	51.6(3)
C(24)-C(19)-C(20)-C(21)	1.1(4)
C(3)-C(19)-C(20)-C(21)	179.3(2)
C(19)-C(20)-C(21)-C(22)	-0.5(4)
C(20)-C(21)-C(22)-C(23)	-0.7(4)
C(21)-C(22)-C(23)-C(24)	1.3(4)
C(20)-C(19)-C(24)-C(23)	-0.5(4)
C(3)-C(19)-C(24)-C(23)	-178.8(2)

C(22)-C(23)-C(24)-C(19)	-0.6(4)
C(30)-C(25)-C(26)-O(2)	178.96(17)
C(37)-C(25)-C(26)-O(2)	-1.4(3)
C(30)-C(25)-C(26)-C(27)	0.9(3)
C(37)-C(25)-C(26)-C(27)	-179.43(19)
O(2)-C(26)-C(27)-C(28)	-177.82(18)
C(25)-C(26)-C(27)-C(28)	0.1(3)
O(2)-C(26)-C(27)-C(43)	4.0(3)
C(25)-C(26)-C(27)-C(43)	-178.10(19)
C(26)-C(27)-C(28)-C(29)	-0.1(3)
C(43)-C(27)-C(28)-C(29)	178.07(19)
C(27)-C(28)-C(29)-N(2)	-179.2(2)
C(27)-C(28)-C(29)-C(30)	-0.9(3)
N(2)-C(29)-C(30)-C(25)	-179.39(18)
C(28)-C(29)-C(30)-C(25)	2.0(3)
N(2)-C(29)-C(30)-C(31)	1.2(2)
C(28)-C(29)-C(30)-C(31)	-177.41(19)
C(26)-C(25)-C(30)-C(29)	-1.9(3)
C(37)-C(25)-C(30)-C(29)	178.45(19)
C(26)-C(25)-C(30)-C(31)	177.3(2)
C(37)-C(25)-C(30)-C(31)	-2.3(3)
C(29)-C(30)-C(31)-C(32)	178.5(2)
C(25)-C(30)-C(31)-C(32)	-0.8(4)
C(29)-C(30)-C(31)-C(36)	-0.6(2)
C(25)-C(30)-C(31)-C(36)	-179.9(2)
C(36)-C(31)-C(32)-C(33)	-1.2(3)
C(30)-C(31)-C(32)-C(33)	179.8(2)
C(31)-C(32)-C(33)-C(34)	0.9(4)
C(32)-C(33)-C(34)-C(35)	-0.3(4)
C(33)-C(34)-C(35)-C(36)	0.0(4)
C(34)-C(35)-C(36)-N(2)	-179.8(2)
C(34)-C(35)-C(36)-C(31)	-0.4(3)
C(32)-C(31)-C(36)-N(2)	-179.52(19)
C(30)-C(31)-C(36)-N(2)	-0.2(2)
C(32)-C(31)-C(36)-C(35)	0.9(3)
C(30)-C(31)-C(36)-C(35)	-179.76(19)

C(35)-C(36)-N(2)-C(29)	-179.5(2)
C(31)-C(36)-N(2)-C(29)	1.0(2)
C(28)-C(29)-N(2)-C(36)	177.1(2)
C(30)-C(29)-N(2)-C(36)	-1.4(2)
C(26)-C(25)-C(37)-C(38)	110.6(2)
C(30)-C(25)-C(37)-C(38)	-69.8(3)
C(26)-C(25)-C(37)-C(42)	-67.7(3)
C(30)-C(25)-C(37)-C(42)	111.9(2)
C(42)-C(37)-C(38)-C(39)	-0.2(3)
C(25)-C(37)-C(38)-C(39)	-178.55(19)
C(37)-C(38)-C(39)-C(40)	0.5(3)
C(38)-C(39)-C(40)-C(41)	-0.6(4)
C(39)-C(40)-C(41)-C(42)	0.3(4)
C(40)-C(41)-C(42)-C(37)	0.0(4)
C(38)-C(37)-C(42)-C(41)	-0.1(3)
C(25)-C(37)-C(42)-C(41)	178.3(2)
C(28)-C(27)-C(43)-C(44)	-58.4(3)
C(26)-C(27)-C(43)-C(44)	119.8(2)
C(28)-C(27)-C(43)-C(48)	123.5(2)
C(26)-C(27)-C(43)-C(48)	-58.3(3)
C(48)-C(43)-C(44)-C(45)	0.8(3)
C(27)-C(43)-C(44)-C(45)	-177.3(2)
C(43)-C(44)-C(45)-C(46)	-0.4(4)
C(44)-C(45)-C(46)-C(47)	-0.3(5)
C(45)-C(46)-C(47)-C(48)	0.7(5)
C(46)-C(47)-C(48)-C(43)	-0.4(4)
C(44)-C(43)-C(48)-C(47)	-0.4(3)
C(27)-C(43)-C(48)-C(47)	177.7(2)

Symmetry transformations used to generate equivalent atoms:
